# Decorating titania with ultrasmall UiO-66-H crystallites enables quantitative photocatalytic oxidation of methane to oxygenates

**DOI:** 10.1038/s41467-026-72422-8

**Published:** 2026-04-29

**Authors:** Geqian Fang, Nour Alhajjar, Wenjun Yu, Maya Marinova, Karima Ben Tayeb, Jian Lin, Thomas Roland, Pardis Simon, Vincent De Waele, Vitaly V. Ordomsky, Andrei Y. Khodakov

**Affiliations:** 1 Université de Lille, CNRS, Centrale Lille, Univ. Artois, UMR 8181—UCCS—Unité de Catalyse et Chimie du Solide, Lille, France; 2 Université de Lille, CNRS, UMR 8516—LASIRe—Laboratoire de Spectroscopie pour les Interactions, la Réactivité et l’Environnement, Lille, France; 3 CAS Key Laboratory of Science and Technology on Applied Catalysis, Dalian Institute of Chemical Physics, Chinese Academy of Sciences, Dalian, China; 4 UMET–Institut Michel-Eugène Chevreul, Université de Lille, CNRS, INRAE, Centrale Lille, Université d’Artois, Lille, France

**Keywords:** Photocatalysis, Organic-inorganic nanostructures, Materials for energy and catalysis

## Abstract

Selective photocatalytic oxidation of methane to liquid oxygenates under ambient conditions remains challenging due to the inert C–H bond and propensity for over-oxidation. Controlling carrier transfer and reactive oxygen species is therefore essential to the selective methane photooxidation. Ultrasmall zirconium metal–organic framework (UiO-66-H) nanocrystals in situ grown on titania form a heterojunction that promotes efficient charge separation and tunes the interfacial band alignment. Comprehensive experiments and characterization reveal that this heterojunction precisely regulates ^•^OH and ^•^OOH generation, enabling controlled, radical-mediated oxidation of methane with a competitive oxygenate yield and nearly 100% selectivity at room temperature using air as an oxidant. In this work, ultrasmall metal–organic framework–semiconductor heterojunctions with a built-in electric field provide an effective route for developing efficient, low-cost photocatalysts for methane chemical valorization under mild, solar-driven conditions.

## Abstract (web summary)

Ultrasmall MOF nanocrystals grown on titania create a heterojunction that controls reactive oxygen species and enhances charge separation, enabling quantitative photocatalytic oxidation of methane to liquid oxygenates under mild conditions.

## Introduction

Methane constitutes the primary component of natural gas, shale gas, biogas, and methane hydrate^1–3^. Methane is a potent greenhouse gas, but converting it into oxygenates such as formaldehyde turns it into platform molecules for synthesis of resins, polymers, and other materials^4^. This direct methane upgrade to chemicals not only creates value from stranded or renewable methane but also offers a cleaner, more efficient route than traditional syngas-based processes. However, it remains difficult to realize a significant conversion of methane due to the inherently small polarizability (2.84 × 10^−40^ C^2^ m^2^ J^−1^), ionization potential (12.61 eV), and high dissociation energy of C–H bonds (440 kJ mol^−1^)^5–9^. Meanwhile, deep oxidation of methane-derived oxygenates to CO_2_ is a more thermodynamically favorable process. Therefore, the partial methane oxidation with a selectivity close to 100% under mild conditions is considered as a major challenge^10^.

Photocatalysis offers a sustainable strategy for methane conversion by leveraging solar energy to drive chemical transformations under mild conditions^11^. Selective photocatalytic CH_4_ oxidation remains limited by two interrelated problems: inefficient charge separation and poorly controlled radical flux that drives methane over-oxidation to CO_2_^12^. Recent reports have explored integration of metal oxide semiconductors (e.g., ZnO, TiO_2_, WO_3_) with noble metal catalysts (e.g., Au, Pt, Pd) at the nano- or atomic scale to form Schottky barriers^12–15^. Through redox reactions with oxygen donors such as water and O_2_, this configuration enables the in situ generation of reactive oxygen species (ROS), including hydroxyl radicals (^•^OH) and hydroperoxyl radicals (^•^OOH), thereby enhancing photocatalytic methane hydroxylation. However, conventional semiconductors suffer from inherent drawbacks, such as limited charge-carrier mobility, rapid electron–hole recombination, and uncontrolled ROS generation, which undermine their photocatalytic performance. Furthermore, the high cost and scarcity of noble metals severely hinder the scalability of these systems. Thus, the development of highly selective and low-cost photocatalysts for enhancing methane oxidation deserves further investigation.

Heterojunction photocatalysts without noble metals are emerging as cost-effective systems that enhance charge separation, accelerate interfacial transfer, and leverage cocatalysts for high activity and selectivity^16,17^. Yet, conventional all-inorganic heterojunctions remain underwhelming for selective CH_4_ oxidation: their rigid band structures limit control over redox potentials and charge-transfer pathways, hindering the concurrent attainment of high activity and product selectivity. Zirconium-based metal-organic frameworks (UiO-66 MOF) are attractive not only as standalone photocatalysts but, crucially, as cocatalysts/promoters for important semiconductors, such as TiO_2_. When dispersed as ultrasmall nanocrystals, they can act as porous, Lewis-acidic surface modifiers that tailor interfacial band bending and electric fields, passivate recombination sites, and provide selective adsorption/activation pockets (for H_2_O/O_2_ and reactants), thereby enhancing charge separation, steering surface chemistry and catalytic properties. Their high surface area, hydrothermal robustness^18–22^, and modular ligand chemistry enable fine tuning of TiO_2_ interfacial energetics without compromising light harvesting. Downsizing to sub-2 nm dramatically shortens carrier-transport pathways and maximizes perimeter contact with TiO_2_, thereby achieving rate and selectivity enhancements comparable to those typically induced by promoter addition^23^. Despite these advantages, synthetic constraints have so far precluded sub-2 nm Zr-MOF being deliberately implemented as photocatalytic promoters on TiO_2_.

Here, we construct a (U6-H)/TiO_2_ heterojunction with a robust built-in electric field (BEF) by in situ growth of ultrasmall MOF nanocrystals on the TiO_2_ surface. Ultrasmall (sub-2 nm, abbreviated as S2) UiO-66-H nanocrystals decorate and remodel TiO_2_ surface sites (–OH, Ti^3+^/O_v_) and create Zr–O–Ti bridges. This highly efficient heterojunction enhances charge carrier management and modulates ROS production by shifting interfacial band offsets. Consistent with this mechanism, the optimized 5(U6-H)_S2_/TiO_2_ photocatalyst exhibits nearly 100% selectivity toward oxygenates and achieves an impressive oxygenate yield of 6454 μmol g_cat_⁻¹ h⁻¹ at room temperature using air as an oxidant.

## Results

### Synthesis and characterization

**Fig. 1:**
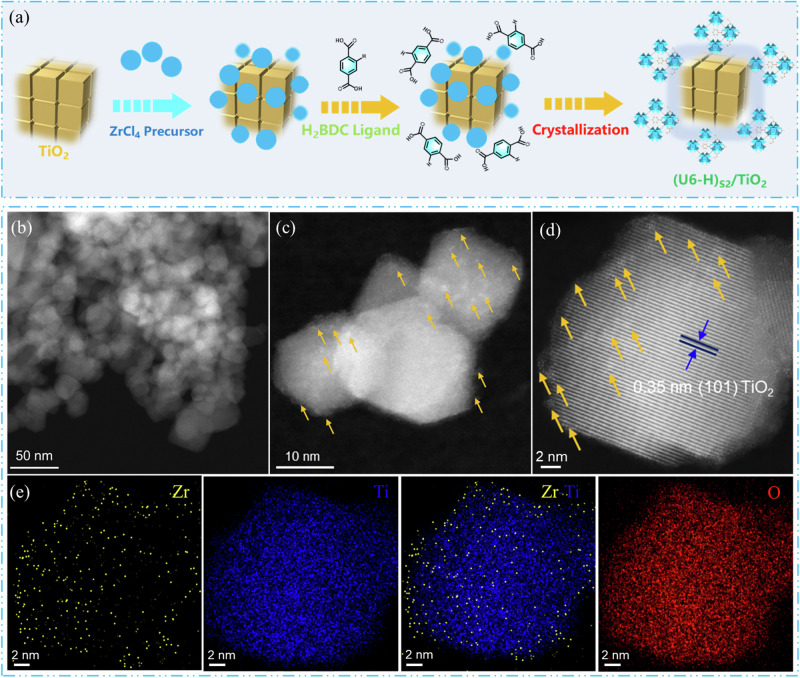
Synthesis and structural characterization of (U6-H)_S2_/TiO_2_ heterostructure. **a** Schematic illustration of TiO_2_ anchored sub-2 nm U6-H via in situ growth method (light orange cubes: TiO_2_, blue spheres: Zr precursor species, green molecular structures: H_2_BDC ligands, and cyan polyhedral units: U6-H MOF). **b** Representative AC-HAADF-TEM. **c**, **d** High resolution AC-HAADF-TEM (the yellow arrows, the blue arrows and black lines pointing to MOF species and lattice spacing, respectively) and corresponding EDS-Mapping of 5(U6-H)_S2_/TiO_2_ catalyst (**e**).

The UiO-66-H/TiO_2_ composite (simplified to (U6-H)/TiO_2_) was prepared via an in situ growth strategy (Fig. 1a). TiO_2_ (P25) was first mixed with ZrCl_4_ in DMF under stirring to form oxophilic Zr^4+^ coordinated TiO_2_. Subsequently, terephthalic acid (H_2_BDC) ligand was added into the above mixture to produce the (U6-H)/TiO_2_ composite under the hydrothermal conditions. By varying the amount of precursor, (U6-H)/TiO_2_ composites with U6-H contents in the range of 2.5–20 wt% were achieved, denoted as x(U6-H)/TiO_2_ (e.g., 5(U6-H)/TiO_2_). The powder X-ray diffraction (XRD) patterns of U6-H, TiO_2_, and (U6-H)/TiO_2_ composite materials are shown in Supplementary Fig. 1. Pristine U6-H exhibits two prominent diffraction peaks at 2θ = 7.5° and 8.6°, corresponding to the (111) and (200) crystal planes, respectively^24,25^. P25 TiO_2_ exhibits two prominent diffraction peaks at 25.3° and 27.4°, corresponding to the anatase (101) and rutile (100) planes, confirming the presence of anatase and rutile phases^26^. In the (U6-H)/TiO_2_ composites, the characteristic peaks of U6-H gradually increase with the U6-H content, confirming the coexistence of both U6-H and TiO_2_ phases.

Aberration corrected high angle angular dark field-scanning transmission electron microscopy (AC-HAADF-TEM) was conducted to further explore the microstructure of TiO_2_ and U6-H. Figure 1b, c, and Supplementary Fig. 2 show that both 5(U6-H)_S2_/TiO_2_ and TiO_2_ exhibit an aggregated nanoparticles morphology with an average size of ~20 nm. The observed lattice fringes with a d-spacing of 0.35 nm correspond to the (101) plane of anatase TiO_2_^27^, which is consistent with the XRD results (Supplementary Fig. 1). Multiple high-resolution AC-HAADF-TEM images further reveal that ultra-small MOF nanocrystals with a size of approximately <2 nm are anchored on the surface of TiO_2_ (Fig. 1c, d and Supplementary Fig. 3). Moreover, energy-dispersive X-ray spectroscopy mapping (EDX-mapping) confirms that the Zr element from (U6-H)_S2_ is uniformly dispersed across the TiO_2_ surface (Fig. 1e). The intimate contact between TiO_2_ and sub-2 nm U6-H is likely to enhance the transfer of photogenerated carriers and mitigate the recombination of electron-hole pairs. The specific surface area and texture of the synthesized samples were also evaluated by N_2_ physical adsorption measurements at −196 °C. Supplementary Fig. 4 and Supplementary Table 1 show a hierarchical porous structure of 5(U6-H)_S2_/TiO_2_ composed of both micropores and mesopores (0.6–15 nm). Compared to pure TiO_2_, the specific surface area of 5(U6-H)_S2_/TiO_2_ significantly increases with higher loading of U6-H, from 54.3 to 261.9 m^2^/g. The well-defined MOF porous structure enables precise tuning of the MOF–oxide interface on (U6-H)_S2_/TiO_2_.

**Fig. 2:**
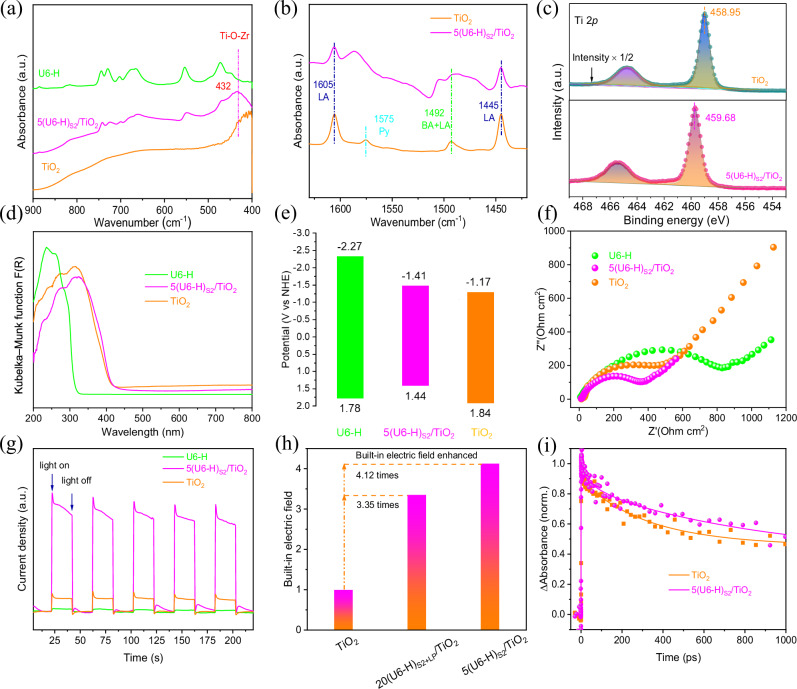
Electronic structure and charge transfer properties of (U6-H)_S2_/TiO_2_. **a** ATR-IR spectra on U6-H, TiO_2_ and 5(U6-H)_S2_/TiO_2_ catalysts (dotted lines indicating Ti-O-Zr bond position), **b** Pyridine-IR spectra on TiO_2_ and 5(U6-H)_S2_/TiO_2_ catalysts; Py: pyridine; LA: Lewis acid; BA: Brønsted acid, (dotted lines indicating Py adsorption IR bands), **c** Ti 2*p* XPS spectra on TiO_2_ and 5(U6-H)_S2_/TiO_2_ catalysts; **d** diffuse reflectance UV-Vis spectra; **e** energy structure diagram; **f** EIS spectra; The solution resistance (Rs), obtained from the high-frequency intercept of the Nyquist plots, was approximately 19.0, 17.3, and 24.7 Ω cm^2^ for TiO_2_, 5(U6-H)_S2_/TiO_2_, and U6-H, respectively. No iR correction was applied in this study. **g** the photocurrent density with light on/off cycles on U6-H, TiO_2_ and 5(U6-H)_S2_/TiO_2_ catalysts, **h** the BEF intensity for TiO_2_, 5(U6-H)_S2_/TiO_2_ and 20(U6-H)_S2+LP_/TiO_2_ catalysts (orange arrows indicating enhanced times), **i** Pump-probe decay curves recorded for 5(U6-H)_S2_/TiO_2_ and TiO_2_. The marks correspond to the experimental and the full-line to their fit, respectively.

Attenuated total reflectance infrared (ATR-IR) spectroscopy provided deeper insight into the interaction between TiO_2_ and U6-H. With the introduction of an ultrasmall U6-H nanocrystals, a new characteristic band at 432 cm^−1^ was observed (Fig. 2a), suggesting the formation of Ti–O–Zr chemical bond^28,29^. These observations indicate that the hard oxophilic Zr^4+^ cations of the MOF are successfully coordinated with surface oxygen of the TiO_2_ nanoparticles, forming a robustly interfacial contact between TiO_2_ and U6-H. Pyridine-adsorption IR (Py-IR) was used to characterize Lewis-acid surface sites of TiO_2_, U6-H and 5(U6-H)_S2_/TiO_2_ catalysts. As shown in Fig. 2b, the characteristic bands of pyridine coordinated to Lewis acid Ti^δ+^ sites on TiO_2_ (1445 and 1605 cm⁻¹) are markedly weakened after the introduction of ultrasmall U6-H nanocrystals. This observation indicates a significant decrease in the density of accessible TiO_2_-based Lewis acid sites, consistent with their involvement in interfacial coordination during heterojunction formation^30^. In contrast, pristine U6-H exhibits relatively weaker pyridine adsorption bands at around 1438 and 1605 cm^−1^ (Supplementary Fig. 5), which are assigned to pyridine coordinated to Zr^δ+^ Lewis acid sites associated with Zr-oxo nodes^31^. The overall weak intensity of these bands suggests a relatively low density of intrinsic Lewis acid sites in U6-H, confirming that the observed suppression of Lewis acidity in 5(U6-H)_S2_/TiO_2_ primarily originates from the modification and blocking TiO_2_ surface sites.

The electronic interaction between U6-H and TiO_2_ in (U6-H)_S2_/TiO_2_ was also investigated using X-ray photoelectron spectroscopy (XPS). Compared with pristine TiO_2_, the Ti 2*p* peak of 5(U6-H)_S2_/TiO_2_ exhibits a shift to higher binding energy, from 458.95 to 459.68 eV (Fig. 2c), indicating interfacial electronic coupling and charge redistribution associated with a heterojunction. In contrast, the Zr 3d peaks of 5(U6-H)_S2_/TiO_2_ remain within the characteristic binding energy range of Zr^4+^ species in U6-H (Supplementary Fig. 6). Low-temperature (100 K) solid-state electron paramagnetic resonance (EPR) spectra reveal a stronger [Ti^3+^---O^-^] signal for 5(U6-H)_S2_/TiO_2_ compared with TiO_2_ (Supplementary Fig. 7), suggesting that the chemically coupled U6-H-TiO_2_ interface promotes the formation and stabilization of oxygen-vacancies and facilitates charge separation.

The ultraviolet–visible spectroscopy (UV-Vis) spectra of pure TiO_2_, U6-H, and the 5(U6-H)_S2_/TiO_2_ composite are shown in Fig. 2d. Compared to pristine U6-H, the 5(U6-H)_S2_/TiO_2_ composite exhibits enhanced absorption in the near UV region, with a slightly extended absorption range than TiO_2_. This enhancement can be attributed to the interfacial interaction between TiO_2_ and ultrasmall U6-H nanocrystals. The band gap energies (E_g_) were calculated using Tauc plot (Supplementary Fig. 8**)**. The bandgap energy (E_g_) of the 5(U6-H)_S2_/TiO_2_ composite (2.85 eV) is smaller than that of both TiO_2_ (3.01 eV) and U6-H (4.05 eV). Furthermore, ultraviolet photoemission spectroscopy (UPS) and XPS were carried out to investigate the band position of TiO_2_, U6-H, and (U6-H)_S2_/TiO_2_ catalysts (Supplementary Figs. 9 and 10). After referencing the energy levels to the NHE scale using the measured work function (Fig. 2e and Supplementary Table 2), the valence band maximum (VBM) of (U6-H)_S2_/TiO_2_ is 1.44 eV versus NHE; its oxidation potential is significantly lower than that of TiO_2_ (VBM ~1.84 eV versus NHE) and U6-H (VBM ~1.78 eV versus NHE). This indicates strong interfacial coupling and the formation of a heterojunction between TiO_2_ and ultrasmall sub-2 nm U6-H. The heterojunction is expected to facilitate charge separation and to enhance the photocatalytic performance of the (U6-H)_S2_/TiO_2_ composite.

Steady-state photoluminescence (PL, Supplementary Fig. 11) was conducted to investigate the influence of designed structure on the charge carrier separation. U6-H exhibited two strong emission bands of fluorescence at 385 and 445 nm. After decorating the TiO_2_ surface with sub-2 nm MOF species, the PL emission spectrum of 5(U6-H)_S2_/TiO_2_ exhibited a single peak similar to pure TiO_2_ at 460 nm with a relatively low luminescence intensity. The lower intensity of the PL emission of 5(U6-H)_S2_/TiO_2_ reflects a lower recombination rate of charge carriers because of the electronic coupling between the (U6-H)_S2_ and TiO_2_. This implies that the reduced recombination is due to the introduction of ultrasmall U6-H nanocrystals to form junction interfaces. Moreover, electrochemical impedance spectroscopy (EIS) (Fig. 2f) shows that 5(U6-H)_S2_/TiO_2_ has the highest charge transfer efficiency, as indicated by its smallest arc radius. To further unveil the charge migration and transfer efficiency in the composite, photocurrent analyses are performed (Fig. 2g). Compared with pure TiO_2_ and U6-H, 5(U6-H)_S2_/TiO_2_ exhibits a higher photocurrent intensity, indicating that the intimate interface between the two components enhances the separation and transfer efficiency of photoexcited electron–hole pairs. As displayed in Supplementary Figs. 12, 13, the surface charge density of 5(U6-H)_S2_/TiO_2_ was calculated to be higher than that of TiO_2_ (58.0 vs 3.98 μC cm^−2^). Notably, 20(U6-H)_S2+LP_/TiO_2_ also exhibits an enhanced surface charge density of 41.6 μC cm^−2^ (Supplementary Fig. 14), which remains much higher than that of bare TiO_2_, indicating effective photogenerated charge accumulation induced by the heterojunction. 5(U6-H)_S2_/TiO_2_ composite displayed a stronger surface photogenerated voltage (SPV) response (Supplementary Fig. 15) than pure TiO_2_ (49.0 vs 42.0 mV), while 20(U6-H)_S2+LP_/TiO_2_ shows a comparable but slightly lower SPV value (45.0 mV), reflecting efficient charge separation in both heterostructured catalysts. In the light of photocurrent and SPV results, the BEF intensity of 5(U6-H)_S2_/TiO_2_ was calculated to be ~4.12 times higher than that of bare TiO_2_ (Fig. 2h), whereas 20(U6-H)_S2+LP_/TiO_2_ exhibits a ~3.35 enhanced BEF, further confirming a positive role of ultrasmall U6-H domains in strengthening the interfacial BEF.

Femtosecond transient absorption in the mid-infrared measurements (Mid-IR fs-TAS) has been carried out to characterize the decay of the free and shallow trapped electron populations generated upon photoexcitation of TiO_2_ and 5(U6-H)_S2_/TiO_2_ under UV-light excitation (Fig. 2i and Supplementary Figs. 16–18). Both pump-probe decay curves exhibit a similar qualitative evolution. The population of the free/shallow-trapped electrons is formed instantaneously with a rise time less than 200 fs^32,33^. The intensity of the signal is stable during the first 10 ps, and then the amplitude of the traces decreases and finally reaches a plateau at 1 ns (Supplementary Fig. 18), which is typical of the evolution of the population of electron escaping the geminate recombination^34–36^. Fig. 2i shows that the decay kinetics of 5(U6-H)_S2_/TiO_2_ are slower and that the intensity of the signal at 1 ns is significantly higher than for TiO_2_. Sub-2 nm U6-H changes the electron-population lifetime, consistent with more electrons avoiding geminate electron–hole recombination. Therefore, the Mid-IR fs-TAS data suggest that the charge separation is more efficient in 5(U6-H)_S2_/TiO_2_ than for TiO_2_. We observe a ~10–15% higher fraction of electrons and holes separated at 1 ns in 5(U6-H)_S2_/TiO_2_ vs TiO_2_. In comparison, pure U6-H exhibits no transient signal assigned to the formation of shallow trapped electrons (Supplementary Fig. 19). These results reveal a strong interfacial coupling between U6-H and TiO_2_, which alters the electronic structure of both constituents, establishes a robust BEF with improved charge separation, and drives chemical interactions leading to Ti–O–Zr interface formation. The incorporation of sub-2 nm U6-H on TiO_2_ facilitates the formation of an organic-inorganic hybrid MOF-oxide heterojunction, which significantly promotes charge separation.

### Photocatalytic methane oxidation

**Fig. 3:**
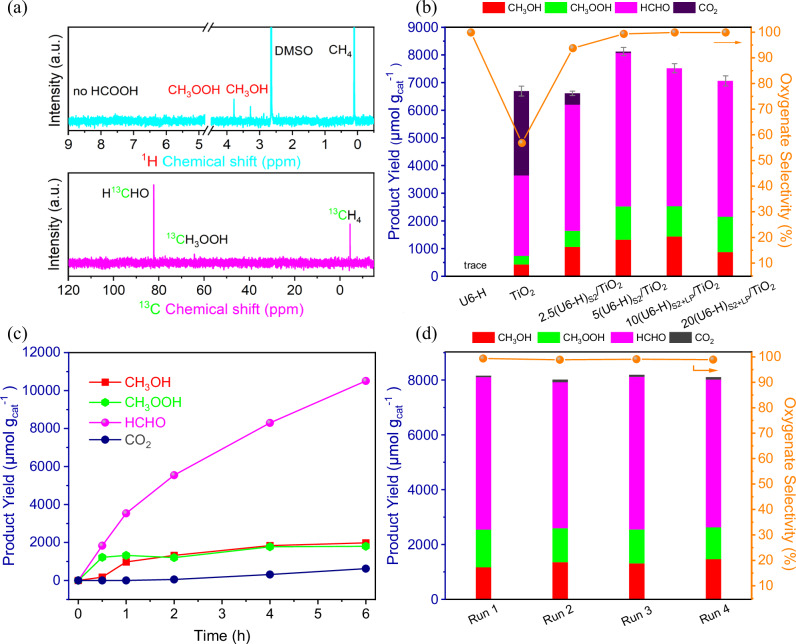
Photocatalytic performance in methane oxidation. Test conditions: Pressure (CH_4_) = 18 bar, (Air) = 2 bar, Catalyst: 5 mg 5(U6-H)_S2_/TiO_2_, 100 mL H_2_O, r.t., 500 W Xe lamp (350 mW cm^−1^). **a** ^1^H NMR spectrum of liquid products over (U6-H)_S2_/TiO_2_, illumination 2 h; ^13^C NMR spectrum of reaction products from ^13^CH_4_ oxidation over (U6-H)_S2_/TiO_2_. Test conditions: Pressure: (^13^CH_4_) = 7 bar, (Air) = 1 bar, Catalyst: 5 mg 5(U6-H)_S2_/TiO_2_, 100 mL H_2_O, r.t., illumination 4 h. **b** Yield and selectivity of oxygenates in the photocatalytic methane oxidation over various (U6-H)/TiO_2_ catalysts, illumination 2 h. **c** Time dependence of oxygenate production during the photocatalytic methane oxidation over 5(U6-H)_S2_/TiO_2_. **d** Reusability of 5(U6-H)_S2_/TiO_2_ in the photocatalytic methane oxidation over multiple cycles, illumination 2 h, respectively.

Methane photocatalytic oxidation was carried out in air under irradiation from a 500 W Xenon (Xe) lamp at room temperature (Supplementary Fig. 20). The products were identified and quantified by ^1^H and ^13^C nuclear magnetic resonance (^1^H NMR; ^13^C NMR) (Fig. 3a), UV-Vis spectroscopy, and gas chromatography (GC) (Supplementary Figs. 21–23). Figure 3b shows the yields of oxygenates (HCHO, CH_3_OH, and CH_3_OOH) over the photocatalysts, along with CO_2_, which is the complete oxidation product. CO was not detected among the reaction products. Pristine U6-H exhibits trace yields of oxygenates, consistent with its limited light absorption and severe charge recombination. TiO_2_ demonstrates an increased production of oxygenates, but with a poor selectivity (55%). After introduction of highly dispersed sub-2 nm MOF nanocrystals onto the TiO_2_ surface via an in-situ growth method, the oxygenate yield increased markedly, while CO_2_ formation was effectively suppressed. The 5(U6-H)_S2_/TiO_2_ sample exhibits the highest oxygenate yield, reaching 8069.4 μmol g_cat_^−1^ within 2 h, accompanied by a high selectivity of 99.3%. The yield is 2.3 times higher than that of pure TiO_2_, However, further increase in the U6-H content leads to a decline in the oxygenate yield. AC-TEM (Supplementary Fig. 24) and PL (Supplementary Fig. 25) analysis indicate that higher precursor concentrations promote larger MOF growth on TiO_2_, reducing charge-diffusion efficiency and, consequently, catalytic activity. Notably, the performance of the optimized 5(U6-H)_S2_/TiO_2_ catalyst is highly competitive with most state-of-the-art photocatalysts, even under higher O_2_ concentrations or higher irradiance intensity (Supplementary Table 3).

Figure 3c shows a time dependence of product yield over the optimal 5(U6-H)_S2_/TiO_2_ catalyst, which possesses a maximized Zr–O–Ti interface. The data show that HCHO is the dominant product, exhibiting continuous accumulation throughout the reaction. To investigate the stability of the optimized 5(U6-H)_S2_/TiO_2_ photocatalyst, several reaction cycles were carried out (Fig. 3d). No obvious decrease in oxygenate yield and selectivity was observed after 4 cycles, demonstrating good stability of 5(U6-H)_S2_/TiO_2_. Meanwhile, XRD patterns and ATR-IR spectra of used 5(U6-H)_S2_/TiO_2_ remain nearly identical to those of the fresh samples, confirming the catalyst’s stable crystalline and heterojunction structures (Supplementary Figs. 26 and 27).

### Mechanistic investigation

**Fig. 4:**
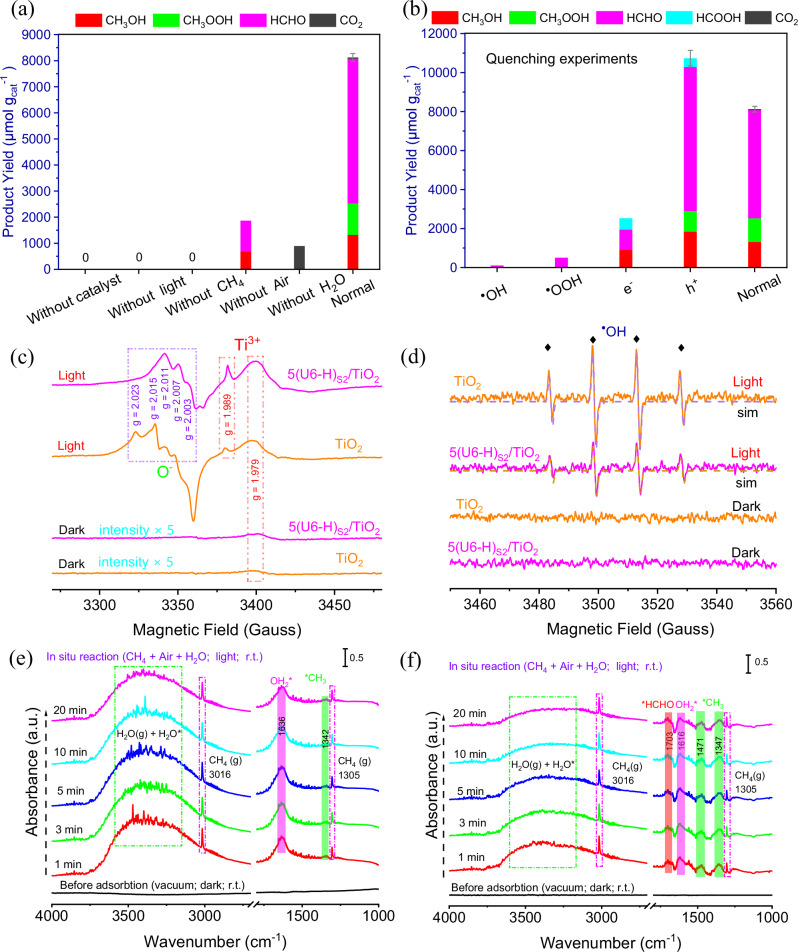
Mechanistic investigation. **a** Blank experiments; **b** quenching experiments on 5(U6-H)_S2_/TiO_2_ catalyst. The *x*-axis shows the quenched species. Test conditions: Pressure (CH_4_) = 18 bar and/or (Air) = 2 bar, Catalyst: 5 mg 5(U6-H)_S2_/TiO_2_ and/or 100 mL H_2_O, r.t., 500 W Xe lamp (350 mW cm^−1^) illumination 2 h; K_2_S_2_O_8_, Na_2_C_2_O_4_, salicylic acid, and 1,4-benzoquinone were used as quenchers for e^-^, h^+^, ^•^OH, and ^•^OOH, respectively. **c** in situ solid EPR spectra under low temperature (100 K) with CH_4_ and air (dashed boxes indicating EPR signal); **d** in situ liquid EPR spectra with DMPO as spin trapping of ^•^OH with CH_4_ and air on 5(U6-H)_S2_/TiO_2_ and TiO_2_ catalysts (dashed line indicating radical sim. signal), In situ FT-IR spectra with CH_4_ and air on (**e**) TiO_2_ and **f** 5(U6-H)_S2_/TiO_2_ catalysts (dashed boxes and highlight area indicating adsorbed species signal).

Blank (control) experiments were conducted by omitting, in turn, the catalyst, light, methane, or air (Fig. 4a). In all these experiments, no oxygenates were detected, thus confirming that the reaction is indeed a photocatalytic CH_4_ oxidation driven by 5(U6-H)_S2_/TiO_2_. Remarkably, in the absence of liquid H_2_O in the reactor, CH_4_ undergoes full oxidation to CO_2_. This underscores significance of the liquid phase as a crucial factor for preventing over-oxidation. The conversion of labelled ^13^CH_4_ resulted in a high concentration of ^13^C-labeled oxygenates (Fig. 3a**)**, with H^13^CHO and ^13^CH_3_OOH clearly detected by ^13^C NMR at approximately ~82 and ~65 ppm, respectively^37^. These results prove that the oxygenated products derive from CH_4_ oxidation rather than the catalyst itself. Notably, a high apparent quantum yield (AQY) of 6.77% ± 0.34 was achieved on 5(U6-H)_S2_/TiO_2_ under monochromatic irradiation with a wavelength of 352 nm (Supplementary Table 4). In contrast, the calculated AQY under 418 nm light is zero, consistent with the UV–vis absorption spectrum of 5(U6-H)_S2_/TiO_2_ (Fig. 2d). The negligible oxygenate formation under *λ* > 400 nm confirms insignificant contribution from Vis/IR irradiation, including possible thermal effects (Supplementary Fig. 28).

Quenching experiments were conducted to identify the active species involved in the methane oxidation over 5(U6-H)_S2_/TiO_2_. Figure 4b shows that the reaction was nearly completely suppressed upon the addition of salicylic acid and 1, 4-benzoquinone, quenchers for ^•^OH and ^•^OOH, respectively^38^. This suggests that the process proceeds predominantly through a radical-mediated mechanism. When K_2_S_2_O_8_ was introduced to trap photogenerated electrons^26^, the reduction of O_2_ to protonated ^•^OOH, H_2_O_2_, and then to ^•^OH species via the Haber-Weiss and/or Fenton-like multistep process was significantly hindered, resulting in a sharp decline in catalytic activity^39^. Importantly, upon the simultaneous addition of a small amount of H_2_O_2_ (40 μL) together with K_2_S_2_O_8_ (Supplementary Fig. 29), the photocatalytic activity was largely restored, indicating that externally supplied H_2_O_2_ can bypass the electron-dependent O_2_ activation pathway and directly generate ^•^OH species through a Fenton-like process. In contrast, upon the addition of Na_2_C_2_O_4_ as a hole sacrificial agent, the proton-coupled electron transfer (PCET) pathway for O_2_ reduction is significantly promoted, leading to a remarkable enhancement in the photocatalytic performance^40^. Notably, when methane was replaced with N_2_ as the feed gas, no products were detected. However, when both the h⁺ and ^•^OOH scavengers were introduced, the yield of oxygenates significantly decreased, indicating that the enhanced production of oxygenates originates from ROS–mediated methane activation.

The active species on different catalysts under reaction conditions were further investigated by in situ solid/liquid EPR spectroscopy. Figure 4c shows that, both of TiO_2_ and 5(U6-H)_S2_/TiO_2_ generate the [Ti^3+^---O^−^] species under illumination^41–43^. The O^-^ signals observed on TiO_2_ exhibit a rhombic-type pattern, which can be attributed to hole-trapped oxygen species on the anatase and/or rutile phases of P25^44,45^. After the introduction of U6-H, the reduction of paramagnetic signals’ intensity can be attributed to the formation of Zr–O–Ti interfaces, which alter the coordination environment of the O centers with Zr^4+^ and Ti^δ+^^46^, consistent with the ATR-IR results (Fig. 2a). Notably, 5(U6-H)_S2_/TiO_2_ produced more stronger signals of Ti^3+^ (*g* = 1.979 and 1.989) than pristine TiO_2_ under irradiation^47^. These findings indicate that a higher concentration of Ti^3+^ sites acting as electron acceptors captures photogenerated electrons from the VBM of TiO_2_ decorated with highly dispersed sub-2 nm U6-H. This process can facilitate the formation of more electron-rich active sites and effectively modulates the generation of moderate ^•^OOH species via PCET process, thereby suppressing the undesirable over-oxidation paths.

In situ liquid EPR tests were further carried out by using 5, 5-dimethyl-1-pyrroline N-oxide (DMPO) as a spin trap^1^. Before the measurements, the photocatalyst was dispersed into a solution of DMPO under atmosphere of CH_4_ and air. As shown in Fig. 4d, a quartet signal peak of spin adducts DMPO-^•^OH appears after irradiation both on TiO_2_ and (U6-H)_S2_/TiO_2_. Evidently, the ^•^OH signal intensity for (U6-H)_S2_/TiO_2_ is almost 2 times weaker than that for TiO_2_ (Supplementary Table 5)^48^. This observation is consistent with the VBM of (U6-H)_S2_/TiO_2_ shift to a lower level compared to TiO_2_ (∼1.44 eV vs. ∼1.84 eV versus NHE, Fig. 2e) after the introduction of sub-2 nm U6-H, thereby weakening its ability to oxidize H_2_O into ^•^OH^49^. The decrease in ^•^OH concentration coincides with an increase in the selectivity toward oxygenates and a decrease in the selectivity toward CO_2_. When water was replaced by methanol^37^, ^•^OOH was successfully detected by in situ liquid EPR (Supplementary Figs. 30 and 31). Notably, the integral area of ^•^OOH on 5(U6-H)_S2_/TiO_2_ is 2.3 times higher than that on TiO_2_, indicating favorable generation of ^•^OOH on (U6-H)_S2_/TiO_2_ (Supplementary Fig. 32 and Supplementary Table 6). The enhanced ^•^OOH formation originates from the shift of the CBM in 5(U6-H)_S2_/TiO_2_ to a more negative potential (1.41 eV versus NHE) compared with pristine TiO_2_ (1.17 versus NHE, Fig. 2e), together with a higher concentration of Ti^3+^ species (Fig. 4c). Both factors facilitate the reduction of O_2_ to ^•^O_2_⁻, followed by protonation to ^•^OOH. Consequently, this pathway directly correlates with the predominance of CH_3_OOH and HCHO in the products. These results demonstrate that the (U6-H)_S2_/TiO_2_ catalyst preferentially generates moderately reactive ^•^OOH intermediates while suppressing strong ^•^OH oxidizing species, thereby optimizing the product distribution.

Considering that methanol is one of the key intermediates in the formation of formaldehyde, we used CH_3_OH as a reactant instead of CH_4_ to evaluate the catalyst’s capacity to hinder the over-oxidation. Supplementary Fig. 33 shows that, in CH_3_OH oxidation, (U6-H)_S2_/TiO_2_ achieves a much higher selectivity for oxygenates than pure TiO_2_. Thus, forming the (U6-H)_S2_/TiO_2_ heterojunction and decorating TiO_2_ with sub-2 nm MOF nanoparticles modulate catalyst electronic structure and surface sites, thereby suppressing the overproduction of ^•^OH radicals.

In photocatalytic systems, ^•^OOH and ^•^OH can be formed via two pathways: (i) oxidation of H_2_O or surface hydroxyl groups by photogenerated holes in the VB, and (ii) reduction of O_2_ at the conduction band to ^•^O_2_^−^, followed by protonation to ^•^OOH or H_2_O_2_ and subsequent Fenton-like reactions yielding ^•^OH^12^. A combination of in situ measurements and quenching experiments suggests that ^•^OOH and ^•^OH are primarily produced over (U6-H)_S2_/TiO_2_ via the second pathway. The regulated concentration of ROS via a tuneable band structure ultimately contributes to the enhanced selectivity of photocatalytic methane hydroxylation and reduces oxygenate over-oxidation to CO_2_.

Furthermore, in situ Fourier transform infrared (in situ FTIR) spectroscopy was employed to monitor the adsorption states of reactants and intermediates during the photocatalysis. Both TiO_2_ and 5(U6-H)_S2_/TiO_2_ exhibit (Fig. 4e, f) under illumination characteristic bands of weakly adsorbed CH_4_ (1305, 1342 and 3016 cm^−1^)^50^. In contrast to pristine TiO_2_, (U6-H)_S2_/TiO_2_ displays additional bands at 1471 cm^−1^ and 1703 cm^−1^ ^51^, possibly assigned to surface methoxides or formates, which intensity correlates with high HCHO productivity (Fig. 2a). Following gas cutoff and subsequent evacuation at 150 °C, all signals vanish (Supplementary Figs. 34 and 35), confirming that the detected carbon species originate exclusively from CH_4_ conversion rather than the catalyst itself. Thus, constructing the (U6-H)_S2_/TiO_2_ heterojunction and decorating TiO_2_ with MOF nanoparticles jointly modulate the electronic structure and surface sites, thereby enabling controlled generation of ROS and ultimately achieving selective oxidation of methane.

**Fig. 5:**
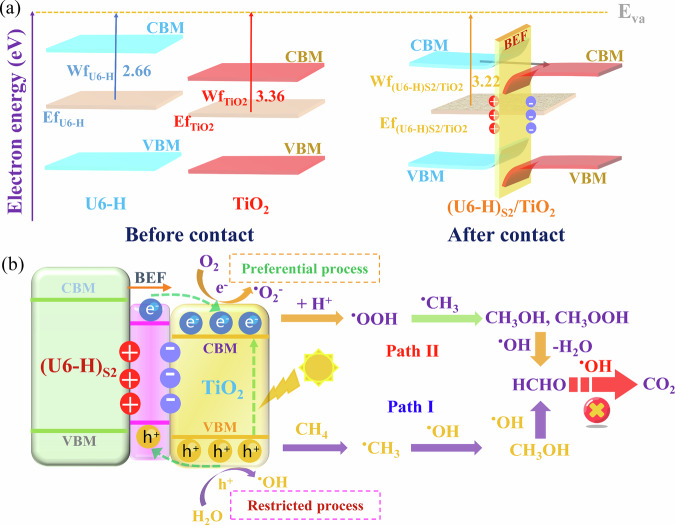
Proposed mechanism. **a** Sketches of U6-H and TiO_2_ before/after contact. **b** Plausible mechanism for the photocatalytic oxidation of CH_4_ to oxygenates over (U6-H)_S2_/TiO_2_ (Wf work function, Ef Fermi level, *E*_vac_ vacuum level, “+”: positive charge, “-”: negative charge, the colored planes: the band position, Fermi level, and BEF).

Combined spectroscopic analyses, particularly UPS, conclusively demonstrate that U6-H possesses a higher Fermi level and a lower work function (2.66 eV) (Supplementary Fig. 9 and Fig. 5a). Upon heterojunction formation, electrons redistribute between U6-H and the Zr–O–Ti interface. This process aligns with their Fermi levels, with TiO_2_ exhibiting a higher work function (3.36 eV). The resulting 5(U6-H)_S2_/TiO_2_ heterostructure displays a reduced work function (3.22 eV) compared with pristine TiO_2_, thereby establishing a robust BEF that facilitates rapid charge transfer. Based on the present findings and previous studies^52^, a plausible mechanism of the photocatalytic oxidation of CH_4_ to oxygenates over (U6-H)_S2_/TiO_2_ is proposed (Fig. 5b). In the (U6-H)_S2_/TiO_2_ composite catalyst, TiO_2_ with a narrower bandgap is mainly responsible for the photon adsorption. The generated electrons eventually migrate from (U6-H)_S2_ to the TiO_2_ surface for reduce O_2_ to generate protonated ^•^OOH and ^•^OH species via Haber-Weiss and Fenton-like reactions (Fig. 4d). The ^•^OH radicals activate CH_4_ to form ^•^CH_3_ intermediates^53,54^, which subsequently react with ^•^OH and ^•^OOH to form CH_3_OH and CH_3_OOH, respectively. It is noteworthy that CH_3_OH and CH_3_OOH can serve as intermediates for HCHO formation through distinct reaction pathways, namely oxidation and dehydration, respectively. The photogenerated holes transfer from the valence band of TiO_2_ to the (U6-H)_S2_/TiO_2_ junction interface, where the Type-II–like band alignment effectively suppresses electron–hole recombination and enhances the photocatalytic activity (Figs. 2e, 5b)^55^. Importantly, the highly dispersed sub-2 nm MOF crystallites on TiO_2_ decrease the VBM and further suppress excessive ^•^OH generation from H_2_O oxidation (Fig. 4d and Supplementary Table 5). At the same time, the more negative CBM favors a selective O_2_ reduction pathway toward mild ^•^OOH species (Supplementary Fig. 31 and Supplementary Table 6). This synergistic modulation of the interfacial energy structure at the MOF–TiO_2_ heterojunction enhances the overall photocatalytic activity while effectively preventing over-oxidation, thus enabling near-complete selectivity toward the desired oxygenates.

## Discussion

We have demonstrated a rational interfacial engineering strategy for constructing a highly efficient MOF–TiO_2_ catalyst, in which sub-2 nm ultrasmall Zr-based MOF nanocrystals (U6-H)_S2_ are uniformly dispersed and firmly anchored on the TiO_2_ surface. The MOF–TiO_2_ interface is formed via in situ MOF growth on the titania surface. This synergistic junction not only promotes efficient charge separation via a robust BEF but also exerts a tunable band structure to precisely control the concentration of ^•^OH and ^•^OOH species, enabling selective activation of methane while suppressing over-oxidation. Decorating TiO_2_ with ultrasmall U6-H nanocrystallites tunes the oxidation potential of photo-holes, thereby suppressing excessive ^•^OH formation via water oxidation. In addition, decorating TiO_2_ with sub-2 nm MOF crystallites modifies the catalytically active surface sites, thereby moderating generation of highly reactive ^•^OH species.

In the (U6-H)_S2_/TiO_2_ composite catalyst, the radicals that promote methane hydroxylation are predominantly generated via an O_2_ PCET pathway, driven by an increased reduction potential. As a result, the optimized 5(U6-H)_S2_/TiO_2_ catalyst achieves a remarkable oxygenate yield of 6454 μmol g_cat_⁻¹ h⁻¹ with nearly 100% selectivity at room temperature using air as the oxidant, demonstrating competitive performance compared with reported photocatalysts. These results highlight the central role of the MOF–TiO_2_ heterojunction in steering both activity and selectivity. A generalizable platform is proposed for sub-2 nm MOF–semiconductor interface design in solar-driven C–H functionalization with high efficiency and molecular precision.

## Methods

### Chemicals

Titanium dioxide (TiO_2_, P25, ≥99.5% trace metals basis), Deuterium oxide (D_2_O, 99.9 atom % D), Dimethyl sulfoxide (DMSO, anhydrous, (≥99.9%), 5, 5-dimethyl-1-pyrroline N-oxide (DMPO, ≥99%), Methanol (CH_3_OH, ≥99.9%), Ethanol (CH_3_CH_2_OH, ≥99.9%), Acetone (≥99%), Zirconium tetrachloride (ZrCl_4_, ≥99%), Terephthalic acid (H_2_BDC, ≥99%), N, N-dimethylformamide (DMF, ≥99.5%), Ammonium acetate (≥99%), Acetic acid (≥99%), Pentane-2,4-dione (≥99%), Formaldehyde (HCHO, with methanol stable agent), Hydrogen peroxide (H_2_O_2_, 30 wt%), Hydrochloric acid (HCl, 35 wt%) were purchased from Sigma Aldrich. Nafion solution (5 wt%), Potassium ferricyanide (K_3_[Fe(CN)_6_], ≥99%), Potassium ferrocyanide (K_4_[Fe(CN)_6_], ≥99%), and Potassium chloride (KCl, ≥99%) were purchased from Aladdin. Deionized (DI) water is lab-made. All chemicals were used as received without further purification sorption.

### Preparation of U6-H

UiO-66-H (denoted as U6-H) was synthesized via a solvothermal method with minor modifications to a reported procedure^19,20^. 1.64 g of ZrCl_4_, and 1.16 g of H_2_BDC were dissolved in 80 mL DMF under continuous stirring. 0.7 mL HCl was then introduced as a modulator, and the solution was stirred until a homogeneous mixture was obtained. The precursor solution was transferred into a Teflon-lined autoclave (200 mL) and maintained at 120 °C for 24 h. After naturally cooling to room temperature, the resulting solid was collected by centrifugation and washed sequentially with DMF (three times), deionized water (two times), and acetone (once). The final product was dried at 80 °C for 12 h.

### Preparation of (U6-H)/TiO_2_

Ultrasmall sub-2 nm UiO-66-H (U6-H) nanocrystals were grown on the surface of cubic TiO_2_ via an in situ growth method. Specifically, 1.0 g of TiO_2_ was dispersed in 30 mL of DMF containing 0.1 mL of HCl solution and stirred for 10 min at room temperature. Then, 0.08 g of ZrCl_4_ was added under continuous stirring for another 0.5 h. Subsequently, 0.056 g of H_2_BDC was introduced, and the mixture was further stirred for 0.5 h. The resulting suspension was transferred into a stainless-steel autoclave lined with polytetrafluoroethylene (PTFE) and heated at 120 °C for 24 h. After cooling to room temperature, the solid MOF was collected via high-speed centrifugation and thoroughly washed three times with DMF, twice with water, and once with acetone. Finally, the obtained solid was dried in an oven at 80 °C overnight. The resulting composite, containing 5 wt.% ultrasmall U6-H nanocrystals on TiO_2_, was denoted as 5(U6-H)/TiO_2_. Samples with other U6-H loadings (2.5, 10, and 20 wt.%) were prepared by adjusting the amount of U6-H precursor and are denoted as 2.5(U6-H)/TiO_2_, 10(U6-H)/TiO_2_, and 20(U6-H)/TiO_2_, respectively. To clarify the size regime, samples in which U6-H exists exclusively as sub-2-nm nanocrystals are labelled as (U6-H)_S2_/TiO_2_, such as 5(U6-H)_S2_/TiO_2_. In contrast, samples where sub-2-nm nanocrystals coexist with larger nanoparticles are labelled as (U6-H)_S2+LP_/TiO_2_, such as 20(U6-H)_S2+LP_/TiO_2_.

### Photocatalytic tests

A commercial 200 mL batch reactor (Xi’an Taikang Biotechnology Co., Ltd) equipped with a quart window on the top was used for photocatalytic tests (Supplementary Fig. 20). A 500 W Xe lamp from Perfect (CHF-XM500) with full irradiation from 300 to 1100 nm (350 mW/cm^−2^, Newport 843-r power meter) was used as a light source. Typically, in the batch process, 5 mg catalyst was homogeneously dispersed in 100 mL DI water via sonification and then transferred into the reactor. The reactor is firstly evacuated using a vacuum pump and is filled with methane/air mixture. The total pressure was adjusted to 20 bar, followed by an equilibration period of 30 min in the dark. During the reaction, the reactor temperature was maintained at 25 ± 3 °C by an attached cooling system (Minichiller 300). After the reaction, the gas was directly injected into gas chromatography (GC) (Agilent 8860) and was analyzed by the PoraBOND Q and ShinCarbon ST 100/120 columns with Ar as the carrier gas, accompanied by a flame ionization detector (FID) and a thermal conductivity detector (TCD). The liquid products were analyzed by ^1^H nuclear magnetic resonance (NMR) spectroscopy, by mixing 0.5 mL filtered liquid sample with 0.1 mL DMSO/D_2_O solution (1/2000, v/v, DMSO is the internal standard). As CO was not detected in our system, the oxygenates selectivity was calculated based on the following Eq. (1):

1 $${{\rm{Oxygenate}}}\; {{\rm{selectivity}}}=\frac{{{\rm{n}}}\left({{{\rm{CH}}}}_{3}{{\rm{OH}}}\right)+{{\rm{n}}}\left({{{\rm{CH}}}}_{3}{{\rm{OOH}}}\right)+{{\rm{n}}}\left({{\rm{HCHO}}}\right)}{{{\rm{n}}}\left({{{\rm{CH}}}}_{3}{{\rm{OH}}}\right)+{{\rm{n}}}\left({{{\rm{CH}}}}_{3}{{\rm{OOH}}}\right)+{{\rm{n}}}\left({{\rm{HCHO}}}\right)+{{\rm{n}}}\left({{{\rm{C}}}0}_{2}\right)}\times 100\%$$

The HCHO was quantified using UV-Vis spectroscopy. The reagent solution was prepared by dissolving ammonium acetate (15 g) in deionized water, followed by the addition of acetic acid (0.3 mL) and pentane-2,4-dione (0.2 mL). The mixture was transferred into a volumetric flask and diluted to a final volume of 100 mL with deionized water. For analysis, 0.5 mL of the filtered reaction solution was mixed with 2.0 mL of deionized water and 0.5 mL of the prepared chromogenic reagent. The resulting solution was maintained at 40 °C for 1 h to allow full color development. The absorbance was subsequently recorded using UV–vis spectroscopy. The characteristic absorption at 413 nm was used for quantification, and the signal was confirmed to reach a stable value with no further increase upon repeated measurements.

### Reusability of the catalyst

Catalyst reusability was evaluated through consecutive catalytic cycles. In a typical test, the fresh catalyst (30 mg) was first exposed to the standard reaction conditions. After each run, the catalyst was recovered by filtration and dried prior to reuse. A portion of the recovered catalyst (5 mg) was then employed for a subsequent catalytic test under identical conditions, while the remaining material was used for a parallel cycling test and used for further consecutive tests.

### AQY calculation

The apparent quantum yields (AQY) at 352 nm and 418 nm were calculated according to the following Eq. (2):

2 $${{\rm{AQY}}}=\frac{{{\rm{R}}}({{\rm{electron}}})\times {{{\rm{N}}}}_{{{\rm{A}}}}}{{{{\rm{ISt}}}/{{\rm{E}}}}_{{{\rm{\lambda }}}}} \times 100\%$$

where *N*_A_, *I*, *S*, and *t* stand for the Avogadro’s constant, light irradiance on the sample (W/cm^2^), irradiation area (9.6 cm^−2^), and reaction time (3600 s), respectively. *E*_λ_ (J) is given by hc/*λ* (*λ* = 352 nm or *λ* = 418 nm). R(electron) represents the number of electrons used in the formation of the products. Since the formations of CH_3_OOH, CH_3_OH, and HCHO need 1, 3, and 5 photogenerated charge carriers, respectively, the R(electron) was calculated according to the following Eq. (3):

3 $${{\rm{R}}}({{\rm{electron}}})={{\rm{n}}}({{{\rm{CH}}}}_{3}{{\rm{OOH}}})\times 1+{{\rm{n}}}({{{\rm{CH}}}}_{3}{{\rm{OH}}})\times 1+{{\rm{n}}}({{\rm{HCHO}}})\times 5$$

### Photoelectrochemical experiments

Photoelectrochemical measurements were performed on a CS310H electrochemical workstation (CorrTest Instruments Co) using a standard three-electrode configuration. The working electrode was prepared by dispersing 5 mg of catalyst in a mixed solution containing 10 μL of 5 wt% Nafion and 2 mL of ethanol under ultrasonication. Then, 100 μL of the suspension was drop-cast onto an indium tin oxide (ITO) substrate (1 cm^2^) and dried under ambient conditions. A Pt plate and an Ag/AgCl (3 M KCl) electrode were used as the counter and reference electrodes, respectively. The electrolyte was a 0.5 M Na_2_SO_4_ aqueous solution (100 mL), with a measured pH of 6.8 ± 0.1 (*n* = 3). The photocurrent measurements were carried out under illumination of a 300 W Xe lamp. Electrochemical impedance spectroscopy (EIS) measurements were conducted using the same three-electrode configuration. The electrolyte was a deoxygenated aqueous solution containing 1 mmol L^−1^ K_3_[Fe(CN)_6_], 1 mmol L^−1^ K_4_[Fe(CN)_6_], and 0.1 mmol L^−1^ KCl (100 mL).

### Characterization

Powder X-ray diffraction (XRD) patterns were collected on a PANalytical Empyrean X-ray diffractometer operated in Bragg-Brentano configuration. Data were recorded using Cu *Kα* radiation (40 kV, 30 mA) with a step size of 0.02° and a counting time of 1 s per step. The crystalline phases were identified by comparison with reference patterns from standard databases (JCPDS).

Ultraviolet photoemission spectra (UPS) measurements were conducted using a Thermo Scientific NEXSA G2 system. The powder sample was prepared using a pellet-pressing method. The powder was evenly spread over a 1 × 1 cm conductive copper tape, covered with clean, flat aluminum foil, and pressed into a pellet using a tablet press. The aluminum foil was then removed, and the pellet was mounted onto the sample holder. The work function was determined from the secondary electron cut-off (SECO) region of the spectra. To ensure reliable energy referencing, the Fermi level position was calibrated using a sputtered and annealed Au standard^56^.

X-ray photoelectron spectroscopy (XPS) measurements were performed on a Kratos Axis Ultra DLD system equipped with a monochromatic Al Kα source (1486.7 eV). High-resolution spectra were acquired with a pass energy of 20 eV over an analysis area of approximately 300 × 700 μm. Binding energies were calibrated using the C 1 s peak with a binding energy of 284.8 eV as reference. The VBM versus NHE was calculated by combining the experimentally measured work function (*Φ*) with the *E*_f_-VBM, according to Eq. (4):

4 $${E}_{{\mathrm{VB}}},{\mathrm{NHE}}=\Phi+({E}_{{{\rm{f}}}}-{\mathrm{VBM}})-4.44$$

Surface photogenerated voltage (SPV) measurements were performed on a CEL-SPS1000 surface photovoltage spectrometer (Beijing China Education Au-Light Technology CO., LTD.), using a 500 W Xe lamp as the light source. The test wavelength range was set from 300 to 600 nm. The SPV response originates from the separation and migration of photogenerated charge carriers, which induce a measurable electric field across a capacitor-like configuration equipped with two transparent FTO electrodes.

The magnitude of the Built-in electric field (BEF) was estimated based on the relationship (5):

5 $${E=({-2{{\rm{V}}}}_{{{\rm{s}}}}\rho /{{{\rm{\varepsilon }}}{{\rm{\varepsilon }}}}_{0})}^{1/2}$$

Here, *E* represents the internal electric field magnitude, *V*_s_ corresponds to the surface voltage, *ε* denotes the low-frequency dielectric constant, and *ε*_0_ is the vacuum permittivity. According to this relationship, the internal electric field is primarily governed by the surface charge density and the corresponding surface potential^57^.

Aberration-corrected transmission electron microscopy (AC-TEM) was conducted on a JEOL 2100 F instrument operating at 200 kV. Samples were dispersed in ethanol and deposited onto holey carbon grids. High-angle annular dark-field (HAADF) images were collected with a detector spanning 73–194 mrad. Elemental distribution was probed by energy-dispersive X-ray spectroscopy using a silicon drift detector (DrySD60GV, 60 mm active area), providing a collection solid angle of about 0.6 sr.

Photoluminescence (PL) spectra were recorded on a LabRam HR spectrometer (Horiba Scientific) under excitation at 325 nm at room temperature in air. To enable reliable comparison of emission intensities, the spectra were normalized using the Raman scattering signals in the 329–332 nm region as an internal ref.^58^.

BET surface areas of the catalysts were determined by N_2_ adsorption using a Micromeritics ASAP2460-4 apparatus with liquid nitrogen at −196 °C. The samples were outgassed at 150 °C for 12 h prior to analysis.

Paramagnetic species were investigated by in situ electron paramagnetic resonance (in situ EPR) using a Bruker ELEXSYS E500 spectrometer operating in the X-band (9.5 GHz). For solid-state measurements, spectra were recorded at 100 K to suppress charge recombination, using a microwave power of 2 mW, a modulation amplitude of 1 G, a conversion time of 40 ms, and 15 scans. Spin-trapping experiments were carried out using 5,5-dimethyl-1-pyrroline N-oxide (DMPO) as the trapping agent, which was freshly prepared in water or methanol prior to use. The DMPO concentration was fixed at 80 mM. Measurements were performed under optimized conditions (microwave power: 10 mW; modulation amplitude: 0.2 G; conversion time: 5 ms; 50 scans) to ensure reliable detection of transient radical species. All in situ measurements were conducted in a sealed pressure tube to maintain a controlled atmosphere. The spectra were further analyzed using SpinFit software.

Ultraviolet-visible diffuse reflectance spectra (UV-vis) were acquired using a PerkinElmer Lambda 650 spectrophotometer over a wavelength range of 200–800 nm.

Attenuated total reflectance infrared (ATR-IR) spectra were collected on a Thermo Fisher Scientific Nicolet iS50 spectrometer equipped with a mercury cadmium telluride detector. Spectra were acquired with 32 scans at a resolution of 4 cm^−1^.

In situ Fourier transform infrared (In situ FTIR) spectra collected on a Thermo Fisher Scientific Nicolet 6700 spectrometer equipped with a mercury cadmium telluride detector^59^. Spectra were acquired with 32 scans at a resolution of 4 cm^−1^. For the measurements, the catalyst was diluted with KBr and pressed into a self-supported wafer (13 mm in diameter, containing 20 mg of catalyst). The wafer was pretreated at 150 °C under vacuum to remove adsorbed species, followed by cooling to room temperature prior to exposure to a CH_4_/Air mixture (150 mbar) and subsequent light irradiation. Pyridine adsorption FT-IR (Py-IR) experiments were conducted by introducing pyridine vapor (10 Torr) into the in situ cell, followed by evacuation to remove weakly bound species before spectral acquisition.

Femtosecond MidIR TAS (MidIR fs-TAS) measurements were carried out using the set-up described in ref. ^60^. The pump-probe measurements were performed with the pump excitation wavelength *λ*_pump_ = 350 nm, in resonance with the optical bandgap of the photocatalysts and below the direct photoexcitation of U6-H (see Fig. 2i), and the probe wavelength set to *λ*_probe_ = 4 µm (2500 cm^−1^). At this probe wavelength, only the spectral signature of the free and shallow trapped electrons is detected^32,36^. In order to compare two different samples, it is absolutely necessary to be sure that the transient signal is not depending on the intensity of the photoexcitation because the concentration of electron-hole pairs can be high enough to affect the kinetics by second order non-geminated electron-hole reactions. We carefully addressed this issue to record kinetic traces that are independent on the pump pulse intensity and pump-probe overlap (see SI for details). The MidIR TAS measurements were carried out using TiO_2_ and 5(U6-H)_S2_/TiO_2_ prepared as self-supported pellet. The catalysts were dehydrated overnight and kept under vacuum during the measurements.

## Supplementary information

Supplementary Information

Transparent Peer Review file

## Source data

Source Data

## Data Availability

The data that support the findings of the study are included in the main text and supplementary information files, or are available from the corresponding author upon request. Source data are provided with this paper.
