# Supplementary material for: Decorating titania with ultrasmall UiO-66-H crystallites enables quantitative photocatalytic oxidation of methane to oxygenates
Source: Nat Commun. 2026 Apr 29;17:5867. doi: 10.1038/s41467-026-72422-8 (PMC13334034; doi:10.1038/s41467-026-72422-8)
Supplement: Supplementary file 1 — Supplementary Information [file 41467_2026_72422_MOESM1_ESM.pdf]

Supplementary Information

Decorating Titania with Ultrasmall UiO-66-H Crystallites Enables Quantitative Photocatalytic Oxidation of Methane to Oxygenates

Geqian Fang[1], Nour Alhajjar[2], Wenjun Yu[3], Maya Marinova[4], Karima Ben Tayeb[2], Jian Lin[3], Thomas Roland[2], Pardis Simon[1], Vincent De Waele[2], Vitaly V. Ordomsky[1]*, Andrei Y. Khodakov[1]*

[1] UCCS−Unité de Catalyse et Chimie du Solide, Université de Lille, CNRS, Centrale Lille, ENSCL, Université d' Artois, UMR, 8181 Lille, France

[2] Univ. Lille, CNRS, UMR 8516 - LASIRe - Laboratoire de Spectroscopie pour les Interactions, la Réactivité et l'Environnement, F-59000 Lille, France

[3] CAS Key Laboratory of Science and Technology on Applied Catalysis, Dalian Institute of Chemical Physics, Chinese Academy of Sciences, Dalian 116023, China

[4] UMET–Institut Michel-Eugène Chevreul, Université de Lille, CNRS, INRAE, Centrale Lille, Université d'Artois, FR, 2638 Lille, France

* Corresponding authors E-mail: Andrei Khodakov (andrei.khodakov@univ-lille.fr), Vitaly V. Ordomsky (vitaly.ordomsky@univ-lille.fr)

Supplementary note

Note S1. Femtosecond UV pump – Mid-IR probe Transient Absorption Spectroscopy (TAS)

The UV pump - Mid-IR probe TAS configuration applied in this study is derived from the set-up has been described in ref[1]. Briefly, our pump-probe spectroscopy setup is based on a 4W Libra (Coherent) Ti:Sa CPA laser, delivering 110 fs pulses at the repetition rate of 1 kHz, and a central wavelength at 800 nm. The pump pulses are generated by using an OPA (OPERA model Coherent) seeded by 1.3 W at 800nm and equipped by a SFG, SHG and THG module. The Mid-IR probe pulses are generated by a second OPA (OPERA model Coherent) equipped with a DFG stage. The TAS spectra are recorded by using by a single-pass optical delay line (Microcontrole) and a 500 Hz chopper placed along the pump optical pathway. At the output of the DFG stage, low passband ZnSe and Ge neutral filters are used to filter and to attenuate the energy of the Mid-MIR probe pulses which are then split in two beams before the optical cell containing the samples. The Reference (REF) beam is steered and focused directly onto the slit of the IR spectrometer while the Signal beam (SIG) is slightly focused onto the sample and then imaged onto the IR spectrometer using gold parabolic mirrors. Both SIG and REF are dispersed and imaged using a IHR320 spectrometer (HORIBA JY) and then detected at 1kHz using a two-arrays detector (MCT, 2*32 elements; 3-13µm, InfraRed Associates). Each transient spectrum is acquired and averaged using 5000 Mid-IR probe pulses per scan. Typically, 4 to 6 scans of 130 to 300 pump-probe delays are measured.

For this study, the following specific conditions were applied:

Note S2. The Mid-IR probe pulses were centered around 4 µm (covering the IR spectral range 2400-2700 cm^{-1}), in a spectral range where only the free and shallow-trapped electrons are observed and where the U6-H and TiO_2 exhibits no vibrational bands. The TAS-IR spectra were detected with the iHR320 spectrograph equipped with a 100 lines-grating blazed at 3 µm, and the spectrum were recording in the second order of the grating. Under these conditions, and taking into account the fact that the absorption spectra of the electrons are broad and featureless, we can analyze the kinetic traces by averaging the signal over the detected spectral range. We controlled that the decays for each pixel is identical to the mean decay for each data set, as previously done in ref [2]

The pump pulses were tuned at λ_{pump} = 350 nm in resonance with the energy of the TiO_2 band gap transition. The intensity of the pump and the pump-probe overlap condition were carefully adjusted and controlled using a lens and neutral density filters. This parameter is particularly critical in the context of this study because the kinetic of the electron-hole recombination depends on the concentration of the photogenerated charge carriers and therefore it depends directly on the intensity of the pump laser pulse but also indirectly on the pump-probe overlap conditions. It is therefore critical to determine the measurements conditions for which the kinetic traces are independent on the intensity parameters. To solve this issue, we established independently for the TiO_2 and the $5(U6\text{-}H)_{S2}/TiO_2$ samples the pump-probe conditions for which the kinetic traces are not sensitive to the energy or to the beam diameter on the sample. These optimal

measurements conditions are shown in Supplementary Fig. 16 and S17 for the 5(U6-H)$_{S2}$/TiO_2 and TiO_2, respectively. The conditions of recording are given in the caption.

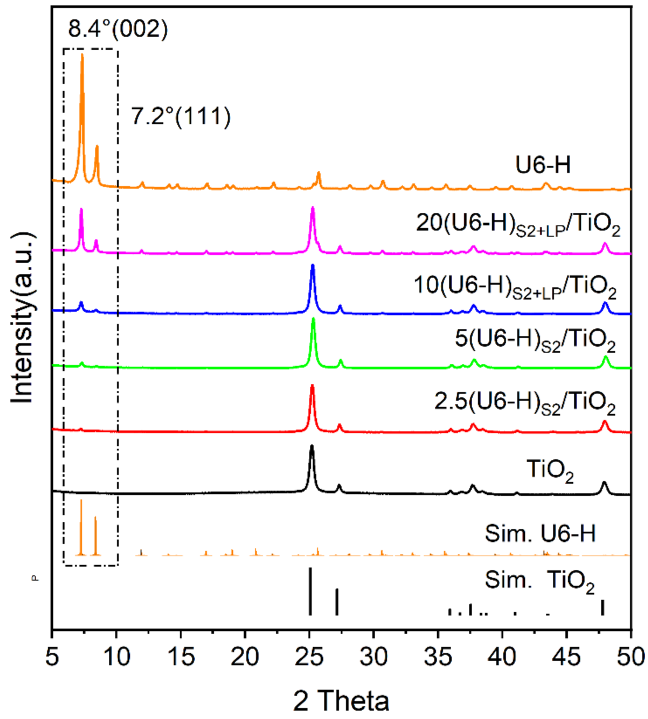

Supplementary Fig. 1. XRD pattern of U6-H, TiO_2, 2.5(U6-H)$_{S2}$/TiO_2, 5(U6-H)$_{S2}$/TiO_2, 10(U6-H)$_{S2+LP}$/TiO_2, 20(U6-H)$_{S2+LP}$/TiO_2 catalysts, together with the simulated diffraction patterns of U6-H (cm1022882_si_001.cif) and P25 TiO_2 (Anatase: JCPDS 21-1272; Rutile: JCPDS 21-1276)

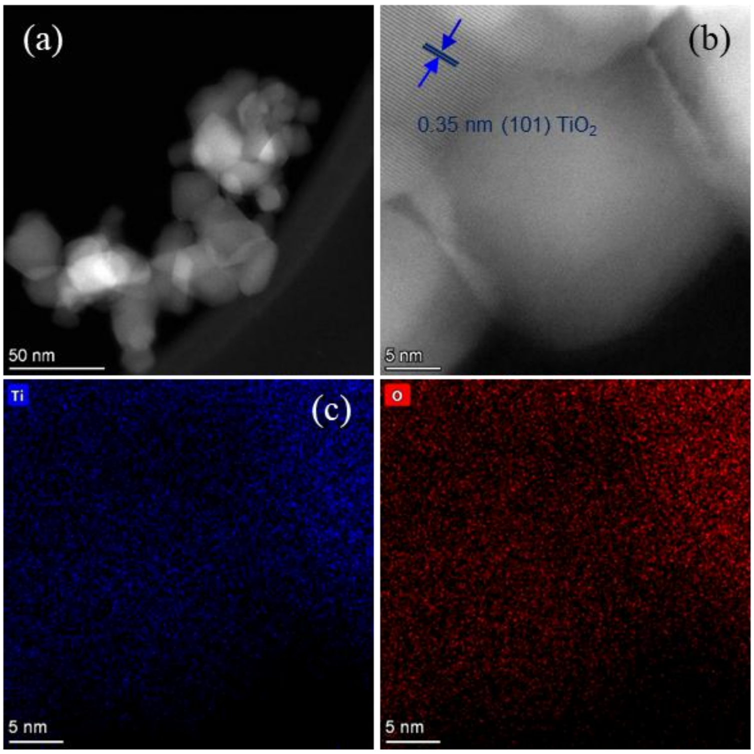

Supplementary Fig. 2. (a) AC-HADDF-TEM. (b) High-resolution-AC-HADDF-TEM, (c) EDS-Mapping of TiO_2 catalyst

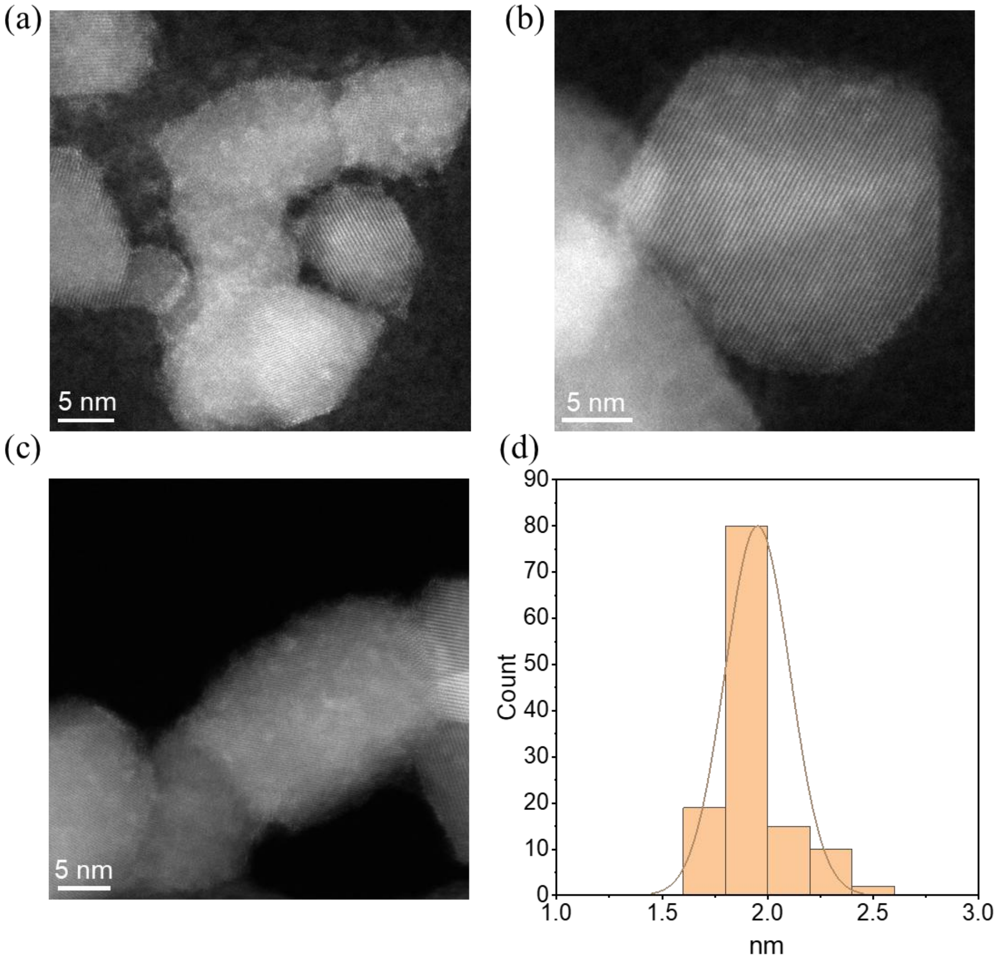

Supplementary Fig. 3 (a-c). Representative High-resolution-AC-HADDF-TEM, (d) particle size distribution of 5(U6-H)$_{S2}$/TiO_2 catalyst

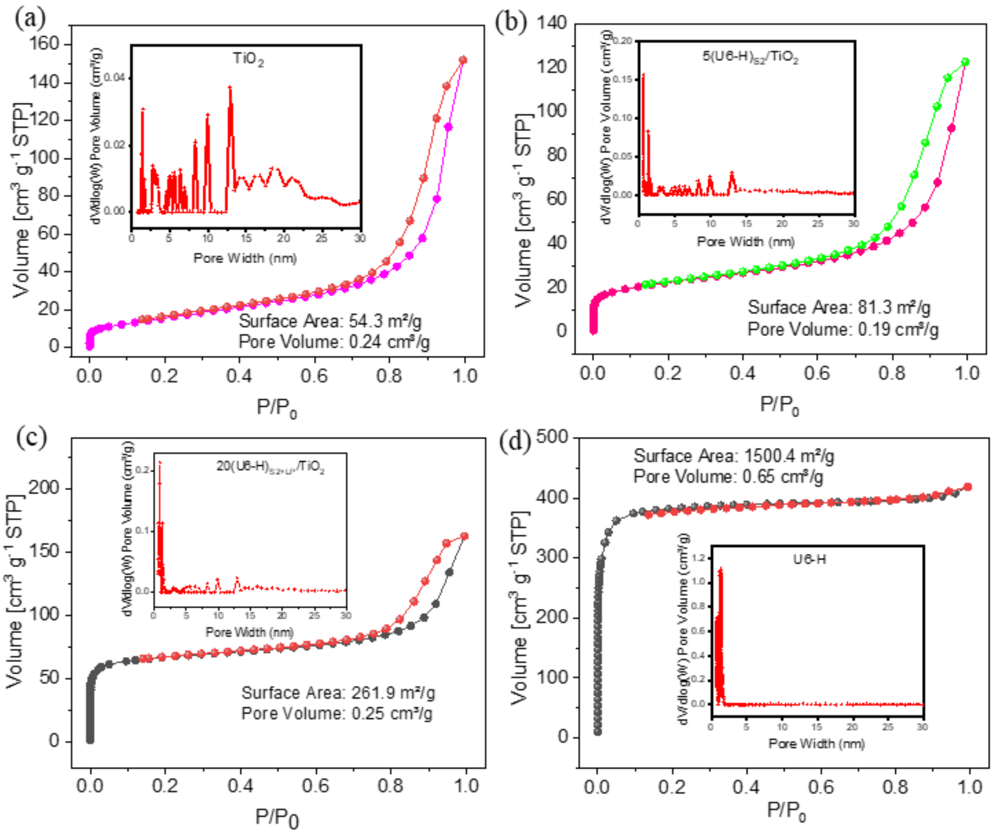

Supplementary Fig. 4. N_2 adsorption-desorption isotherms and pore-size distribution curves of (a) TiO_2, (b) $5(U6\text{-}H)_{S2}/TiO_2$, (c) $20(U6\text{-}H)_{S2+LP}/TiO_2$ and (d) U6-H catalysts

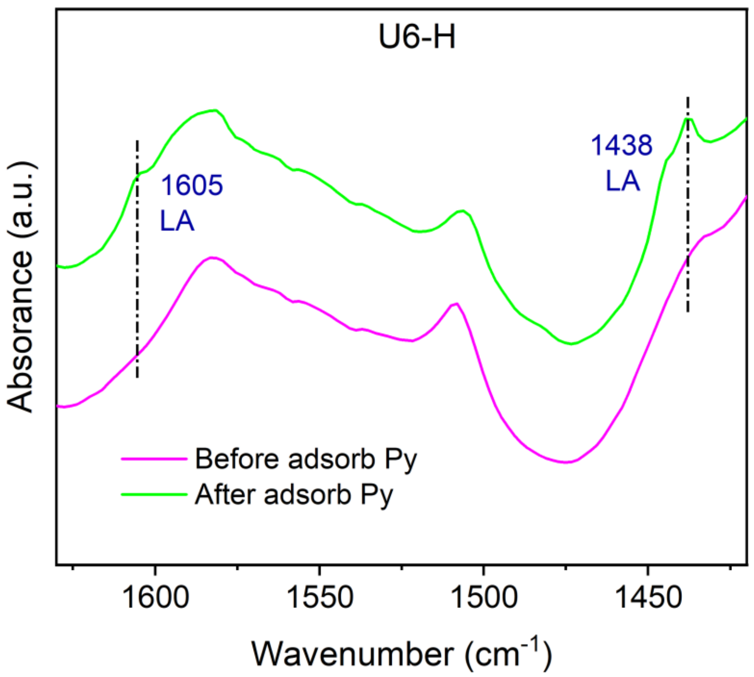

Supplementary Fig. 5. Pyridine-IR spectra on U6-H catalyst; Py: pyridine; LA: Lewis acid (dotted lines indicating Py adsorption position)

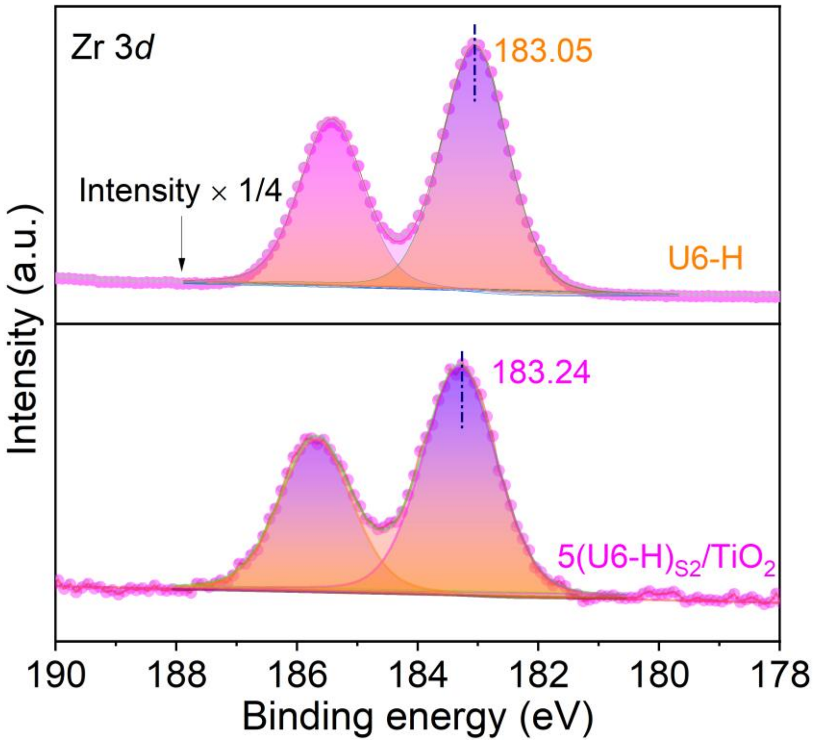

Supplementary Fig. 6. Zr 3*d* XPS spectra of U6-H and $5(U6\text{-}H)_{S2}/TiO_2$ catalysts

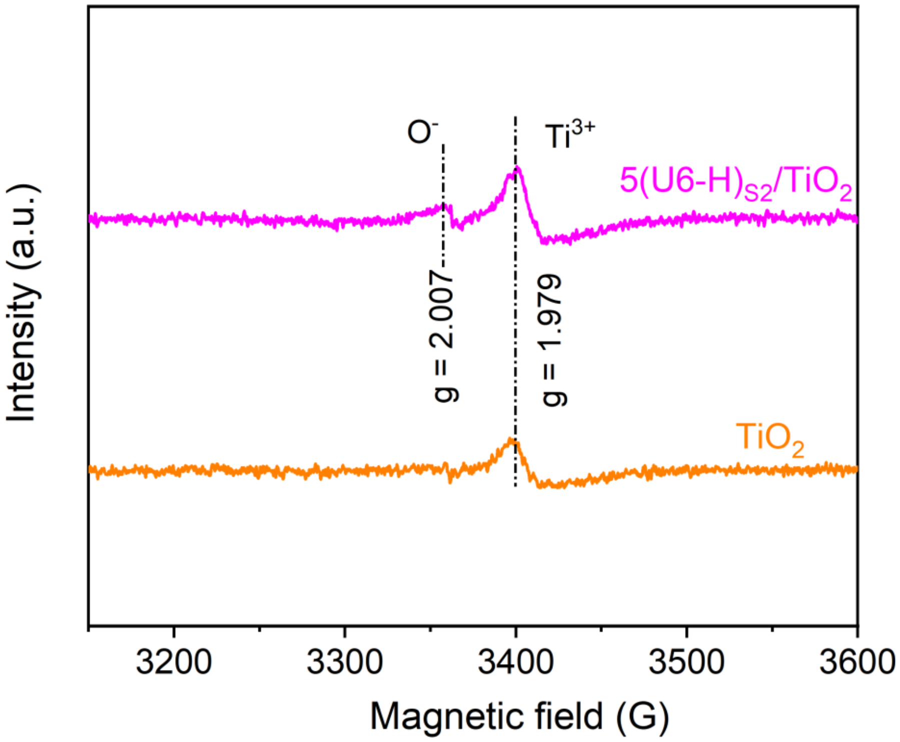

Supplementary Fig. 7. low-temperature (100 K) solid-state EPR spectra of TiO_2 and $5(U6\text{-}H)_{S2}/TiO_2$ catalysts under dark conditions

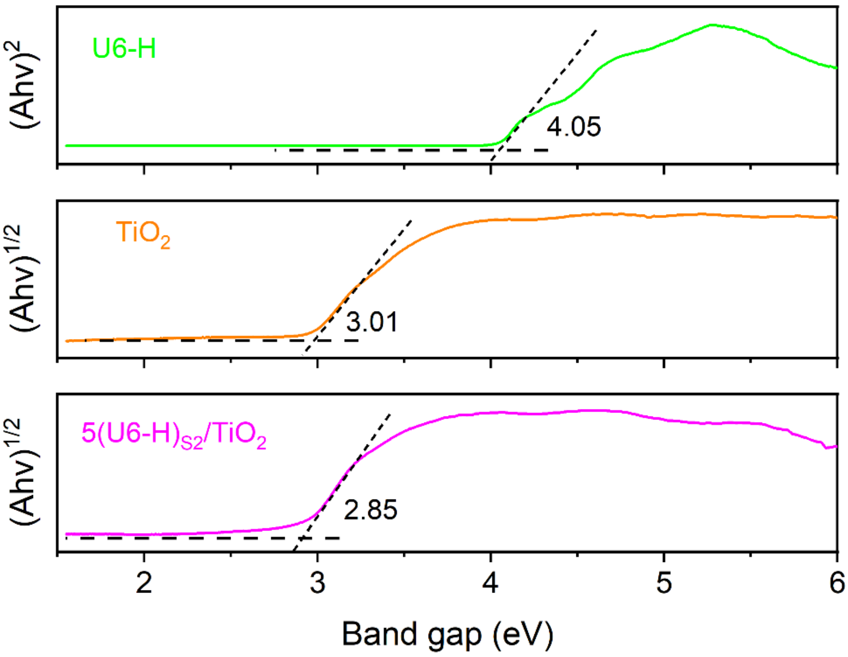

Supplementary Fig. 8. Tauc's plot derived from diffuse reflectance spectra of U6-H, TiO_2, and $5(U6\text{-}H)_{S2}/TiO_2$ catalysts

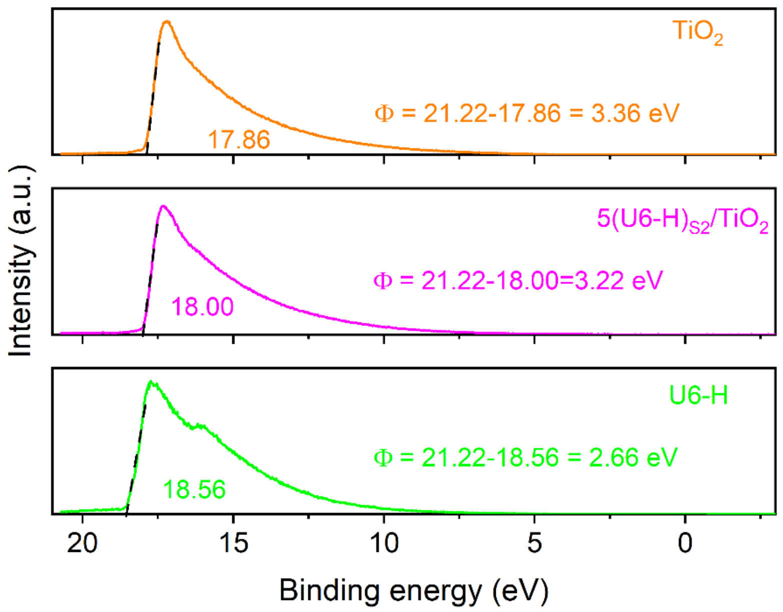

Supplementary Fig. 9. UPS spectra on U6-H, TiO_2, and $5(U6\text{-}H)_{S2}/TiO_2$ catalysts

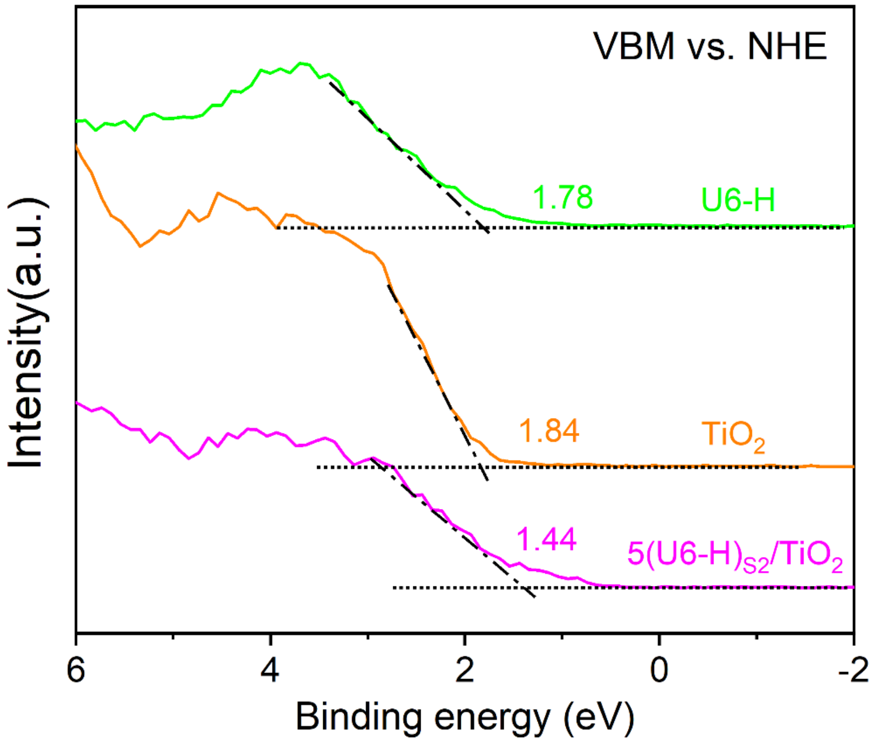

Supplementary Fig. 10. Low binding energy XPS measurements of the VBM energy region of TiO_2, U6-H, and 5(U6-H)$_{S2}$/TiO_2 catalysts. VBM versus NHE: combining the experimentally measured work function (Φ) with the VBM–E_f separation obtained from UPS, according to: E_{VB}, NHE = Φ + (E_f − VBM) − 4.44

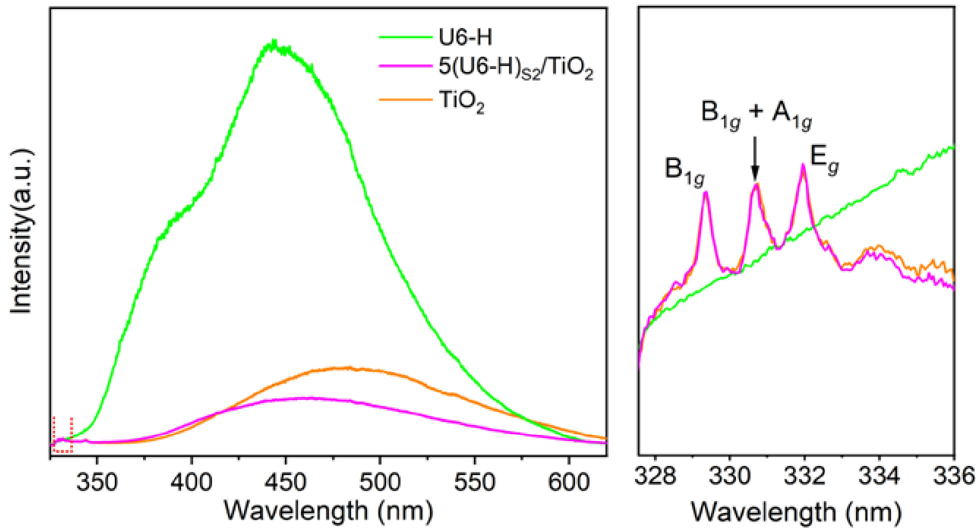

Supplementary Fig. 11. PL spectra of U6-H, TiO_2, and $5(U6\text{-}H)_{S2}/TiO_2$ catalysts with 325 nm excitation. Note that the spectra of TiO_2 and $5(U6\text{-}H)_{S2}/TiO_2$ are normalized based on the measured Raman modes indicated by the inset right image

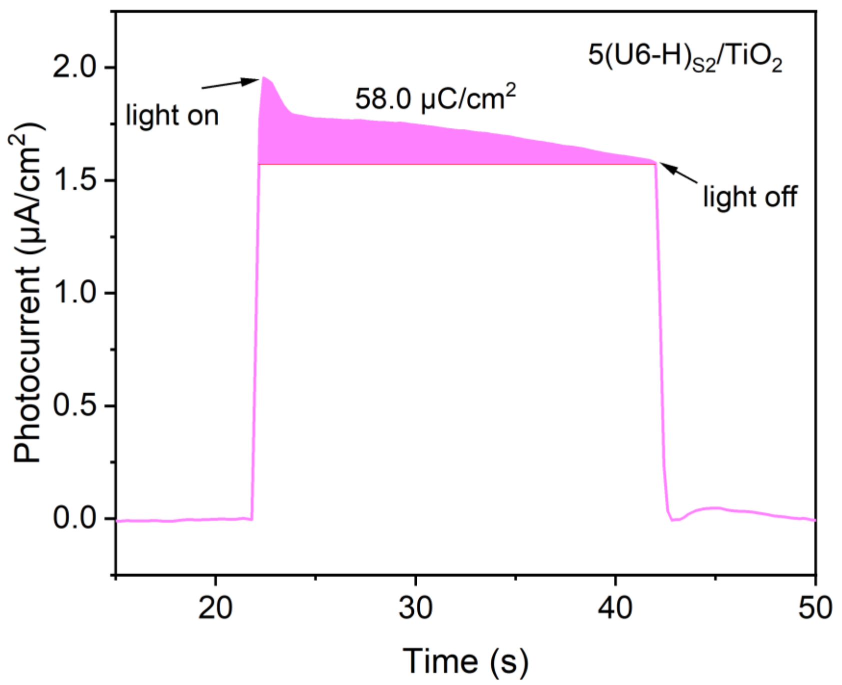

Supplementary Fig. 12. The surface charge density of 5(U6-H)$_{S2}$/TiO_2 catalyst

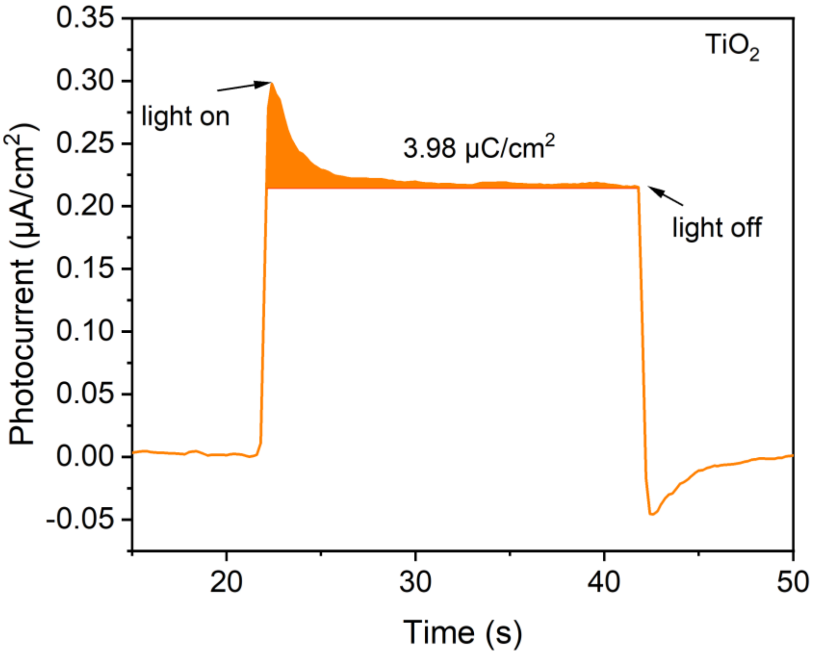

Supplementary Fig. 13. The surface charge density of TiO_2 catalyst

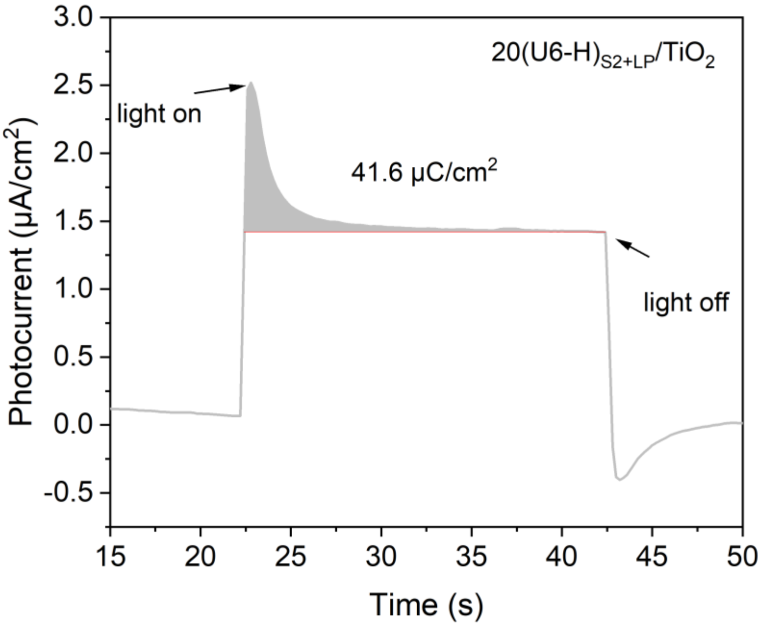

Supplementary Fig. 14. The surface charge density of $20(U6\text{-}H)_{S2+LP}/TiO_2$ catalyst

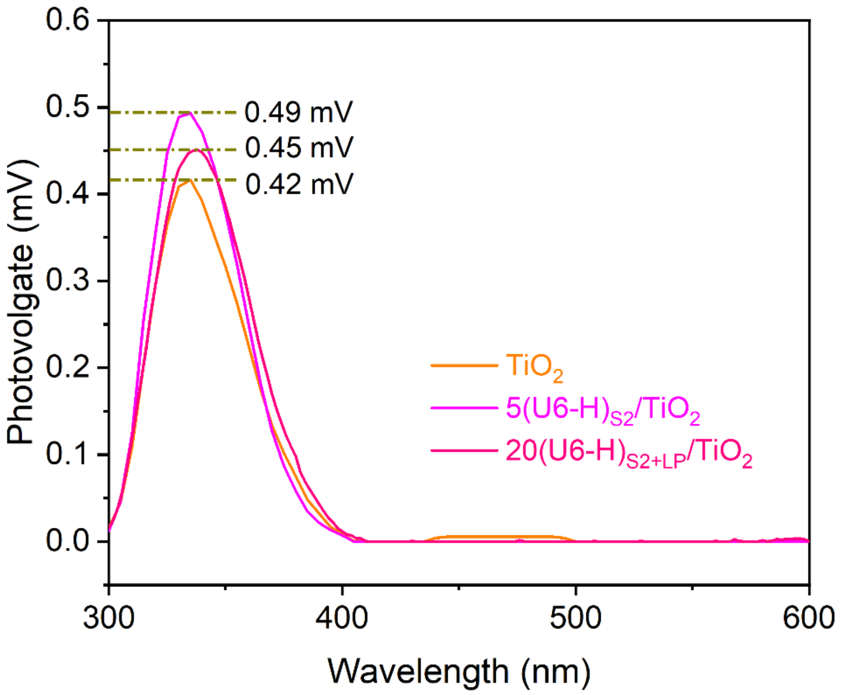

Supplementary Fig. 15. The surface photogenerated voltage of TiO_2, 5(U6-H)$_{S2}$/TiO_2, and 20(U6-H)$_{S2+LP}$/TiO_2 catalysts

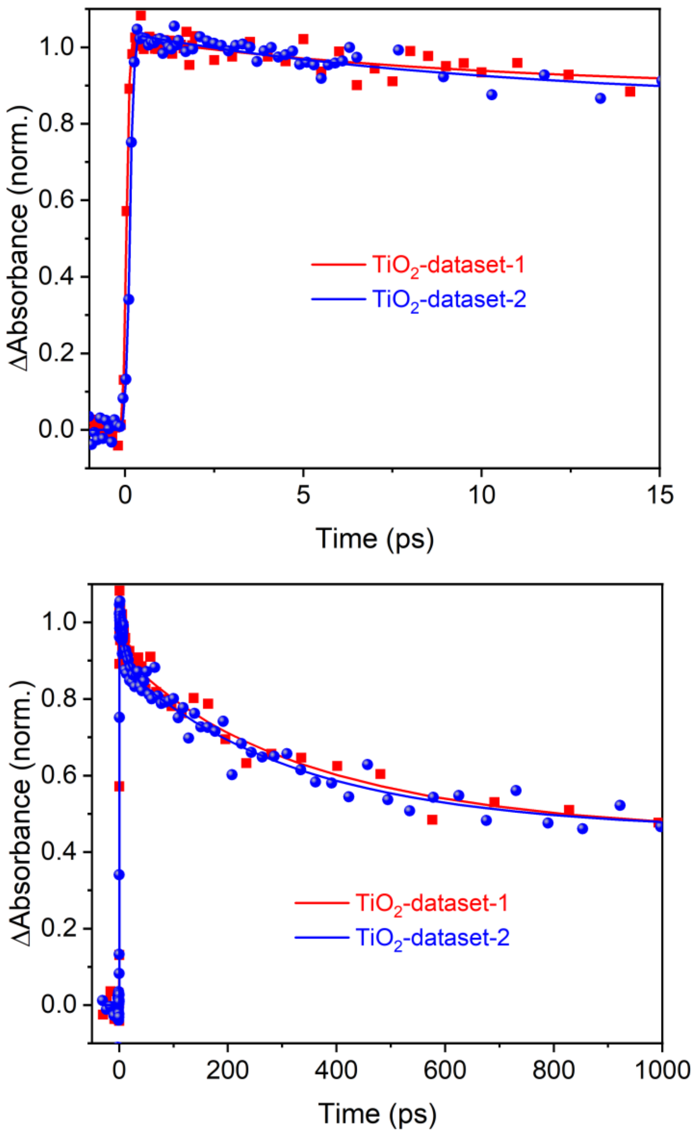

Supplementary Fig. 16. Two reference datasets recorded for TiO_2 under experimental pump-probe conditions independent on the pump laser intensity

Dataset 1 was recorded using a pump intensity of 37 nJ and an OD of 2.0. This reference decay was quasi-identical to the decay recorded with an OD of 1.6 under the strictly similar pump-probe overlap conditions. **Dataset 2** have been recorded after defocussing the pump beam and using an energy of 50 nJ (OD = 2.0).

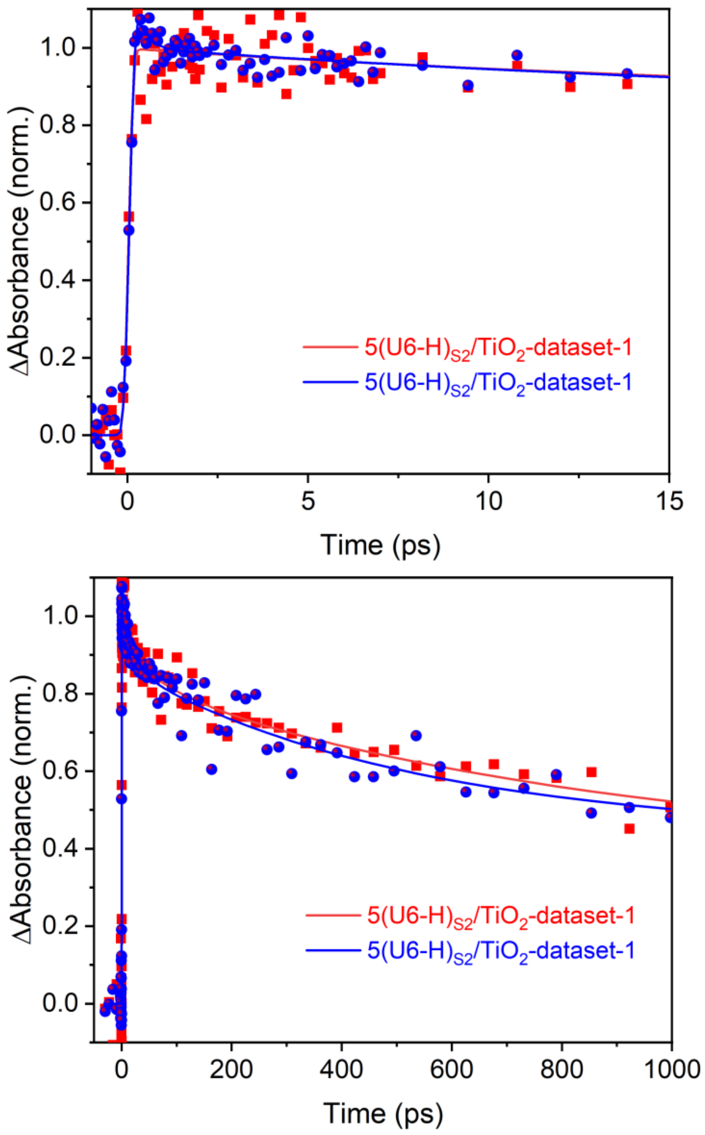

Supplementary Fig. 17. Two reference datasets recorded for 5(U6-H)$_{S2}$/TiO$_2$ under experimental pump-probe conditions independent on the pump laser intensity

Dataset 1 was recorded using a pump intensity of 37 nJ and an OD of 2.0. This reference decay is identical to the decay recorded with an OD of 2.3 under the strictly similar pump-probe overlap conditions. **Dataset 2** have been recorded after defocussing the pump beam and using an energy of 50 nJ (OD = 2.0).

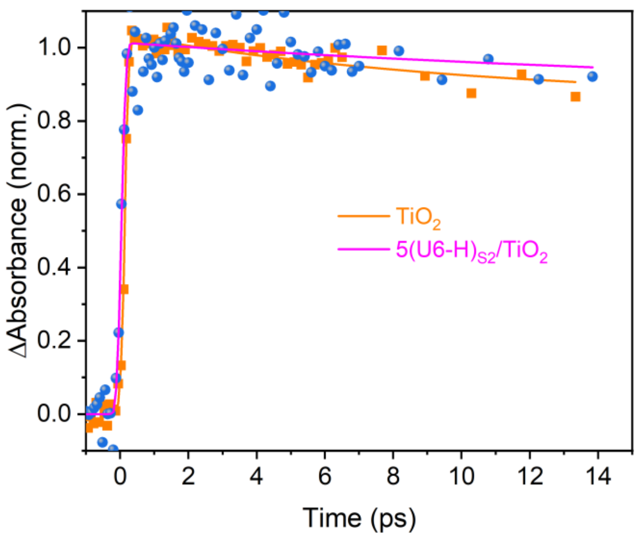

Supplementary Fig. 18. Pump-probe decay trace recorded for 5(U6-H)$_{S2}$/TiO_2 and TiO_2 between 0-15 ps (same datasets as in **Fig. 2i**)

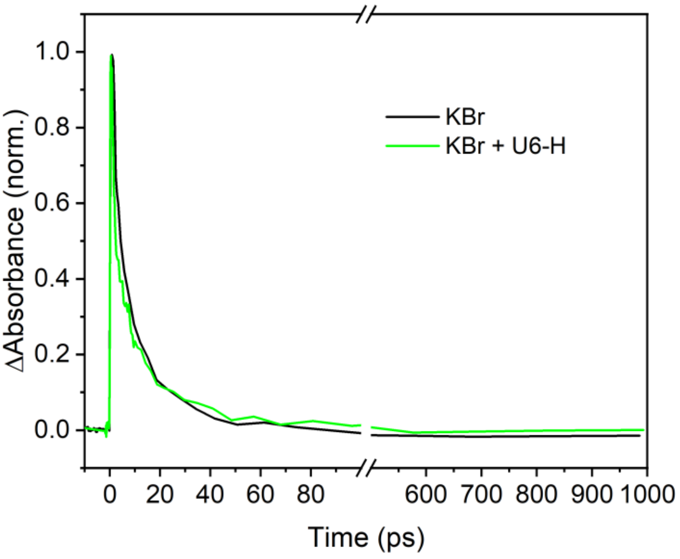

Supplementary Fig. 19. Pump–probe kinetic traces of pure KBr and of the U6-H + KBr pellet. The measurements were acquired using a 330 nm pump and a probe centered at 4750 nm, with a pump excitation energy of 80 nJ/ pulse

For a comprehensive comparison between the photodynamics of the charge carriers in the U6-H samples and in the $5(U6\text{-}H)_{S2}/TiO_2$, we first outline that U6-H absorbs only bellow 320 nm (**Fig 2d**), while the pristine absorbs strongly bellow 400 nm. We excited at 330 nm, a wavelength that is more favorable than 355 nm to excite the U6-H. For these measurements 3 mg of U6-H have been dispersed in 100 mg of KBr (≈3 wt%) and pressed as self-supported pellet. The U6-H content in the U6-H/KBr pellet is 3 times more than in the $5(U6\text{-}H)_{S2}/TiO_2$ pellet used of **Fig. 2i**. The traces clearly show that there is no decay component with a lifetime longer than a 20 ps. Therefore, we can conclude that the pump-probe signal signal recorded for the $5(U6\text{-}H)_{S2}/TiO_2$ composite (**Fig. 2i**) is the signature of the free and shallow trapped electrons from TiO_2.

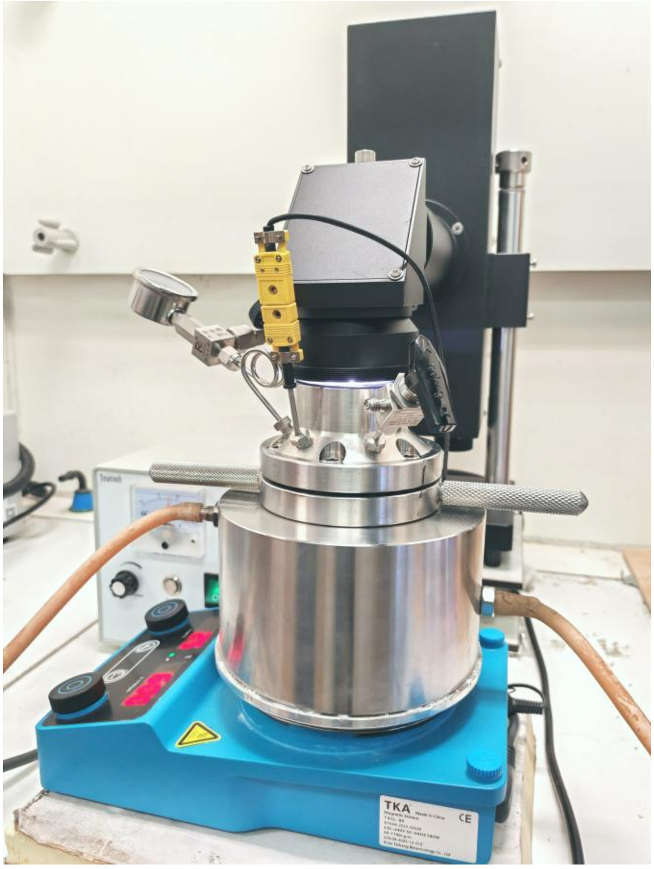

Supplementary Fig. 20. Batch photoreactor (Xi'an Taikang Biotechnology Co., Ltd) with a quart window and Xe lamp (CHF-XM500, Perfect) with full spectrum.

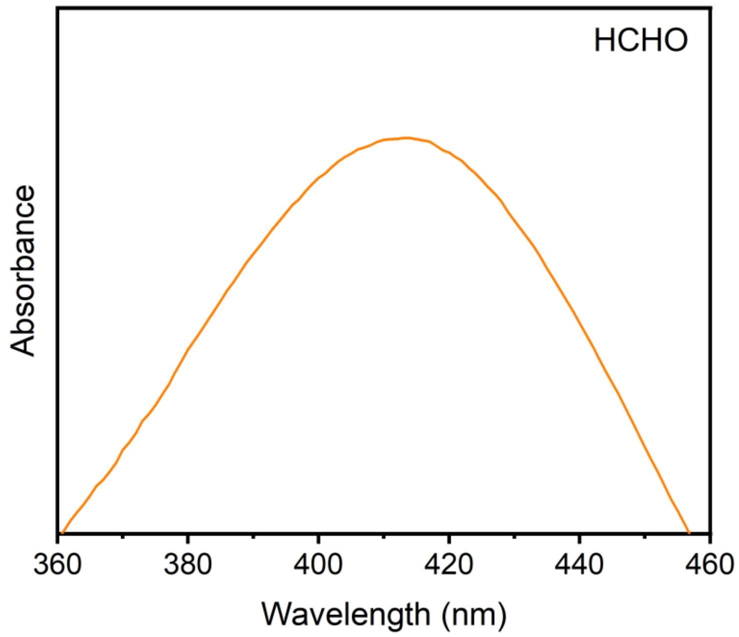

Supplementary Fig. 21. UV-Vis spectrum of HCHO products over (U6-H)$_{S2}$/TiO_2. Test conditions: Pressure (CH_4) = 18 bar, (Air) = 2 bar, Catalyst: 5 mg 5(U6-H)$_{S2}$/TiO_2, 100 mL H_2O, r.t., 500 W Xe lamp illumination for 2 h

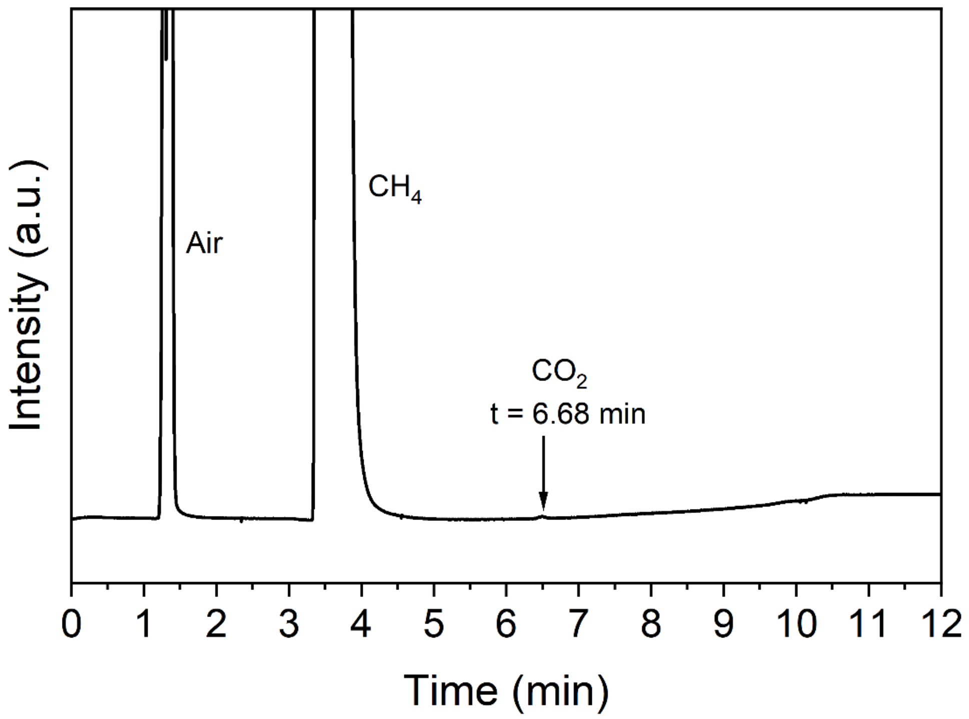

Supplementary Fig. 22. The TCD spectra of gaseous products over $(U6\text{-}H)_{S2}/TiO_2$. Test conditions: Pressure (CH_4) = 18 bar, (Air) = 2 bar, Catalyst: 5 mg $5(U6\text{-}H)_{S2}/TiO_2$, 100 mL H_2O, r.t., 500 W Xe lamp illumination for 2 h

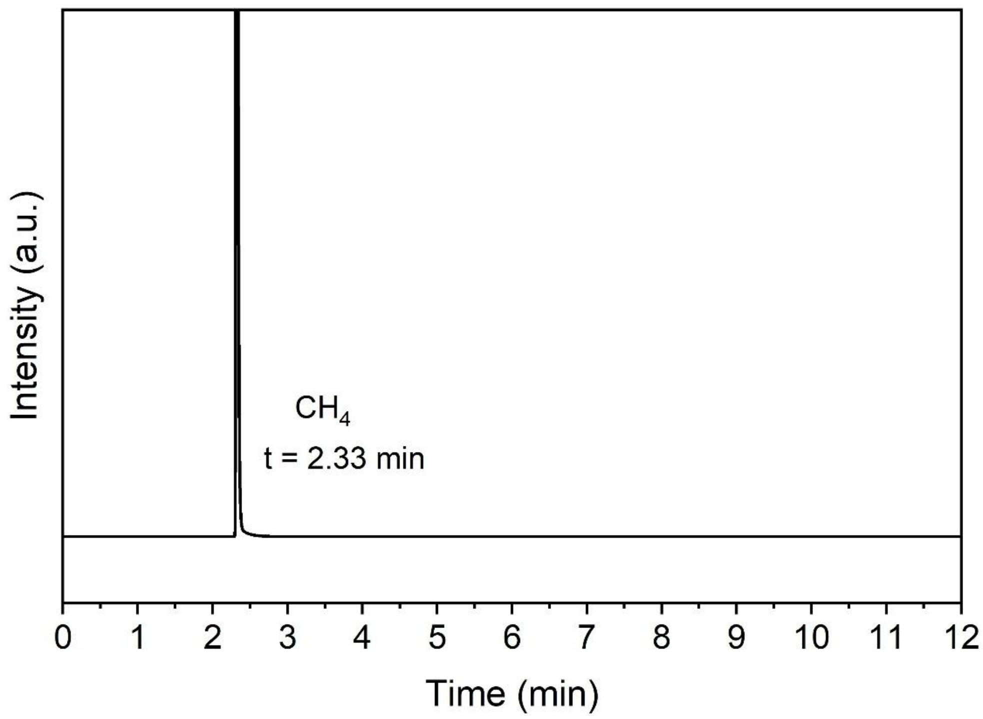

Supplementary Fig. 23. The FID spectra of gaseous products over $(U6\text{-}H)_{S2}/TiO_2$. Test conditions: Pressure (CH_4) = 18 bar, (Air) = 2 bar, Catalyst: 5 mg $5(U6\text{-}H)_{S2}/TiO_2$, 100 mL H_2O, r.t., 500 W Xe lamp illumination for 2 h

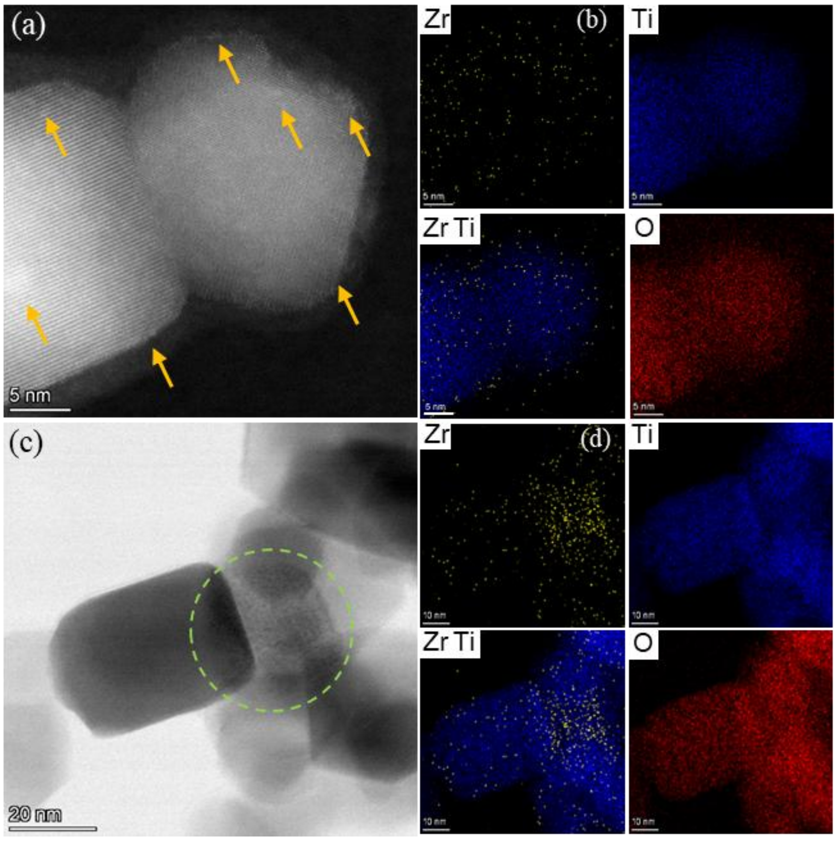

Supplementary Fig. 24 (a,c). AC-TEM and (b,d) EDS-Mapping of 20(U6-H)$_{S2+LP}$/TiO_2 catalyst (the yellow arrows and cycle pointing to MOF species)

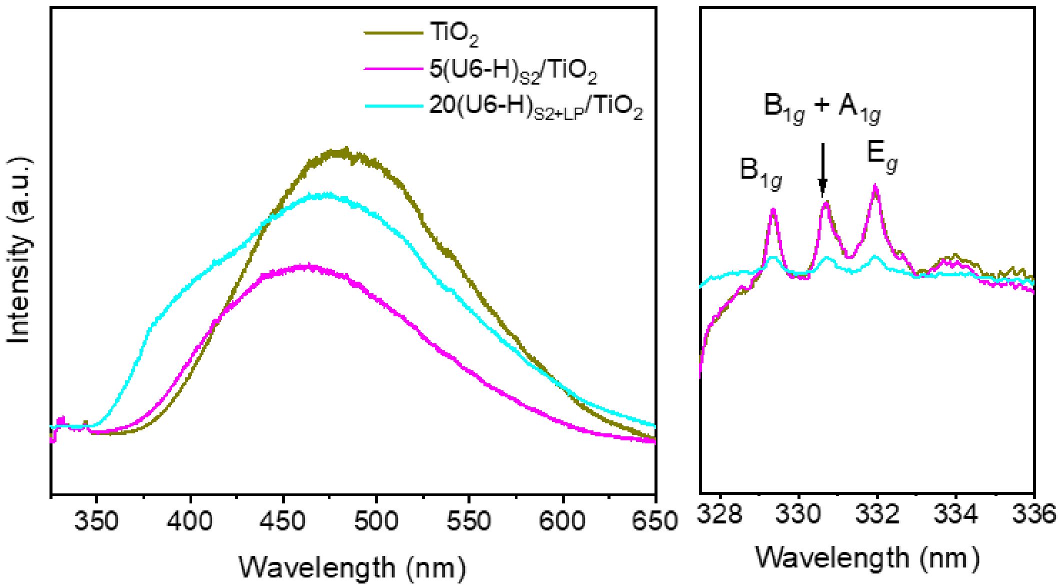

Supplementary Fig. 25. PL spectra of various TiO_2, 5(U6-H)$_{S2}$/TiO_2 and 20(U6-H)$_{S2+LP}$/TiO_2 catalysts. Note that the spectra are normalized based on the measured Raman modes indicated by the inset right image

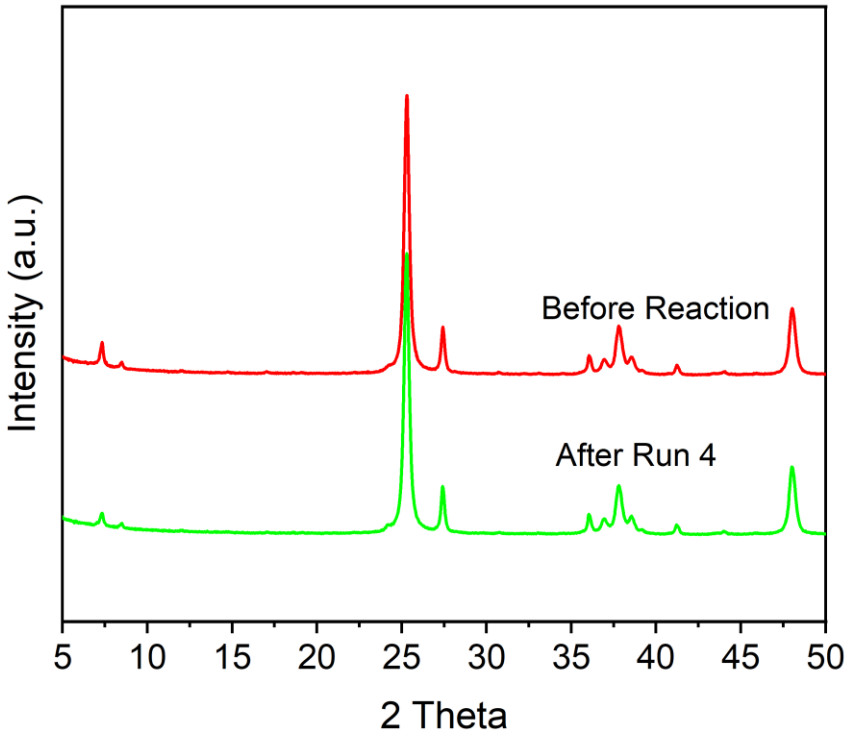

Supplementary Fig. 26. XRD pattern of 5(U6-H)$_{S2}$/TiO_2 catalysts before and after 4 cycle test

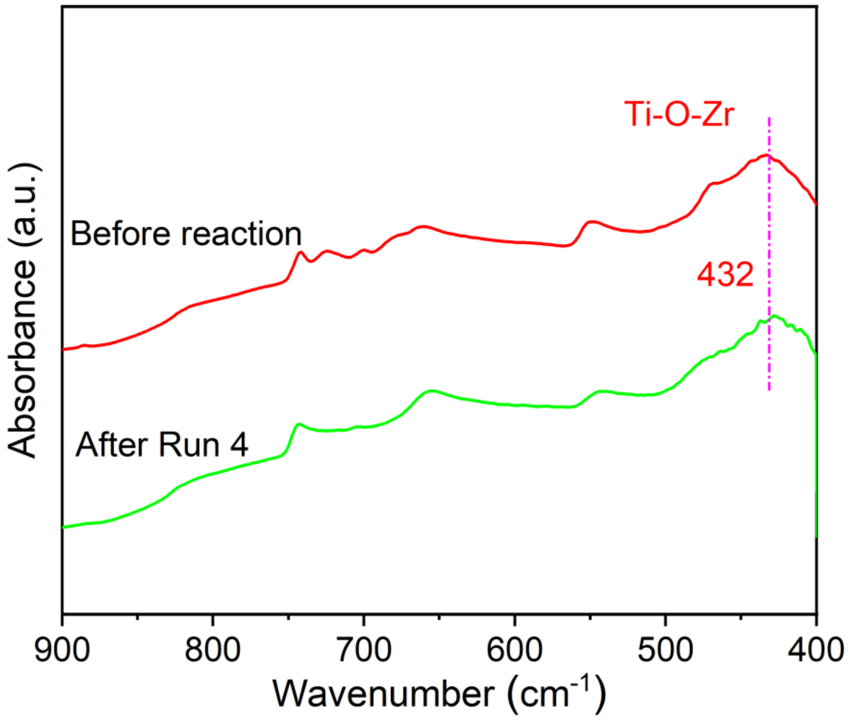

Supplementary Fig. 27. ATR-IR spectra of 5(U6-H)$_{S2}$/TiO_2 catalysts before and after 4 cycle test

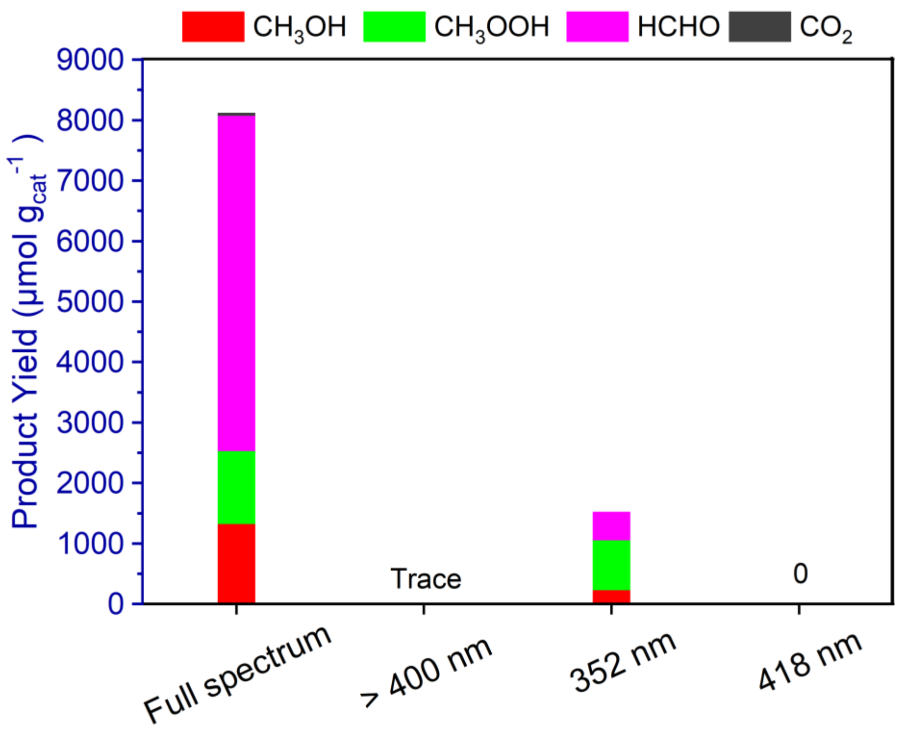

Supplementary Fig. 28. Photocatalytic CH_4 oxidation performance of 5(U6-H)$_{S2}$/TiO_2 under different wavelength. Test conditions: Pressure (CH_4) = 18 bar, (Air) = 2 bar, Catalyst: 5 mg 5(U6-H)$_{S2}$/TiO_2, 100 mL H_2O (with or without 40 μL 30 wt% H_2O_2 solution), r.t., 500 W Xe lamp (full spectrum or specific wavelength) illumination for 2 h

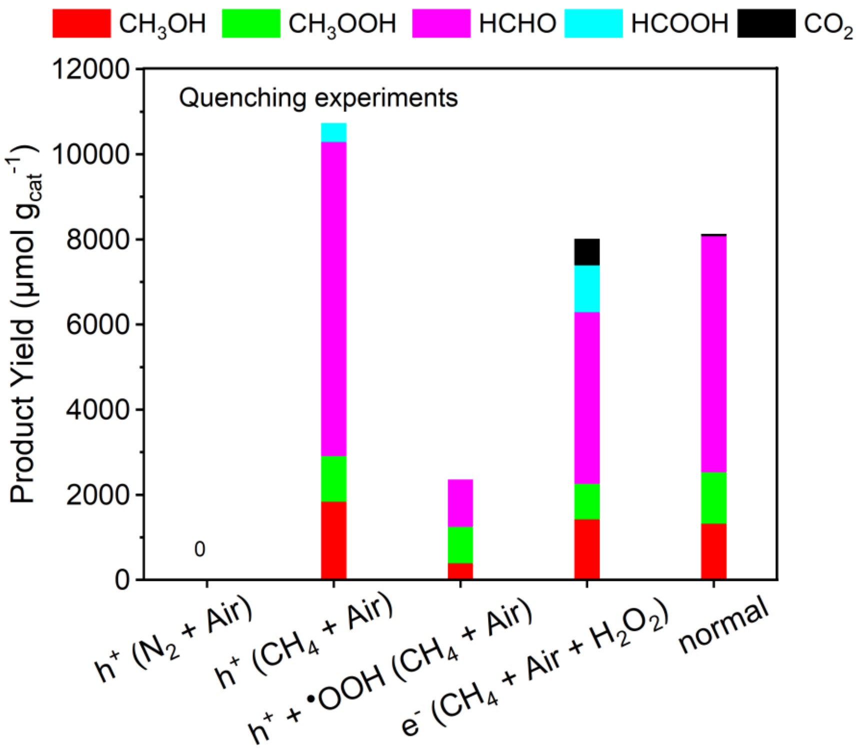

Supplementary Fig. 29. Quenching experiments on 5(U6-H)$_{S2}$/TiO_2 catalyst. The x-axis shows the quenched species and in parentheses, reactant composition. $K_2S_2O_8$, $Na_2C_2O_4$, salicylic acid, and 1,4-benzoquinone were used as quenchers for e^-, h^+, $^{\bullet}OH$, and $^{\bullet}OOH$, respectively. Test conditions: Pressure (CH_4) = 18 bar or (N_2) = 18 bar, (Air) = 2 bar, Catalyst: 5 mg 5(U6-H)$_{S2}$/TiO_2, 100 mL H_2O (with or without 40 μL 30 wt% H_2O_2 solution), r.t., 500 W Xe lamp illumination for 2 h

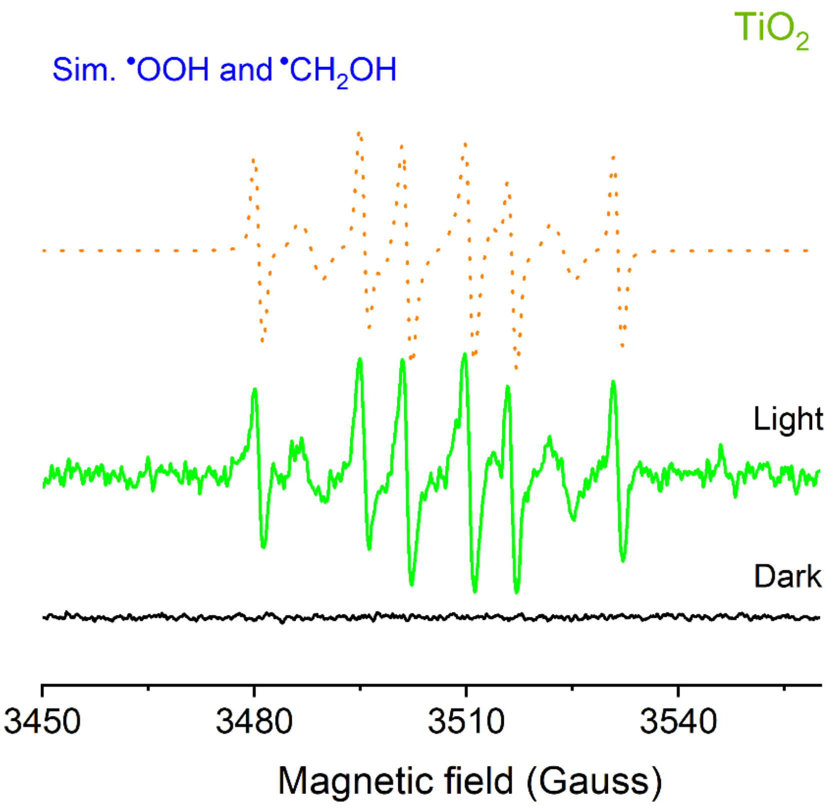

Supplementary Fig. 30. *In situ* liquid EPR of $^{\bullet}$OOH and $^{\bullet}CH_2OH$ over TiO_2 catalyst by using DMPO as spin trapping

The $^{\bullet}$OOH was detected in methanol solution (aN = 13.44 G, aH = 8.78 G, aHγ = 1.5 G) with a total spectral width of 40 G. The signal of $^{\bullet}CH_2OH$ (aN = 14.91 G, aH = 20.97 G) with a total spectral width of 52 G was also detected.

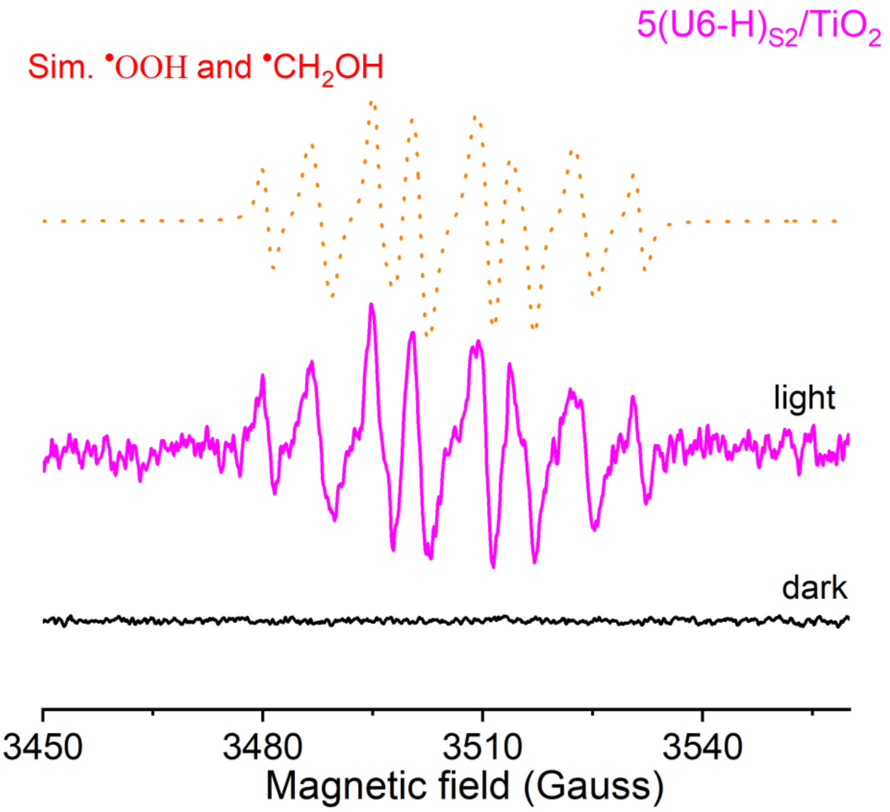

Supplementary Fig. 31. *In situ* liquid EPR of ˙OOH and ˙CH_2OH over $5(U6\text{-}H)_{S2}/TiO_2$ catalyst by using DMPO as spin trapping

The ˙OOH was detected in methanol solution (aN = 13.58 G, aH = 8.82 G, aHγ = 1.4 G) with a total spectral width of 40 G. The signal of ˙CH_2OH (aN = 14.98 G, aH = 20.84 G) with a total spectral width of 52 G was also detected.

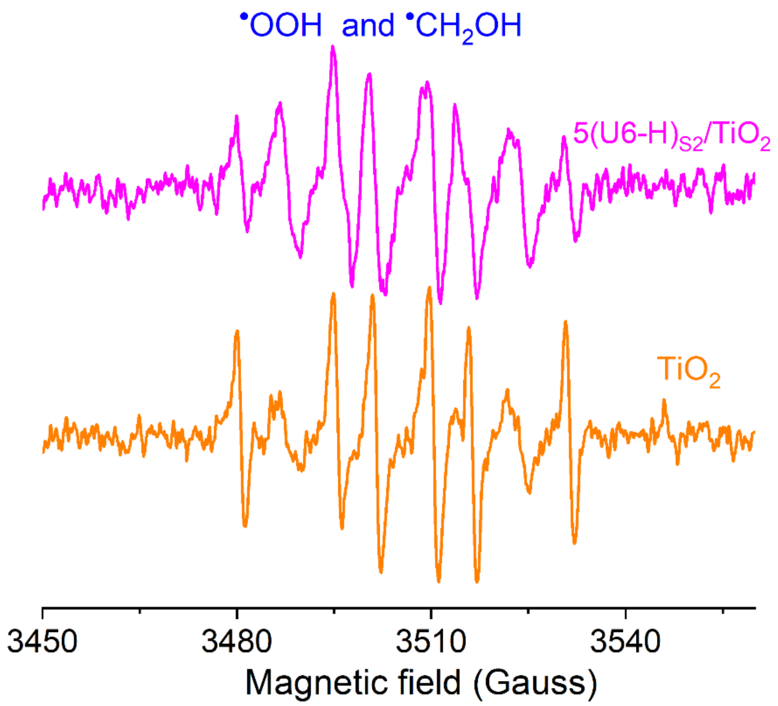

Supplementary Fig. 32. *In situ* liquid EPR of $^{\bullet}OOH$ and $^{\bullet}CH_2OH$ over TiO_2 and 5(U6-H)$_{S2}$/TiO_2 catalysts by using DMPO as spin trapping

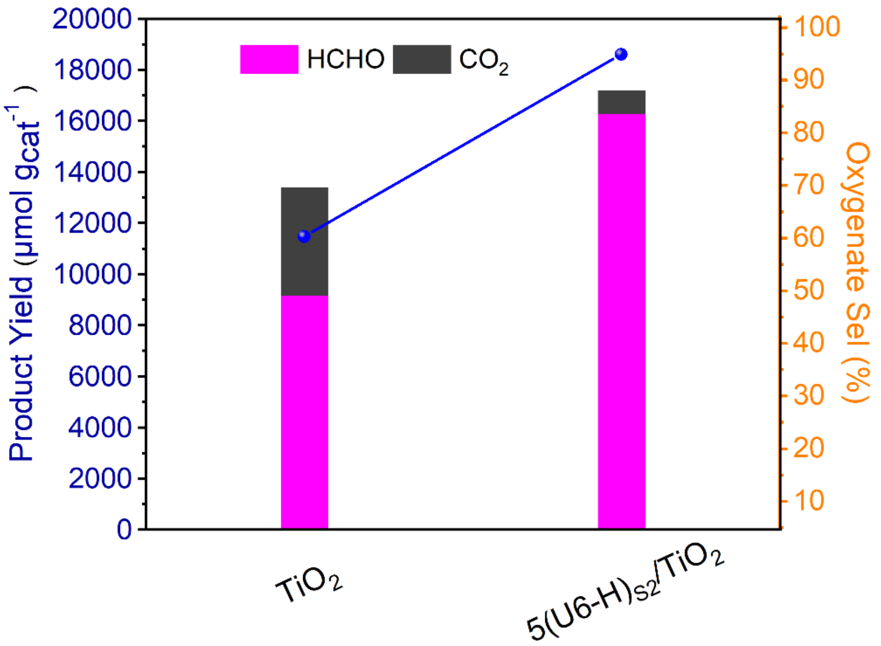

Supplementary Fig. 33. CH_3OH reactant experiments on TiO_2 and $5(U6\text{-}H)_{S2}/TiO_2$ catalysts. Test conditions: Pressure (N_2) = 18 bar, (Air) = 2 bar, Catalyst: 5 mg $5(U6\text{-}H)_{S2}/TiO_2$, 100 mL H_2O, 50 μL CH_3OH, r.t. 500 W Xe lamp illumination for 2 h

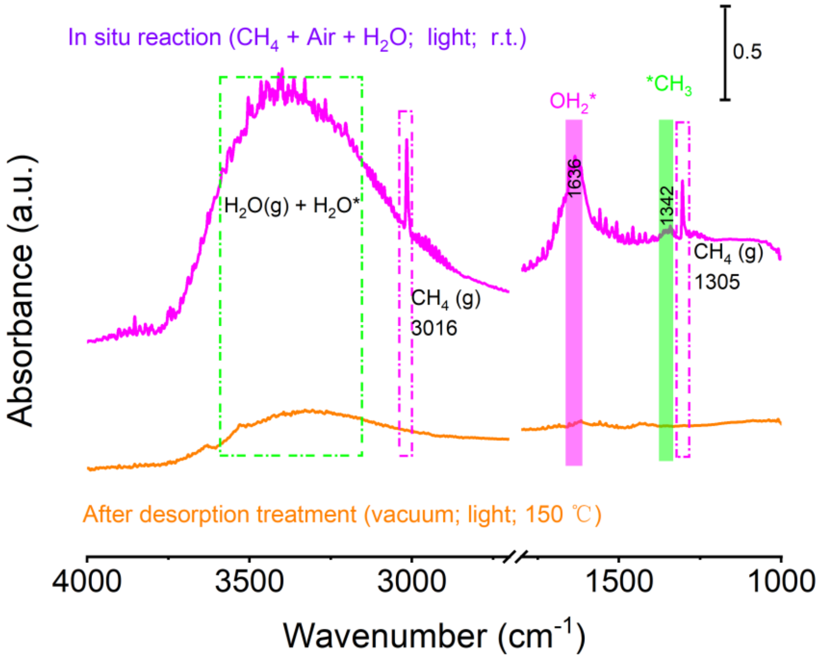

Supplementary Fig. 34. *In situ* FT-IR with desorption treatment on TiO_2 catalyst (dashed boxes and highlight area indicating adsorbed species signal).

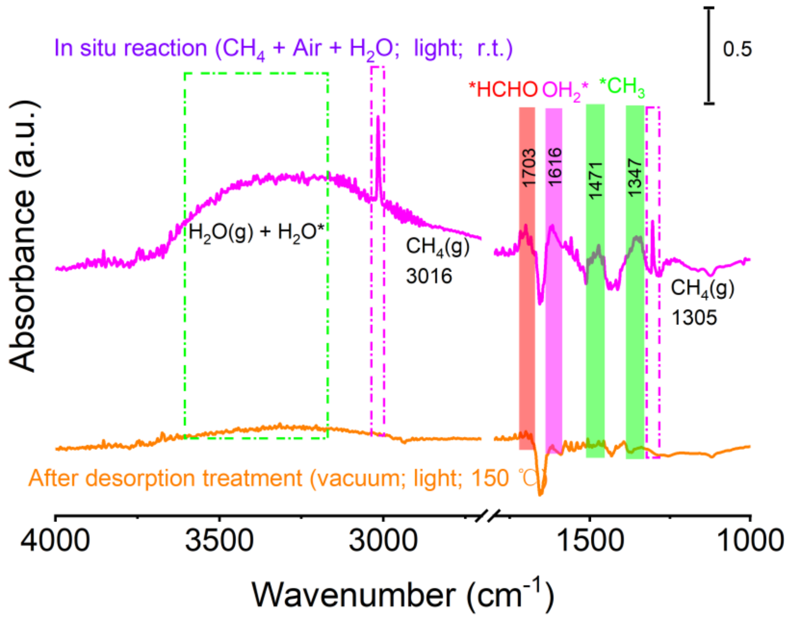

Supplementary Fig. 35. *In situ* FT-IR with desorption treatment on 5(U6-H)$_{S2}$/TiO_2 catalyst (dashed boxes and highlight area indicating adsorbed species signal).

Supplementary Table 1. Textural properties of various (U6-H)/TiO_2 catalysts

Catalyst	S_{BET}[a] (m^2 g^{-1})	V_t[b] (cm^3 g^{-1})	D_{pore}[c] (nm)
TiO_2	54.3	0.24	0.6~15
5(U6-H)$_{S2}$/TiO_2	81.3	0.19	0.6~15
20(U6-H)/TiO_2	261.9	0.25	0.6~15
U6-H	1500.4	0.65	0.6~2

[a] Brunauer-Emmett-Teller surface area; [b] Single point pore volume at P/P_0=0.99; [c] The average pore diameter (NLDFT method).

Supplementary Table 2. Measured work function and VBM of U6-H, TiO_2 and 5(U6-H)$_{S2}$/TiO_2

Catalysts	Φ[a] (eV)	E_f – VBM (eV)	VBM vs NHE[b] (eV)
TiO_2	3.36	2.92	1.78
U6-H	2.66	3.56	1.84
5(U6-H)$_{S2}$/TiO_2	3.22	2.66	1.44

[a] Measured work function (Φ), [b] EVB versus NHE = Φ + (Ef − VBM) − 4.44.

Supplementary Table 3. Performance comparison of $(U6\text{-}H)_{S2}/TiO_2$ with other catalysts in this work and previously representative catalysts for the photocatalytic CH_4 oxidation reaction

Catalysts	Reaction Conditions: Irradiance; Time(h); T(℃); Cat.(mg); P_{gas} (bar); V(mL)	Oxygenates	Oxy. Sel. (%)	Oxy. Yield $\mu mol/g_{cat}/h$	C-H Activation	Ref
0.08Pd–def–In_2O_3	420 nm LED,36.6 mW/cm^{-2}; 3; RT; 20; 19CH_4, 1O_2; 50H_2O	CH_3OH, CH_3OOH, HCHO,	82.5	5000	Radical-like	3
AC–4Co_1/PCN_{KOH}	300~780 nm Xe,170 mW/cm^2; 2, RT, 200; 8CH_4; 5H_2O	CH_3OH,	87.22	2962	Radical-like	4
q-$BiVO_4$	300~600 nm Hg, 170 mW/cm^2; 3; 25; 10; 10CH_4, 10O_2; 10H_2O	CH_3OH, HCHO	86.7	1871	Radical-like	5
Ga_2O_3	300 W Xe, 600 mW/cm^2; 2; 40; 10; 19CH_4, 1O_2; 10H_2O	CH_3OH, HCHO	87	325.4	Radical-like	6
0.54$Pd_{0.5}V_{0.2}$-TiO_2	300 W Xe, 350 mW/cm^2; 1; 25; 5;1O_2, 19CH_4, 3Ar, 2H_2/Ar; 75H_2O	CH_3OH, CH_3OOH, HCHO	89.3	5014	Radical-like	7
0.5Ni–NC/TiO_2	300~500 nm Xe; 4; 25; 10; 20CH_4, 1O_2; 180H_2O	CH_3OH, CH_3OOH, HCHO	93	4950	Radical-like	8
TiO_2(001)-C_3N_4-0.1	300 W Xe; 1; 25; 20; 0.08CH_4,0.04O_2, 1M H_2O_2; 20H_2O	CH_3OH, HCHO, HCOOH, CH_3CH_2OH,	97	696.3	Radical-like	9
$Au_{0.75}$/ZnO	300~1200 nm Xe, 100 mW/cm^2; 2; 30; 10; 15CH_4, 5O_2; 20H_2O	CH_3OH, CH_3OOH	99.1	686	Radical-like	10
$Au_{0.3}$/c-WO_3	300~780 nm Xe, 100 mW/cm^{-2}; 3; 25; 10; 19CH_4, 1O_2; 5H_2O	HCHO	~100	300	Photoholes	11
$Cu_{0.029}$/def-WO_3	420nm LED; 2; 25; 5;19CH_4, 1O_2; 120H_2O	HCHO	~100	2490	Radical-like	12
W_1/PCN-7.5	300 W Xe, 200 mW/cm^{-2}; 5; 25; 1; 5CH_4, 5H_2O	CH_3OH	~100	991.2	Radical-like	13
UION-Cu(OH)-3.48	300~1100 nm Xe; 5; 20; 5; 9CH_4, 1O_2; 20H_2O	HCHO	~100	2750	Radical-like	14
Hetero-ZnO/Fe_2O_3	300 W Xe, 100 mW/cm^{-2}; 25; 1.5; 10; 1CH_4; 5H_2O	CH_3OH, CH_3OOH	~100	119	Photoholes	15
0.2Au_1/BP	300 W Xe, 382 mW/cm^{-2}; 2; 90; 200; 30CH_4, 3O_2; 20H_2O	CH_3OH	>99	56.8	Photoholes	16
BN	300 W Xe, 660 mW/cm^2; 2; 60; 20; 1CH_4, 1O_2; 20H_2O	CH_3OH, HCHO	~100	130.2	Photoholes	17
fl-CN-530	300~780 nm Xe, 200 mW/cm^2; 3; 25; 10; 19CH_4, 1O_2; 20H_2O	CH_3OH, CH_3OOH, HCHO	99.2	790	Radical-like	18
0.5MoO_x–TiO_2	300 W Xe, 420 mW/cm^2; 2; 25; 10; 20CH_4, 1O_2; 100H_2O	CH_3OH, CH_3OOH, HCHO, HCOOH	~100	1900	Radical-like	19
5$(U6\text{-}H)_{S2}/TiO_2$	**300~1100nm Xe, 350 mW/cm^2, 0.5; 25; 5;18CH_4, 2Air; 100H_2O**	**CH_3OH, CH_3OOH, HCHO**	**~100**	**6454.3**	**Radical-like**	**This work**

Supplementary Table 4. The AQY value of $5(U6\text{-}H)_{S2}/TiO_2$ under different wavelength.

Wavelength (nm)	AQY (%)
352	6.77 ± 0.34%
418	0

The AQY was calculated to be 6.77 ± 0.34% (n = 3) under irradiation at 352 nm (6 mW cm^{-2}) with an illuminated area of 9.6 cm^2 for 3600 s, where the average amounts of CH_3OOH, CH_3OH, and HCHO were 8.23, 2.41, and 5.15 μmol, respectively.

Supplementary Table 5. The integrated area (a.u.) of the signal for $^{\bullet}OH$ in EPR spectra over TiO_2 and $5(U6\text{-}H)_{S2}/TiO_2$ catalysts

Catalysts	$^{\bullet}OH$
TiO_2	0.83
$5(U6\text{-}H)_{S2}/TiO_2$	0.46

Supplementary Table 6. The integrated area (a.u.) of the signal for $^{\bullet}OOH$ in EPR spectra over TiO_2 and $5(U6\text{-}H)_{S2}/TiO_2$ catalysts

Catalysts	$^{\bullet}OOH$
TiO_2	13
$5(U6\text{-}H)_{S2}/TiO_2$	29

Reference

1. Duplouy, L., *et al.* Ultrafast formation of exciplex species in dicyanoanthracene ZSM-5 revealed by transient emission and vibrational spectroscopy. *Eur. Phys. J-spec. Top.* **232**, 2145-2156 (2023).
2. Asbury, J.B., *et al.* Femtosecond IR Study of Excited-State Relaxation and Electron-Injection Dynamics of Ru(dcbpy)$_2$(NCS)$_2$ in Solution and on Nanocrystalline TiO_2 and Al_2O_3 Thin Films. *J. Phys. Chem. B* **103**, 3110-3119 (1999).
3. Luo, L., *et al.* Synergy of Pd atoms and oxygen vacancies on In_2O_3 for methane conversion under visible light. *Nat. Commun.* **13**, 2930 (2022).
4. Ding, J., *et al.* Asymmetrically coordinated cobalt single atom on carbon nitride for highly selective photocatalytic oxidation of CH_4 to CH_3OH. *Chem* **9**, 1017-1035 (2023).
5. Fan, Y., *et al.* Selective photocatalytic oxidation of methane by quantum-sized bismuth vanadate. *Nat. Sustain.* **4**, 509-515 (2021).
6. Han, C., *et al.* Selective Cleavage of Chemical Bonds in Targeted Intermediates for Highly Selective Photooxidation of Methane to Methanol. *J. Am. Chem. Soc.* **145**, 8609-8620 (2023).
7. Zhou, H., *et al.* Boosting Reactive Oxygen Species Formation Over Pd and VO^{δ} Co-Modified TiO_2 for Methane Oxidation into Valuable Oxygenates. *Small* **20**, 2311355 (2024).
8. Song, H., *et al.* Atomically Dispersed Nickel Anchored on a Nitrogen-Doped Carbon/TiO_2 Composite for Efficient and Selective Photocatalytic CH_4 Oxidation to Oxygenates. *Angew. Chem. Int. Ed.* **62**, e202215057 (2023).
9. Sun, X., *et al.* Molecular oxygen enhances H_2O_2 utilization for the photocatalytic conversion of methane to liquid-phase oxygenates. *Nat. Commun.* **13**, 6677 (2022).
10. Zhou, W., *et al.* Highly selective aerobic oxidation of methane to methanol over gold decorated zinc oxide via photocatalysis. *J. Mater. Chem. A* **8**, 13277-13284 (2020).
11. Wei, S., *et al.* Aerobic oxidation of methane to formaldehyde mediated by crystal-O over gold modified tungsten trioxide via photocatalysis. *Appl. Catal., B* **283**, 119661 (2021).
12. Luo, L., *et al.* Nearly 100% selective and visible-light-driven methane conversion to formaldehyde via. single-atom Cu and $W^{\delta+}$. *Nat. Commun.* **14**, 2690 (2023).
13. Wang, Y., *et al.* W Single-Atom Catalyst for CH_4 Photooxidation in Water Vapor. *Adv. Mater.* **34**, 2204448 (2022).
14. Feng, C., *et al.* Optimizing the reaction pathway of methane photo-oxidation over single copper sites. *Nat. Commun.* **15**, 9088 (2024).
15. Zheng, K., *et al.* Room-Temperature Photooxidation of CH_4 to CH_3OH with Nearly 100% Selectivity over Hetero-ZnO/Fe_2O_3 Porous Nanosheets. *J. Am. Chem. Soc.* **144**, 12357-12366 (2022).
16. Luo, L., *et al.* Water enables mild oxidation of methane to methanol on gold single-atom catalysts. *Nat. Commun.* **12**, 1218 (2021).
17. Cao, Y., *et al.* Methane Photooxidation with Nearly 100 % Selectivity Towards Oxygenates: Proton Rebound Ensures the Regeneration of Methanol. *Angew. Chem. Int. Ed.* 62, e202302196 (2023).
18. Feng, B., *et al.* Tailored Exfoliation of Polymeric Carbon Nitride for Photocatalytic H_2O_2 Production and CH_4 Valorization Mediated by O_2 Activation. *Angew. Chem. Int. Ed.* 63, e202401884 (2024).

19. Wu, P., *et al.* Subnanometric MoO_x clusters limit overoxidation during photocatalytic CH_4 conversion to oxygenates over TiO_2. *Nat. Commun.* **16**, 4207 (2025).
