# Supplementary material for: Decorating titania with ultrasmall UiO-66-H crystallites enables quantitative photocatalytic oxidation of methane to oxygenates
Source: Nat Commun. 2026 Apr 29;17:5867. doi: 10.1038/s41467-026-72422-8 (PMC13334034; doi:10.1038/s41467-026-72422-8)
Supplement: Supplementary file 2 — Transparent Peer Review file [file 41467_2026_72422_MOESM2_ESM.pdf]

nature portfolio

Peer Review File

Decorating Titania with Ultrasmall UiO-66-H Crystallites Enables Quantitative Photocatalytic Oxidation of Methane to Oxygenates

Corresponding Author: Professor Andrei Khodakov

This file contains all reviewer reports in order by version, followed by all author rebuttals in order by version.

Version 0:

Reviewer comments:

Reviewer #1

(Remarks to the Author)
This manuscript presents a study on MOF–TiO2 heterojunction catalyst for the selective photocatalytic oxidation of methane under mild conditions. The strategy of integrating sub-2 nm Zr-MOF nanocrystals with TiO2 to construct a built-in electric field facilitates charge separation and suppresses excessive •OH formation, leading to an oxygenate yield of 6454 μmol $gcat^{-1}$ h^{-1} with nearly 100% selectivity. This approach demonstrates notable novelty and potential impact.
However, several mechanistic interpretations and data presentations lack sufficient experimental evidence. Clarifying these aspects and providing more rigorous validation of the proposed mechanism are essential before the manuscript can be considered for publication.

1. In Figure 3a, the authors suggest that excessive MOF coverage leads to increased charge recombination, resulting in decreased catalytic activity; however, this claim is not adequately supported by experimental evidence.
2. Why are the 13C resonances corresponding to CH3OH and CH3OOH absent in Figure S22?
3. The manuscript mentions the apparent quantum yield (AQY) in the mechanistic discussion but does not present the corresponding experimental data. The authors are requested to provide the actual AQY values and measurement conditions to substantiate their claims.
4. In Figure 4b, the authors infer the occurrence of a photo-Fenton reaction; however, this claim lacks experimental substantiation. Please provide supporting evidence based on these cited studies or appropriate references (Appl. Catal. B: Environ. 2025, 361, 124634).
5. In Figure 4b, the authors conclude that electrons dominate the photocatalytic process; however, the role of photogenerated holes is not clearly elucidated. What function do holes play in the overall reaction?
6. According to the band structure shown in Figure 2e, both electrons and holes are transferred from the MOF to TiO2. In that case, why does the DMPO–•OH signal decrease in Figure 4d?
7. The manuscript states that CH4 is oxidized by •OH radicals; however, this claim requires supporting evidence. Please provide supporting evidence based on these cited studies or appropriate references (J. Am. Chem. Soc. 2022, 144, 740-750; Chem. Sci 2025, 16, 18652-18659).
8. The labeling of the green signal in Figure 2b appears to be incorrect.

Reviewer #2

(Remarks to the Author)
This manuscript presents a study on enhancing methane photocatalytic oxidation by decorating titania with sub-2 nm Zr-MOF nanocrystals. While the topic is of potential interest, the work suffers from significant flaws that critically undermine the validity of its key findings and conclusions. Consequently, I cannot recommend publication of this manuscript due to limited novelty and insufficient scientific rigor. The specific points of concern are detailed below.
1. The photocatalytic tests employed a 500 W Xe lamp. As indicated in Table S2 and Figure 3b, this light intensity is substantially higher than those typically used in reported literature for methane oxidation (300 W). The observed enhancement in activity is more likely an artifact of the increased photon flux rather than an intrinsic property of the catalyst's structure. Therefore, the performance comparisons made in Table S2 and Figure 3b are invalid and do not meaningfully

demonstrate the superiority of the developed catalyst.
2. The authors utilized a broad-spectrum light source (300-100 nm). However, the UV-Vis spectra (Figure 2d) confirmed that both TiO_2 and 5(U6-H)S2/TiO_2 primarily absorbed only in the UV region. The contribution of Vis and IR portions of the spectrum to the reported activity remains unaddressed. Since most literature in this field employed UV light, the apparent performance improvement in this work could potentially stem from thermal or other effects induced by Vis/IR irradiation, rather than from the proposed structural modifications of the catalyst.
3. The TEM images provided (Fig. 1b-c, S2b) lack the clarity and resolution necessary to conclusively identify and confirm the presence of Zr-MOF nanocrystals with sizes below 2 nm. To substantiate this critical claim, the authors must provide a representative particle size distribution derived from multiple TEM images, statistically validating the nanocrystal size.
4. The XRD patterns are presented without reference to standard diffraction cards. This omission makes it impossible for the reader to verify the crystal phases.
5. To properly compare the Lewis acid sites, Pyridine-IR spectra for the pristine U6-H should be included.
6. The UV-Vis spectra (Figure 2d) appear to show enhanced absorption in the UV region for pure TiO_2, not for the 5(U6-H)S2/TiO_2. This observation contradicts the narrative of enhanced light harvesting due to the composite structure.
7. A significant discrepancy exists between the UV-Vis, Band gap, PL spectra and the EIS, PC spectra. The former set shows minimal differences between TiO_2 and 5(U6-H)S2/TiO_2, while the latter indicates pronounced changes. The authors must provide a plausible explanation for this inconsistency.
8. For a comprehensive comparison of charge carrier behavior, the pump-probe decay curves for the U6-H sample should be provided.

Reviewer #3

(Remarks to the Author)
Review of NCOMMS-25-80571
Recommendation = acceptance after minor revisions

Summary
In this paper the authors present the work titled "In-situ Decorating Titania with Sub-2 nm Zr-MOF Nanocrystals for Quantitative Methane Photocatalytic Oxidation”. In this work they produced a heterojunction photocatalyst, where ultrasmall sub-2 nm Zr MOF ((U6-H)S2) nanocrystals were grown in situ on the surface of P25 TiO_2 particles. These photocatalysts were capable of oxidising CH_4 in air with high selectively to CH_3OH, CH_3COOH and CH_2O (with little over oxidation to CO_2). The show that this catalyst achieves state of the art performance in terms of product selectivity (i.e. near 100%) and activity (i.e. 6454 μmol $gcat^{-1}$ h^{-1}). The materials are well characterized by a whole suite of methods, including the use of various scavengers (and other conditions) to verify the activity of their catalysts.

The work is of high quality, and I would be happy to accept this after minor revisions, with my primary point of disagreement being their interpretation of XPS and UPS data, which can be used to construct a band energy diagram. My analysis indicates that a Type II alignment will form, where hole carriers will be encourage to migrate from the TiO_2 layer into the MOF, which is detailed in Q12 below.

Typos
T1) “as cocatalysts/promoters for important semiconductor, such TiO_2” should be “as cocatalysts/promoters for important semiconductors, such TiO_2”
T2) “using air as oxidant.” Should be “using air as an oxidant.”
T3) Fig. S1 should state what is the meaning of 2.5, 5, 10 and 20 in the composite samples.
T4) The caption of Figure 1 requires some revision. Image d) is incorrectly labelled as EDS mapping (this should be image e), which is not mentioned in the caption). And the arrows drawn in figures c) and d) should be described (which I can see are pointing to MOF sites, however, not all MOF sites are pointed to, so the authors should make clear that only some MOF sites are point to as examples).
T5) The caption of Figure S3 says that the N_2 sorption data is of “various (U6-H)/TiO_2 catalysts” however, this is rather of a) TiO_2, b) 5(U6-H)s2/ TiO_2, c) 20(U6-H) and d) TiO_2. This error is present in various figure captions. Please revise this error throughout to be more precise in your captions of what is being displayed.
T6) In Figure 2, the image (d) says “wavelgenth” but rather should say “wavelength”. Also, (b) says “wavenumbers” but convention is to write “wavenumber”. Also, in the caption you write, “UV-vis spectra”, please be more specific, as I believe that these are “diffuse reflectance UV-vis spectra”. You then write, “photocurrent spectra”. These are not spectra, as you did not investigate a range of discrete wavelengths. Rather, this is simply some chronoamperometry measurement of the photocurrent density with light on/off cycles. Please state the applied potential and light source used. And you later write, “(g) the BEF intensity for on TiO_2..” which should rather be “(h) the BEF intensity for TiO_2…”
T7) “To further unveil the charge migration and transfer efficiency in composite” should be “To further unveil the charge migration and transfer efficiency in the composite”
T8) Figure S11 has misspelt wavelength
T9) “reaching 8158.5 μmol gcat-1 within 2 h”, the -1 is not all superscript as it should be.
T10) In Figure 4, the labels “wavenumbers” should instead be “wavenumber”

Specific Queries
Q1) In the introduction, the authors use the term, “delivering promoter-level”. I request they add some information on what they mean by this term.
Q2) Some clarification should be given from the outset of the results and discussion section as to the series of MOF/ TiO_2 samples produced, and what is the meaning of the S2 subscript in some samples (I presume due to the MOF being sub-2nm

in size), whereas in others it is not present (again, I presume due to the MOF being larger than sub-2nm in size).
Q3) The authors state that "both 5(U6-H)S2/TiO2 and TiO2 exhibit a cubic morphology". I would re-phrase this statement. There are a variety of shapes seen, which is typical of P25.
Q4) The label on Figure S6 should not be "band gap spectra" but rather, a Tauc plot of diffuse reflectance data. Also, the fact that you have plotted the square of (Ahv) means that you are determining the direct allowed transition. In P25, the majority phase is anatase, where the lowest energy transition is an indirect allowed transition, and therefore, one should plot the square root of (Ahv).
Q5) Similar to above, Figure S7 does not display "VBM spectra", but rather, low binding energy XPS measurements of the VBM energy region of your catalysts. Please revise.
Q6) In the caption of Figure S8, please state the excitation wavelength of light.
Q7) The authors write, "Compared to pristine U6-H, the 5(U6-H)S2/TiO2 composite exhibits enhanced absorption in the UV region, with a slightly extended absorption range than TiO2. This enhancement can be attributed to the interfacial interaction between TiO2 and ultrasmall U6-H nanocrystal. The band gap energies (Eg) were calculated using Tauc' plot (Fig. S6)… etc."
In my view, the band edge of TiO2 before and after the addition of the MOF changes only marginally, and likely, within the error of the measurement. And regarding the bandgap values determined from your Tauc plots (which should be redone using the correct square root rather than the square), these values are also within error, given the uncertainty in where to extrapolate each band edge to the energy axis. Therefore, please tone down your statements regarding bandgap reductions.
Q8) The authors write, "the valence band maximum (VBM) of (U6-H)S2/TiO2 measured by XPS is 2.66 eV vs. NHE, which is significantly lower than that of TiO2 (2.92 eV, vs. NHE) and U6-H (3.56 eV, vs. NHE)".
In my view, these values are not vs the NHE scale. They were measured in an XPS instrument under vacuum, and likely, these values are therefore vs the Fermi level (EF) of each material. As such, please modify Figure 2(e) accordingly, where the y-axis should be changed to "Potential vs the EF of each material". What this graph and XPS shows us, in my view, is a reduction in energy between the VBM and EF upon addition of the MOF to TiO2, indicating a flow of donor density from TiO2 into the MOF.
You later write, "This observation is consistent with the result that, after the introduction of sub-2 nm U6-H, the VBM of (U6-H)S2/TiO2 shifts to a lower level compared to TiO2 (2.56 vs. 2.92 eV, Fig. 2e), thereby weakening its ability to oxidize H2O into •OH". Which based on the above, I feel should be deleted (as one has not determined their VBM with respect to the NHE, one cannot make the above statement".
Q9) I would remove your surface charge density analysis from the work. You write, "Besides, as displayed in Figs. S9 and S10, the surface charge density of 5(U6-H)S2/TiO2 was calculated to be higher than that of TiO2 (58.0 vs 3.98 μC cm−2)". Now looking at the data, if it were surface charge, you would also see discharging in the dark. For the composite, you do not see that, however, for the TiO2 sample, you do. Therefore, there is more photo-induced capacitance (i.e. surface charging) in the TiO2 sample than in the composite.
Q10) In Figure 2h), how the BEF intensity was calculated should be made more clear to the reader
Q11) The caption of Figure S23 should make clear what scavenger was used in each experiment.
Q12) You write, "The presence of the Type II liked band structure between TiO2 and junctions interface prevents effectively the recombination of electron−hole pairs and boost the photocatalytic activity" From the diagram in Figure 5a, you have drawn Type I straddling gap heterojunction, not a Type II.

I have now looked into your data in more detail. You have measured the work functions of your materials, in Figure S30. In my view, this work function represents the energy gap from the Fermi level of your material to vacuum, and therefore, one can create a correct band energy diagram using this data and the data in Figure S7. For your TiO2 sample, the work function is 3.36 eV vs vacuum, and therefore, on the NHE scale = 3.36 - 4.44 = -1.08 V vs NHE. And for your MOF sample, the work function is 2.66 eV vs vacuum, and therefore, on the NHE scale = 2.66 - 4.44 = -1.78 V vs NHE. Now from the XPS data in Figure S7, we know that the VBM should be 2.92 eV below the EF for TiO2, putting this at 1.84 V vs NHE and 3.56 eV below the EF for the MOF, putting this at 1.78 V vs NHE.

EF
(V vs NHE) VBM
(V vs NHE)
TiO2 -1.08 1.84
U6-H -1.78 1.78

Using this information, one can now construct a band energy diagram, which is indeed a Type II architecture, where hole carriers flow into the MOF from TiO2.

Please consider this analysis in your revised version.

Reviewer #4

(Remarks to the Author)
In this manuscript, the authors constructed a (U6-H)S2/TiO2 interface with a robust built-in electric field (BEF) by in situ growth of sub-2 nm ultrasmall MOF nanocrystals on the TiO2 surface for the selective photocatalytic oxidation of methane to oxyhenates (HCHO, CH3OH, and CH3OOH)。 Although the catalyst exhibits relatively superior performance, certain noteworthy issues remain unexplained in the manuscript. Therefore, I recommend publishing this paper in Nature Communications after the following revisions.

1. It is essential to explain the reasons behind the formation of sub-2nm ultra-small MOFs.
2. Please provide direct evidence demonstrating the formation of Ti-O-Zr chemical bonds.
3. Figure 4a indicates that oxygenated products are formed even in the absence of air. It is therefore necessary to clarify the origin of oxygen in the oxidation products.
4. The author claimed that "after introduction of highly dispersed sub-2 nm MOF nanocrystals onto the TiO2 surface via an in-situ growth method, the oxygenate yield increased markedly, while CO2 formation was effectively suppressed". Please explain the reasons for this phenomenon.
5. The author attributes the reduction in CO_2 to highly dispersed sub-2 nm MOF crystals on the surface of TiO2. However, no CO_2 was observed in the products of two control samples lacking sub-2 nm MOF crystals. Please explain the reason.
6. To better illustrate the effect of sub-nanostructures on the built-in electric field strength, it is necessary to add the built-in electric field strengths of 10(U6-H)/TiO_2 or 20(U6-H)/TiO_2 in Figure 2h.
7. The duration of each cycle in Figure 3d requires clarification.
8. The interfaces play important roles and more relevant works are suggested to be referred such as 10.1016/j.apcatb.2024.124001 and 10.1016/j.apcatb.2025.125411.

Version 1:

Reviewer comments:

Reviewer #1

(Remarks to the Author)
I have carefully reviewed the authors' revised manuscript and their detailed point-by-point responses. The authors have made a conscientious effort to address all of my previous comments, including additional evidence for charge carrier separation, supplementary 13CH4 experiments, the inclusion of AQY data, and expanded quenching studies. These revisions substantially improve both the scientific rigor and the overall completeness of the manuscript.
Nevertheless, several points still require further clarification before the manuscript can be considered for acceptance. I believe that the manuscript will be suitable for publication once the authors adequately address the following issues:
1. In Fig R6(a), the authors propose that the addition of the electron scavenger significantly suppresses radical generation and H2O2 formation, thereby inhibiting the Fenton process. This interpretation would be further strengthened if the authors could demonstrate whether adding H2O2 together with the electron scavenger restores the activity.
2. According to the energy structure diagram in Fig. 2e, the introduction of the MOF appears to weaken both the reduction ability of electrons and the oxidation ability of holes. The authors should clarify why the ·OOH radical concentration increases while the ·OH radical concentration decreases under these conditions.
3. The energy structure diagram in the revised manuscript differs notably from that in the previous version. The authors should briefly explain the origin of this difference.

Reviewer #2

(Remarks to the Author)
The authors have carefully revised the manuscript. The revised manuscript can be considered for publication.

Reviewer #3

(Remarks to the Author)
Reviewer 3
I am grateful for your detailed response to my comments and am satisfied with the changes that you have made to the manuscript. I am therefore happy to recommend this work for publication in this journal.

Reviewer #4

(Remarks to the Author)
The authors have responded well to all the comments, and I recommend accepting this manuscript.

Version 2:

Reviewer comments:

Reviewer #1

(Remarks to the Author)
The manuscript is now recommended for acceptance.

Open Access This Peer Review File is licensed under a Creative Commons Attribution 4.0 International License, which permits use, sharing, adaptation, distribution and reproduction in any medium or format, as long as you give appropriate credit to the original author(s) and the source, provide a link to the Creative Commons license, and indicate if changes were made.
In cases where reviewers are anonymous, credit should be given to 'Anonymous Referee' and the source.
The images or other third party material in this Peer Review File are included in the article's Creative Commons license, unless indicated otherwise in a credit line to the material. If material is not included in the article's Creative Commons license and your intended use is not permitted by statutory regulation or exceeds the permitted use, you will need to obtain permission directly from the copyright holder.
To view a copy of this license, visit https://creativecommons.org/licenses/by/4.0/

Response to reviewers

GENERAL: We are grateful to the reviewers for all their comments. We have considered all of them carefully and have accordingly revised the manuscript. The changes are highlighted. The detailed responses to all the critical points are listed below, with the original comments in italics.

Reviewer 1: *This manuscript presents a study on MOF–TiO2 heterojunction catalyst for the selective photocatalytic oxidation of methane under mild conditions. The strategy of integrating sub-2 nm Zr-MOF nanocrystals with TiO2 to construct a built-in electric field facilitates charge separation and suppresses excessive •OH formation, leading to an oxygenate yield of 6454 μmol gcat*$^{-1}$ *h*$^{-1}$ *with nearly 100% selectivity. This approach demonstrates notable novelty and potential impact. However, several mechanistic interpretations and data presentations lack sufficient experimental evidence. Clarifying these aspects and providing more rigorous validation of the proposed mechanism are essential before the manuscript can be considered for publication.*

Question 1. *In Figure 3a, the authors suggest that excessive MOF coverage leads to increased charge recombination, resulting in decreased catalytic activity; however, this claim is not adequately supported by experimental evidence.*

Reply: Thank you for the comment. As shown in **Fig. R1**, a certain amount of oxygenates is produced on TiO_2, but with a poor selectivity (55%). After introduction of highly dispersed sub-2 nm MOF nanocrystals onto the TiO_2 surface at loadings below 5 wt% via an *in-situ* growth method, the oxygenate yield increased markedly, while CO_2 formation was effectively suppressed. However, further increase in the U6-H content to 10 wt% and 20 wt% leads to a decline in the oxygenate yield.

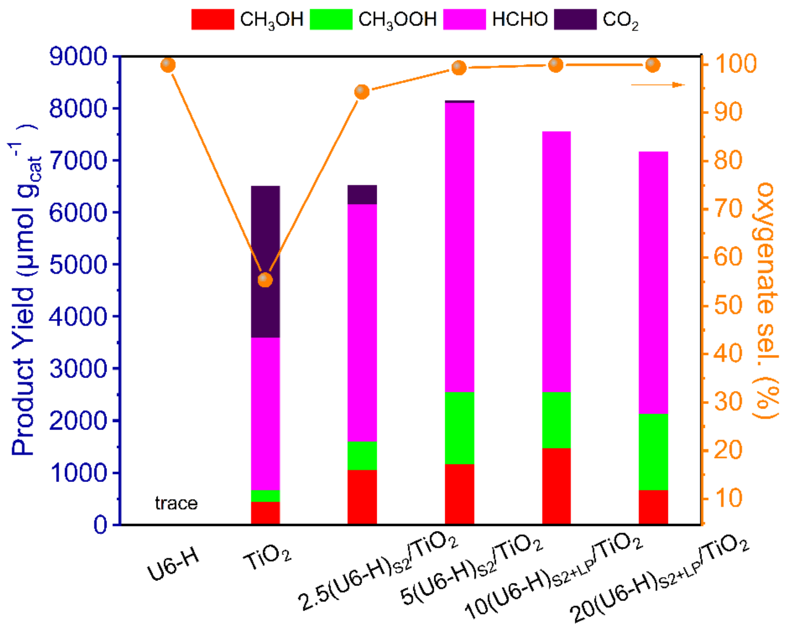

Fig. R1 Yield and selectivity of oxygenates in the photocatalytic methane oxidation over various (U6-H)/TiO_2 catalysts.

As shown in **Fig. R2a, b**, AC-HADDF-TEM and EDS-Mapping analyses show (U6-H)$_{S2}$ species on the 20(U6-H)$_{S2+LP}$/TiO_2 sample. However, the higher precursor concentrations also lead to the formation of larger MOF particles on the TiO_2 surface, which should decrease the effect of MOF on TiO_2 (**Fig. R2c, d**). We further measured the PL spectra of TiO_2, 5(U6-H)$_{S2}$/TiO_2 and 20(U6-H)$_{S2+LP}$/TiO_2 catalysts. As shown in **Fig. R3**, 20(U6-H)$_{S2+LP}$/TiO_2 exhibits a higher PL intensity than 5(U6-H)$_{S2}$/TiO_2, but still lower than that of pure TiO_2. These results demonstrate that the 20(U6-H)$_{S2+LP}$/TiO_2 sample suffers from increased charge recombination, leading to a lower catalytic activity compared to 5(U6-H)$_{S2}$/TiO_2. This trend is fully consistent with the observed photocatalytic performance order: 5(U6-H)$_{S2}$/TiO_2 > 20(U6-H)$_{S2+LP}$/TiO_2 > TiO_2.

Accordingly, we added the PL spectra as **Fig. S25** in the revised supporting information (Page 29) and the corresponding discussion in the revised manuscript (Page 15, middle).

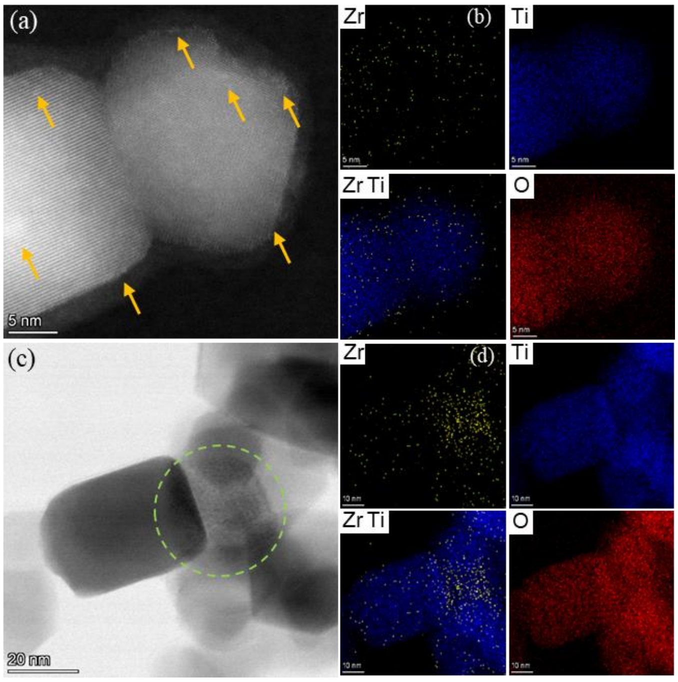

Fig. R2 (a, c) AC-TEM and (b, d) EDS-Mapping of 20(U6-H)$_{S2+LP}$/TiO_2 catalyst (yellow arrows and cycle pointing to MOF species)

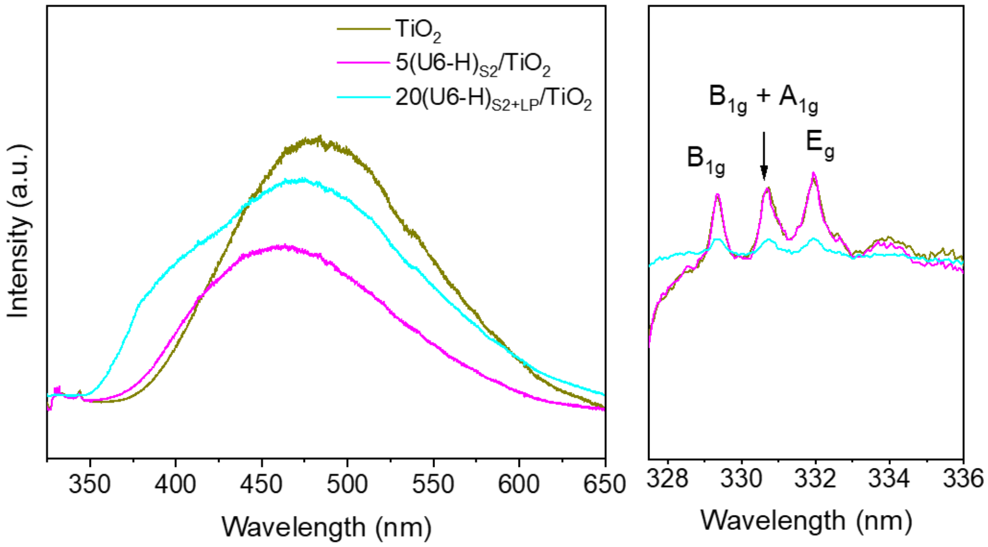

Fig. R3 PL spectra of TiO_2, 5(U6-H)$_{S2}$/TiO_2 and 20(U6-H)$_{S2+LP}$/TiO_2 catalysts. Note that the spectra are normalized based on the measured Raman modes indicated by the inset right image.

Question 2. *Why are the 13C resonances corresponding to CH_3OH and CH_3OOH absent in Figure S22?*

Reply: Thank you for the comment. As shown in the ^{1}H NMR spectrum (**Fig. R4**), CH_3OH and CH_3OOH were detected over 5(U6-H)$_{S2}$/TiO_2 under reaction conditions of 18 bar CH_4 and 2 bar air. However, due to the limited pressure of the $^{13}CH_4$ cylinder available at that stage, the isotope-labeling experiment was initially performed using a mixture of 2 bar $^{13}CH_4$, 2 bar CH_4, and 1 bar air. Under these conditions, because of insufficient sensitivity of NMR ^{13}C analysis, only the major product, $H^{13}CHO$, was observed in the ^{13}C NMR spectrum.

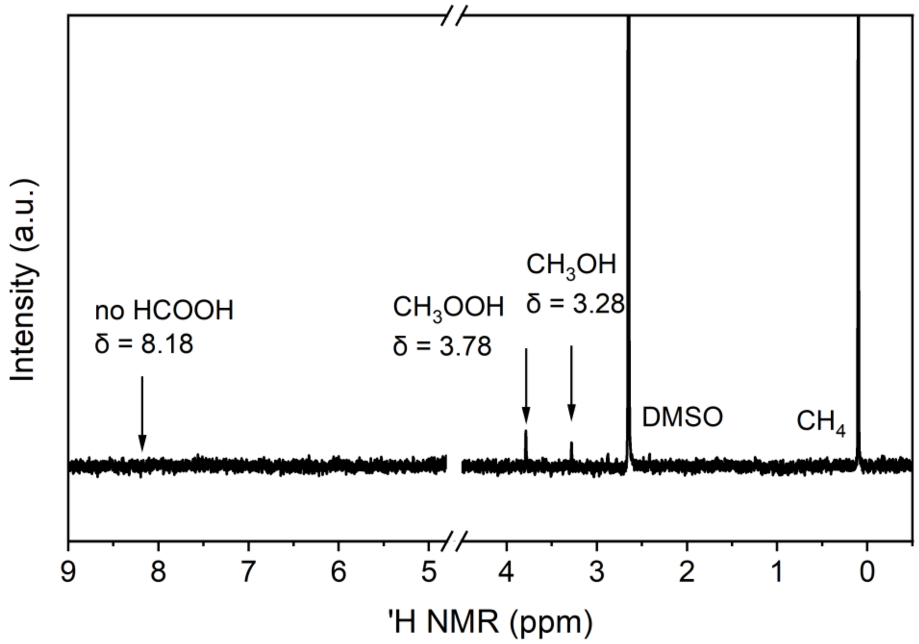

Fig. R4. ^{1}H NMR spectrum of liquid products over (U6-H)$_{S2}$/TiO_2. Test conditions: Pressure (CH_4) = 18 bar, (Air) = 2 bar, Catalyst: 5 mg 5(U6-H)$_{S2}$/TiO_2, 100 mL H_2O, r.t. 500 W Xe lamp illumination for 2 h

To address this limitation, we subsequently purchased a high-pressure $^{13}CH_4$ cylinder and repeated the isotope-labeling experiment using 7 bar $^{13}CH_4$ to increase the yield of ^{13}C-labeled products. As shown in **Fig. R5**, in addition to the $H^{13}CHO$ signal at ~82 ppm, the signal of $^{13}CH_3OOH$ at ~65 ppm can now be observed in ^{13}C NMR at higher methane pressure.

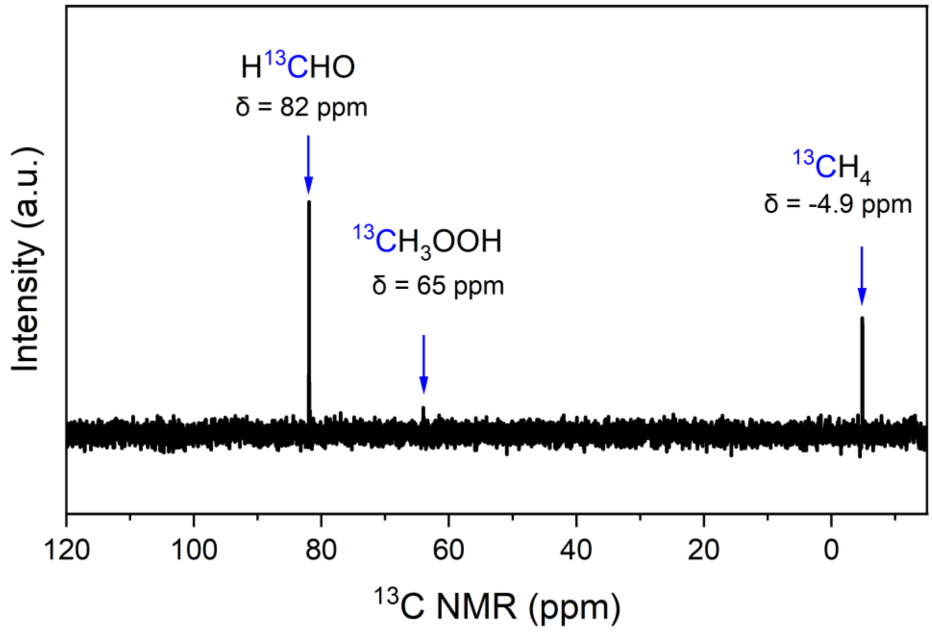

Fig. R5. ^{13}C NMR spectrum of reaction products from $^{13}CH_4$ oxidation over (U6-H)$_{S2}$/TiO_2. Test conditions: Pressure: ($^{13}CH_4$) = 7 bar, (Air) = 1 bar, Catalyst: 5 mg 5(U6-H)$_{S2}$/TiO_2, 100 mL H_2O, r.t. 500 W Xe lamp illumination for 4 h

Accordingly, the updated $^{13}CH_4$ isotope-labeling ^{13}C NMR spectrum has been included as **Fig. S28** in the revised supporting information (Page 32), and the related discussion has been incorporated into the revised manuscript (Page 17, middle).

Question 3. *The manuscript mentions the apparent quantum yield (AQY) in the mechanistic discussion but does not present the corresponding experimental data. The authors are requested to provide the actual AQY values and measurement conditions to substantiate their claims.*

Reply: Thank you for the comment. The apparent quantum yield (AQY) was calculated according to the blow method, which has originally described in the experiment part of manuscript. The AQY at 352 nm and 418 nm was calculated according to the following equation:

$$AQY=\frac{R(electron)\times N_A}{ISt/E_\lambda}\times 100\%$$

where N_A, I, S, and t stand for the Avogadro's constant, light irradiance on the sample

(W/cm^2), irradiation area (9.6 cm^{-2}), and reaction time (3600 s), respectively. E_λ (J) is given by hc/λ (λ = 352 nm or λ = 418 nm). R(electron) represents the number of electrons used in the formation of the products. Since the formations of CH_3OOH, CH_3OH, and HCHO need 1, 3, and 5 photogenerated charges, respectively, R(electron) = n(CH_3OOH) ×1 + n(CH_3OH) ×1 + n(HCHO) × 5. Using the example of a light wavelength of 352 nm, the measured light intensity is 6 mW cm^{-2}, and the amounts of CH_3OOH, CH_3OH, and HCHO are 8.2, 2.3, and 4.7 μmol, respectively. Substituting these values into the equation gives an AQY of 6.38% (**Table R1**).

Table R1. The AQY value of 5(U6-H)$_{S2}$/TiO_2 under different wavelength.

Wavelength (nm)	AQY (%)
352	6.38
418	0

Accordingly, we bolded the AQY calculation in the revised manuscript (Page 29, top) and added the AQY value as **Table S3** in the revised supporting information (Page 43).

Question 4. *In Figure 4b, the authors infer the occurrence of a photo-Fenton reaction; however, this claim lacks experimental substantiation. Please provide supporting evidence based on these cited studies or appropriate references (Appl. Catal. B: Environ. 2025, 361, 124634).*

Reply: Thank you for the comment. To verify whether a photo-Fenton–like process occurs during methane photocatalytic oxidation, we performed a series of reactive-species trapping experiments combined with *in situ* EPR measurements over 5(U6-H)$_{S2}$/TiO_2.

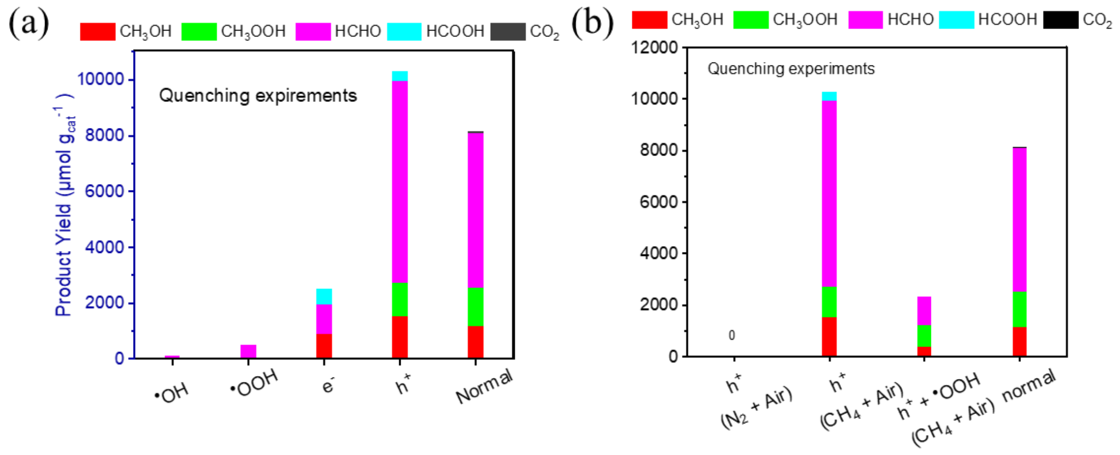

Fig R6. (a), (b) quenching experiments on 5(U6-H)$_{S2}$/TiO_2 catalyst. $K_2S_2O_8$, $Na_2C_2O_4$, salicylic acid, and 1,4-benzoquinone were used as quenchers for e^-, h^+, $^•OH$, and $^•OOH$, respectively.

As shown in **Fig. R6a,** the photocatalytic activity was nearly completely suppressed upon the addition of salicylic acid and 1, 4-benzoquinone, quenchers for $^•OH$ and $^•OOH$, respectively. This suggests that the process proceeds predominantly through a radical-mediated mechanism. When $K_2S_2O_8$ was introduced to trap photogenerated electrons, the reduction of O_2 to protonated $^•OOH$ and H_2O_2 ($^•OH$) species via Haber-Weiss and/or Fenton-like multistep process was significantly hindered resulting in a sharp decline in catalytic activity. In contrast, upon the addition of $Na_2C_2O_4$ as a h^+ sacrificial agent, the proton-coupled electron transfer pathway for O_2 reduction is significantly promoted, leading to a remarkable enhancement in photocatalytic performance. Notably, when methane was replaced with N_2 as the feed gas (**Fig. R6b**), no products were detected. However, when both the h^+ and $^•OOH$ scavengers were introduced, the yield of oxygenates significantly decreased, indicating that the enhanced production of oxygenates originates from oxygen radical–mediated methane activation.

Table R2. The integrated area (a.u.) of the signal for $^{\bullet}OH$ in EPR spectra over TiO_2 and 5(U6-H)$_{S2}$/TiO_2 catalysts

Catalysts	$^{\bullet}OH$
TiO_2	0.83
5(U6-H)$_{S2}$/TiO_2	0.46

Table R3. The integrated area (a.u.) of the $^{\bullet}OOH$ signal in EPR spectra ofTiO$_2$ and 5(U6-H)$_{S2}$/TiO_2 catalysts

Catalysts	$^{\bullet}OOH$
TiO_2	13
5(U6-H)$_{S2}$/TiO_2	29

In situ EPR provides further support. As shown in **Table R2**, the $^{\bullet}OH$ signal intensity for 5(U6-H)$_{S2}$/TiO_2 is almost 2 times weaker than that for TiO_2. When water was replaced by methanol, the $^{\bullet}OOH$ was successfully detected by *in situ* liquid EPR. Notably, the integral area of $^{\bullet}OOH$ on 5(U6-H)$_{S2}$/TiO_2 is 2.3 times higher than that on TiO_2, indicating the favorable generation of $^{\bullet}OOH$ on (U6-H)$_{S2}$/TiO_2 (**Table R3**). A combination of *in situ* measurements and quenching experiments suggests that $^{\bullet}OH$ are primarily produced over (U6-H)$_{S2}$/TiO_2 via the radical-like pathway.

According to the reviewer's suggestion, we cited [Appl. Catal. B: Environ. 2025, 361, 124634] as **Ref.39** in the revised manuscript (Page 36).

Question 5. *In Figure 4b, the authors conclude that electrons dominate the photocatalytic process; however, the role of photogenerated holes is not clearly elucidated. What function do holes play in the overall reaction?*

Reply: Thank you for the comment. In the (U6-H)$_{S2}$/TiO_2 composite catalyst, TiO_2

with a narrower bandgap than U6-H)$_{S2}$ is responsible for the photon adsorption under light irradiation. The generated electrons stay in TiO_2 and eventually migrate to the TiO_2 surface for reduce O_2 to generate protonated $^{\bullet}OOH$ and $^{\bullet}OH$ species via Haber-Weiss and/or Fenton reactions (**Fig. R7**).

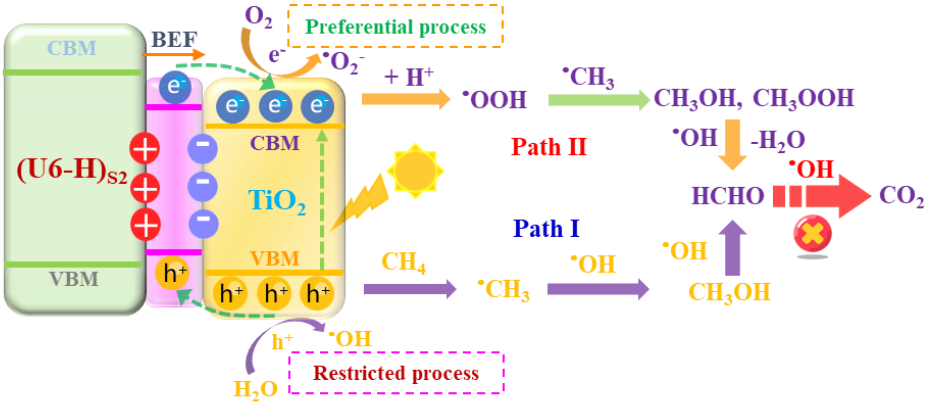

Fig. R7 Plausible mechanism for the photocatalytic oxidation of CH_4 to oxygenates over (U6-H)$_{S2}$/TiO_2

The role of photogenerated holes is clarified as follows. The holes generated in TiO_2 migrate to the (U6-H)$_{S2}$/TiO_2 interfacial region, where the Type-II–like band alignment facilitates their spatial separation from electrons. This interfacial hole transfer effectively suppresses electron–hole recombination, thereby enhancing the photocatalytic efficiency. Moreover, the highly dispersed sub-2 nm MOF crystallites on TiO_2 decrease the VBM and further regulate $^{\bullet}OH$ generation from H_2O oxidation by photogenerated holes avoiding excessing $^{\bullet}OH$ concentrations (**Table R2**). This effectively prevents methane overoxidation and thereby enabling near-complete selectivity toward the desired oxygenates (**Fig. R1**).

Accordingly, we added a corresponding discussion on the function of photogenerated holes in the revised manuscript (Page 24, middle)

Question 6. *According to the band structure shown in Figure 2e, both electrons and holes are transferred from the MOF to TiO2. In that case, why does the DMPO–•OH signal decrease in Figure 4d?*

Reply: Thank you for the comment. The band edge positions of U6-H, TiO_2, and (U6-H)$_{S2}$/TiO_2 were recalculated by correcting the energy levels using the experimentally measured work functions (**Fig. 2e**). Based on this revised band alignment, the photogenerated holes transfer from the valence band of TiO_2 to the (U6-H)$_{S2}$/TiO_2 junction interface, where the Type-II–like band alignment effectively suppresses electron–hole recombination and enhances the photocatalytic activity. (**Fig. R7**). Importantly, the highly dispersed sub-2 nm MOF crystallites on TiO_2 decrease the VBM and moderate $^{•}OH$ generation from H_2O oxidation (**Fig. R8**, **Table R2**), effectively preventing overoxidation and thereby enabling near-complete selectivity toward the desired oxygenates.

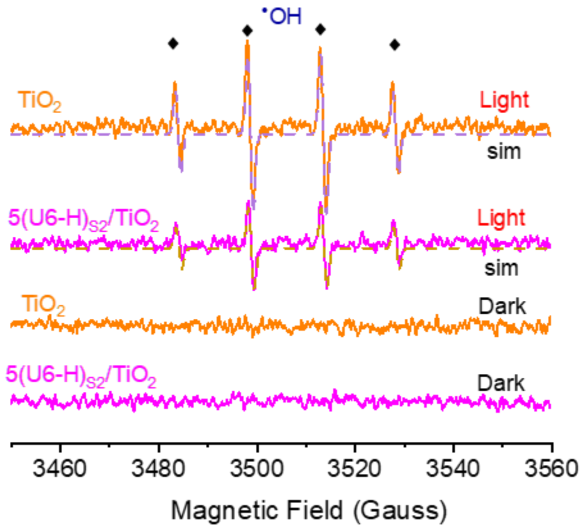

Fig. R8 *in situ* liquid EPR spectra with DMPO as spin trapping of $^{•}OH$ with CH_4 and air on 5(U6-H)$_{S2}$/TiO_2 and TiO_2 catalysts

Question 7. *The manuscript states that CH4 is oxidized by •OH radicals; however, this claim requires supporting evidence. Please provide supporting evidence based on these cited studies or appropriate references (J. Am. Chem. Soc. 2022, 144, 740-750; Chem. Sci 2025, 16, 18652-18659).*

Reply: Thank for the comment. In order to identify the active species involved in the methane photocatalytic oxidation, we have performed a series of reactive-species trapping experiments combined with *in situ* EPR measurements over 5(U6-H)$_{S2}$/TiO_2.

As shown in **Fig. R6a,** the photocatalytic activity was nearly completely suppressed

upon the addition of salicylic acid and 1, 4-benzoquinone, quenchers for •OH and •OOH, respectively. This suggests that the process proceeds predominantly through a radical-mediated mechanism. When $K_2S_2O_8$ was introduced to trap photogenerated electrons, the reduction of O_2 to protonated •OOH and H_2O_2 (•OH) species via Haber-Weiss and/or multistep process was significantly hindered resulting in a sharp decline in catalytic activity. In contrast, upon the addition of $Na_2C_2O_4$ as a h^+ sacrificial agent, the proton-coupled electron transfer pathway for O_2 reduction is significantly promoted, leading to a remarkable enhancement in photocatalytic performance. Notably, when methane was replaced with N_2 as the feed gas, no products were detected. However, when both the h^+ and •OOH scavengers were introduced, the yield of oxygenates significantly decreased, indicating that the enhanced production of oxygenates originates from oxygen radical–mediated methane activation.

In situ EPR provides further support. As shown in **Table R2**, the •OH signal intensity for 5(U6-H)$_{S2}$/TiO_2 is almost 2 times weaker than that for TiO_2. When water was replaced by methanol, the •OOH radicals were successfully detected by *in situ* liquid EPR. Notably, the integral area of •OOH on 5(U6-H)$_{S2}$/TiO_2 is 2.3 times higher than that on TiO_2, indicating the favorable generation of •OOH on (U6-H)$_{S2}$/TiO_2 (**Table R3**). A combination of *in situ* measurements and quenching experiments suggests that •OH are primarily produced over (U6-H)$_{S2}$/TiO_2 via the radical-like pathway.

As suggested by Reviewer, we cited the [J. Am. Chem. Soc. 2022, 144, 740-750; Chem. Sci 2025, 16, 18652-18659] as **Ref. 53** and **54** in the revised manuscript (Page 37).

Question 8. *The labeling of the green signal in Figure 2b appears to be incorrect.*

Reply: Thank you for your carefulness. Accordingly, we corrected the labeling of the green signal in **Fig. 2b** (Page 11).

Reviewer 2: *This manuscript presents a study on enhancing methane photocatalytic oxidation by decorating titania with sub-2 nm Zr-MOF nanocrystals. While the topic is*

of potential interest, the work suffers from significant flaws that critically undermine the validity of its key findings and conclusions. Consequently, I cannot recommend publication of this manuscript due to limited novelty and insufficient scientific rigor. The specific points of concern are detailed below.

Question 1. *The photocatalytic tests employed a 500 W Xe lamp. As indicated in Table S2 and Figure 3b, this light intensity is substantially higher than those typically used in reported literature for methane oxidation (300 W). The observed enhancement in activity is more likely an artifact of the increased photon flux rather than an intrinsic property of the catalyst's structure. Therefore, the performance comparisons made in Table S2 and Figure 3b are invalid and do not meaningfully demonstrate the superiority of the developed catalyst.*

Reply: Thank you for this comment. Use of Xe lamp for photocatalytic methane conversion has been extensively reported [Nat. Commun. 16, 4207 (2025); Nat. Commun. 13, 2806 (2022); J. Am. Chem. Soc. 145, 2698–2707 (2023), J. Am. Chem. Soc. 145, 12357–12366 (2022)]. We would like to clarify that the "500 W" label refers to the electrical input power of the Xe lamp, rather than to the irradiance experienced by the catalyst. This specification is commonly reported in photocatalysis literature and does not reflect the actual photon flux at the sample surface.

To ensure a relatively fair comparison, we measured the real irradiance at the catalyst surface using a calibrated optical power meter. The measured intensity is approximately 350 mW cm^{-2}, which lies within the typical range employed in methane photo-oxidation studies (100~600 mW cm^{-2}) as listed in **Table R4**. Importantly, several representative reports operating at higher irradiance levels (420~600 mW cm^{-2}) show methane oxidation rates below 2500 μmol g_{cat}^{-1} h^{-1} under similar reaction conditions [Nat. Commun. 16, 4207 (2025); Nat. Commun. 13, 6677 (2022); J. Am. Chem. Soc. 145, 8609–8620 (2023); Angew. Chem. Int. Ed. 135, e202302196 (2023)]. Note that our catalyst achieves a markedly higher activity of 6454 μmol g_{cat}^{-1} h^{-1} at a moderate irradiance (350 mW cm^{-2}).

Table R4. Performance comparison of (U6-H)$_{S2}$/TiO_2 with other catalysts in this work and previously representative catalysts for the photocatalytic CH_4 oxidation reaction

Catalysts	Reaction Conditions: Irradiance, Time, Temperature, P_{gas} (bar)	Oxygenates	Oxy. Sel. (%)	Yield μmol/g_{cat}/h	C-H Activation	Ref
Pd–def–In_2O_3	420 nm 3h, RT, 19CH_4, 1O_2	CH_3OH, CH_3OOH, HCHO,	82.5	5000	Radical-like	3
AC–Co_1/PCN_{KOH}	300 W Xe, 2h, RT, 19CH_4, 1O_2	CH_3OH,	87.22	2962	Radical-like	4
q-$BiVO_4$	Hg lamp, 170 mW/cm^2, 10 bar O_2, 10 bar CH_4	CH_3OH, HCHO	86.7	1871	Radical-like	5
Ga_2O_3	300 W Xe, 600 mW/cm^2, 2h, 40 ℃, 19CH_4, 1O_2	CH_3OH, HCHO	87	325.4	Radical-like	6
$Pd_{0.5}V_{0.2}$-TiO_2	300 W Xe, 350 mW/cm^2, 1h, RT, 1O_2, 19CH_4, 3Ar, 2H_2/Ar	CH_3OH, CH_3OOH, HCHO	89.3	5014	Radical-like	7
Ni–NC/TiO_2	300 W Xe, 4h, RT, 19CH_4, 1O_2	CH_3OH, CH_3OOH, HCHO	93	4950	Radical-like	8
TiO_2(001)-C_3N_4	300 W Xe, 450 mW/cm^2, 0.08CH_4, 0.04O_2 165 ul H_2O_2	CH_3OH, HCHO, HCOOH, CH_3CH_2OH,	97	696.3	Radical-like	9
$Au_{0.75}$/ZnO	300 W Xe, 200 mW/cm^2, RT, 2h, 15CH_4, 1O_2	CH_3OH, CH_3OOH	99.1	686	Radical-like	10
Au_x/c-WO_3	300 W Xe, 100 mW/cm^{-2}, RT, 24h, 19CH_4, 1O_2	HCHO	~100	300	Photoholes	11
Cu_1/def-WO_3	LED, 420nm, 2h, RT, 19CH_4, 1O_2	HCHO	~100	2490	Radical-like	12
W_1/PCN-7.5	300 W Xe, 200 mW/cm^{-2}, 5h, RT, 5CH_4, H_2O	CH_3OH	~100	991.2	Radical-like	13
UION-Cu(OH)	300 W Xe, 5h, RT, 9CH_4, 3O_2	HCHO	~100	2750	Radical-like	14
Hetero-ZnO/Fe_2O_3	300 W Xe, 100 mW/cm^{-2}, RT, 1.5h, 1CH_4, H_2O	CH_3OH, CH_3OOH	~100	119	Photoholes	15
Au_1/BP	300 W Xe, 380 mW/cm^{-2}, 2h, 90 ℃, 30CH_4, 3O_2	CH_3OH	>99	56.8	Photoholes	16
BN	300 W Xe, 600 mW/cm^2, 2h, 60 ℃, 1CH_4, 1O_2	CH_3OH, HCHO	~100	130.2	Photoholes	17
fl-CN-530	300 W Xe, 200 mW/cm^2, 3h, RT, 19CH_4, 1O_2	CH_3OH, CH_3OOH, HCHO	99.2	790	Radical-like	18
MoO_x–TiO_2	300 W Xe, 420mW/cm^2, 2h, RT, 19CH_4, 1O_2	CH_3OH, CH_3OOH, HCHO, HCOOH	~100	1900	Radical-like	19
5(U6-H)$_{S2}$/TiO_2	**500 W Xe, 350mw/cm^2, 0.5h, RT, 18CH_4, 2Air**	**CH_3OH, CH_3OOH, HCHO**	**~100**	**6454.3**	**Radical-like**	**This work**

These observations indicate that the enhanced performance is not a consequence of elevated photon flux but arises from the intrinsic properties of the engineered

heterostructure. All activities reported in **Table R4** and **Fig 3b** were obtained under identical illumination conditions and normalized per gram of catalyst, ensuring the internal validity of the comparisons. Overall, the superior performance observed in this work reflects the catalyst's intrinsic reactivity rather than the lamp wattage or irradiation intensity.

Accordingly, we added the irradiance of the listed catalysts of **Table S2** in the revised supporting information (Page 42) and added corresponding discussion in the revised manuscript (Page 15, bottom).

Question 2. *The authors utilized a broad-spectrum light source (300-100 nm). However, the UV-Vis spectra (Figure 2d) confirmed that both TiO2 and 5(U6-H)S2/TiO2 primarily absorbed only in the UV region. The contribution of Vis and IR portions of the spectrum to the reported activity remains unaddressed. Since most literature in this field employed UV light, the apparent performance improvement in this work could potentially stem from thermal or other effects induced by Vis/IR irradiation, rather than from the proposed structural modifications of the catalyst.*

Reply: Thank you for the comment. Due to its broad and continuous emission spectrum (from 300 to 1100 nm), the Xe lamp is widely used in photocatalytic methane conversion [Nat. Commun. 16, 4207 (2025); Nat. Commun. 13, 2806 (2022); J. Am. Chem. Soc. 145, 2698–2707 (2023), J. Am. Chem. Soc. 145, 12357–12366 (2022)]. As reviewer mentioned, both TiO_2 and 5(U6-H)$_{S2}$/TiO_2 primarily absorbed only in the UV region (**Fig. R9**).

Notably, a high apparent quantum yield (AQY) of 6.38% was achieved on 5(U6-H)$_{S2}$/TiO_2 under monochromatic irradiation with a wavelength of 352 nm (**Table R1**). In contrast, the calculated AQY under 418 nm light is 0%, consistent with the UV–vis absorption spectrum of 5(U6-H)$_{S2}$/TiO_2. Only traces of oxygenate products were detected without UV light or under visible light ($\lambda > 400$ nm, **Fig. R10**). We have used the cooling system to keep the reaction temperature at 25 °C during reaction to prevent the IR generated heat in system. These results indicate that the Vis/IR irradiation has a

negligible contribution to the reaction.

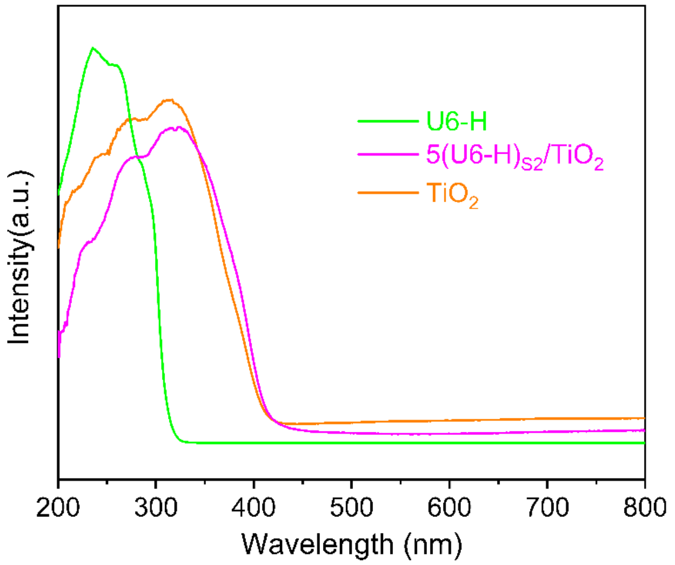

Fig R9. Diffuse reflectance UV-Vis spectra on TiO_2, U6-H, and $5(U6\text{-}H)_{S2}/TiO_2$ catalysts

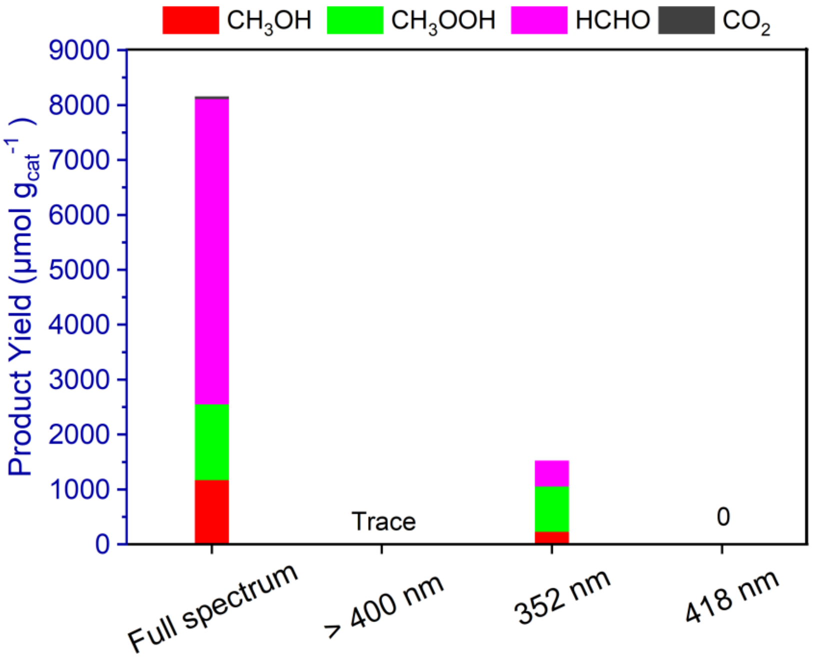

Fig. R10. Photocatalytic CH_4 oxidation performance of $5(U6\text{-}H)_{S2}/TiO_2$ under different wavelength

Accordingly, we added the results obtained with visible light (**Fig. S29,** Page 33) and corresponding discussion to the revised manuscript (Page 17, bottom)

Question 3. *The TEM images provided (Fig. 1b-c, S2b) lack the clarity and resolution necessary to conclusively identify and confirm the presence of Zr-MOF nanocrystals with sizes below 2 nm. To substantiate this critical claim, the authors must provide a representative particle size distribution derived from multiple TEM images, statistically validating the nanocrystal size.*

Reply: Thank you for the comment. As shown in **Fig. 1b** and **c**, ultrasmall MOF nanocrystals with a size of approximately < 2 nm are anchored on the surface of TiO_2. In addition, energy-dispersive X-ray spectroscopy mapping (EDX-mapping) confirms that the Zr element originating from $(U6\text{-}H)_{S2}$ is uniformly dispersed across the TiO_2 surface (**Fig. 1e**). In contrast, pure TiO_2 has a clean surface without any MOF particle (**Fig. S2**).

Following the reviewer's suggestion, additional HRTEM images were collected from multiple regions of the catalyst to substantiate the size of the supported MOF nanocrystals. As shown in **Fig. R11a–c**, a large number of ultrasmall MOF nanocrystals are uniformly dispersed on the TiO_2 surface. Statistical analysis based on multiple TEM images demonstrates that the average particle size is below 2 nm (**Fig. R11d**).

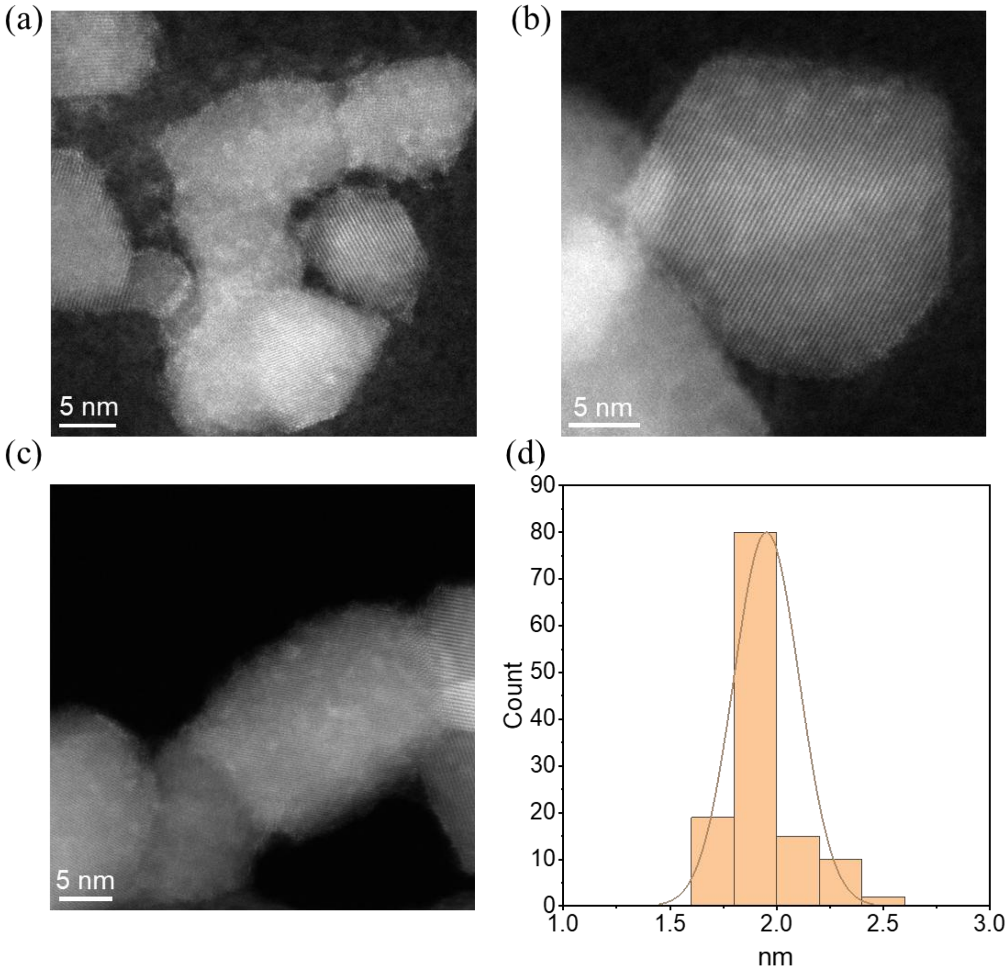

Fig. R11 (a-c) Representative AC-HAADF-TEM, (d) particle size distribution of 5(U6-H)$_{S2}$/TiO_2 catalyst.

Accordingly, we added **Fig. R11** as **Fig. S3** in the revised supporting information (Page 7) and corresponding discussion in the revised manuscript (Page 8, bottom)

Question 4. *The XRD patterns are presented without reference to standard diffraction cards. This omission makes it impossible for the reader to verify the crystal phases.*

Reply: Thank you for the comment. As shown in **Fig. R12**, pristine U6-H exhibits two prominent diffraction peaks at 2θ = 7.5° and 8.6°, corresponding to the (111) and (200) crystal planes [cm1022882_si_001.cif], respectively. P25 TiO_2 exhibits two prominent diffraction peaks at 25.3° and 27.4°, corresponding to the anatase (101) and rutile (100) planes [Anatase: JCPDS 21-1272; Rutile: JCPDS 21-1276], confirming that it contains

a mixed anatase–rutile phase. In the (U6-H)/TiO_2 composites, the characteristic peaks of U6-H gradually increase with the U6-H content, confirming the coexistence of both U6-H and TiO_2 phases in the composites.

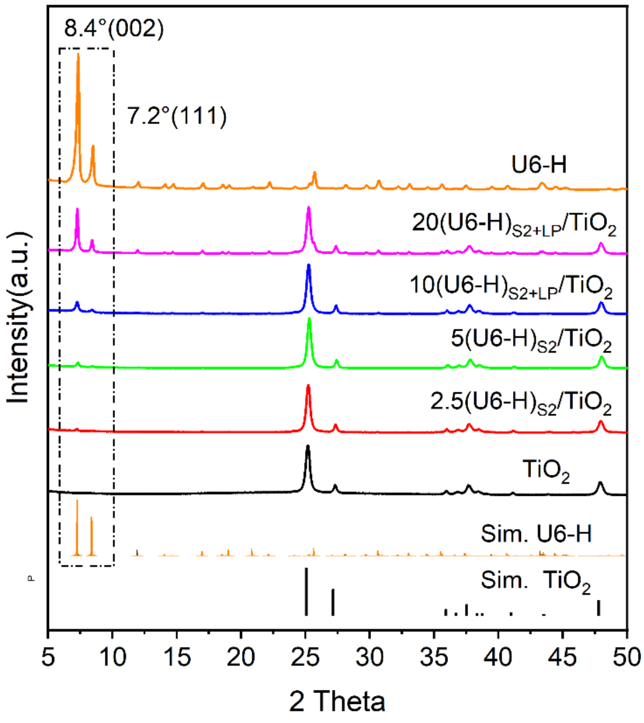

Fig. R12 XRD pattern of U6-H, TiO_2, 2.5(U6-H)$_{S2}$/TiO_2, 5(U6-H)$_{S2}$/TiO_2, 10(U6-H)$_{S2+LP}$/TiO_2, 20(U6-H)$_{S2+LP}$/TiO_2 catalysts, together with the simulated diffraction patterns of U6-H (cm1022882_si_001.cif) and P25 TiO_2 (Anatase: JCPDS 21-1272; Rutile: JCPDS 21-1276).

Accordingly, we added the simulated standard XRD pattern both of U6-H and P25 TiO_2 in **Fig S1** (Page 5), and corresponding discussion to the revised manuscript (Page 7, middle)

Question 5. *To properly compare the Lewis acid sites, Pyridine-IR spectra for the pristine U6-H should be included.*

Reply: Thank you for this suggestion. Following the reviewer's suggestion, Pyridine-IR spectra of the pristine U6-H were measured to enable detection of Lewis acid sites (**Fig. R13**). After pyridine adsorption, pristine U6-H exhibits relatively weaker pyridine

adsorption bands at around 1438 and 1605 cm^{-1}, which are assigned to pyridine coordinated to $Zr^{\delta+}$ Lewis acid sites associated with Zr-oxo nodes [Catal. Sci. Technol. 8, 1134-1141 (2018)].

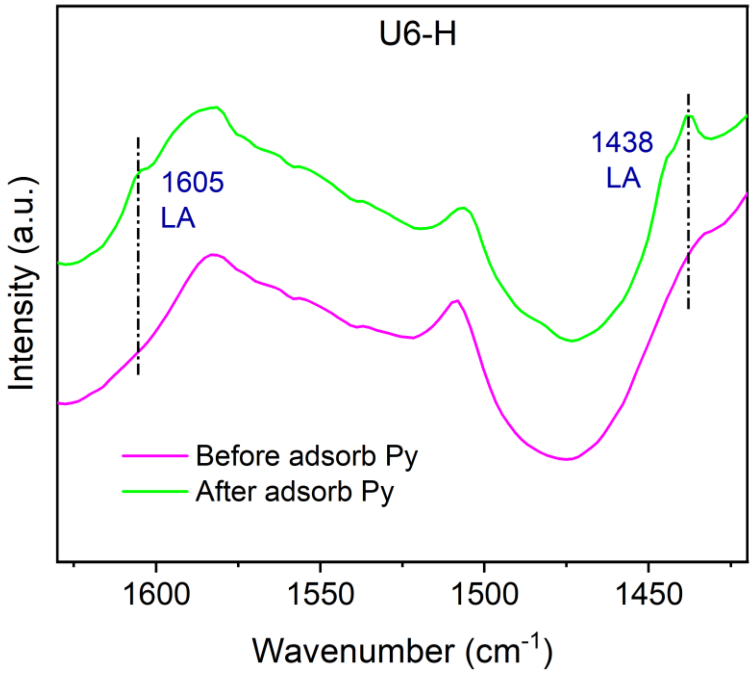

Fig. R13 Pyridine-IR spectra on U6-H catalyst; Py: pyridine; LA: Lewis acid

For comparison, the characteristic bands of pyridine coordinated to Lewis acid $Ti^{\delta+}$ sites on TiO_2 (1445 and 1605 cm^{-1}), are markedly weakened after the introduction of ultrasmall U6-H nanocrystals (**Fig. 2b**). This observation indicates a significant decrease in the density of accessible Ti-based Lewis acid sites, consistent with their involvement in interfacial coordination during heterojunction formation. Overall, the weak pyridine adsorption signals of pristine U6-H confirm its intrinsically low Lewis acidity, demonstrating that the observed suppression of Lewis acidity in 5(U6-H)$_{S2}$/TiO_2 primarily originates from the modification of TiO_2 surface sites.

Accordingly, we added the Pyridine-IR spectra on U6-H catalyst as **Fig S5** in the revised supporting information (Page 9), and corresponding discussion to the revised manuscript (Page 10, top)

Question 6. *The UV-Vis spectra (Figure 2d) appear to show enhanced absorption in*

the UV region for pure TiO2, not for the 5(U6-H)S2/TiO2. This observation contradicts the narrative of enhanced light harvesting due to the composite structure.

Reply: Thank you for the comment. The statement of "enhanced light harvesting" in our manuscript refers to the slight red-shifted absorption edge in the 350–400 nm region originating from the introduction of sub-2nm U6-H nanocrystals. More importantly, the improved photocatalytic activity results from the more efficient utilization of photogenerated carriers, as supported by PL quenching (**Fig. S11**), reduced charge-transfer resistance in EIS (**Fig. 2f**), enhanced photocurrent signals (**Fig. 2g**), and longer-lived electrons revealed by fs-TAS (**Fig. 2i**). Therefore, **Fig. 2d** is fully consistent with the mechanism and does not contradict the narrative of enhanced light harvesting for the composite structure.

Question 7. *A significant discrepancy exists between the UV-Vis, Band gap, PL spectra and the EIS, PC spectra. The former set shows minimal differences between TiO2 and 5(U6-H)S2/TiO2, while the latter indicates pronounced changes. The authors must provide a plausible explanation for this inconsistency.*

Reply: Thank you for the comment. UV–Vis (**Fig. 2d**) and steady-state PL (**Fig. S11**) are measured under dry solid-state conditions. They mainly probe the intrinsic optical absorption and bulk radiative recombination. As the ultrasmall U6-H nanocrystals are introduced and primarily act as an interfacial modifier rather than a bulk dopant, they do not substantially alter the bulk electronic structure of TiO_2. Consequently, some changes are observed in the absorption edge and PL intensity.

In contrast, electrochemical impedance spectroscopy (EIS, **Fig. 2f**) and photocurrent measurements (**Fig. 2g**) are conducted in a liquid–solid system using 0.5 M Na_2SO_4 electrolyte, where interfacial charge transfer processes dominate. Under these conditions, Fermi-level equilibration, surface band bending, and electrolyte-induced interfacial dipoles become critical factors governing charge separation and transport [Appl. Surf. Sci. 387, 183 (2016); Appl. Catal. A: Gen. 433, 81 (2012)]. The formation of the MOF–TiO_2 heterojunction therefore leads to a pronounced reduction in charge-

transfer resistance and a substantial enhancement in photocurrent density. The electrolyte environment amplifies interfacial charge-separation effects that are not accessible by dry-state UV–Vis or PL measurements. Hence, the modest changes observed in optical spectra and more pronounced improvements in EIS and photocurrent responses are consistent and complementary, reflecting the fundamentally different physical processes probed under solid-state and liquid–solid conditions.

Question 8. *For a comprehensive comparison of charge carrier behavior, the pump-probe decay curves for the U6-H sample should be provided.*

Reply: Thank you for this suggestion. As requested, we performed fs-TAS measurements on pristine U6-H to enable a direct comparison of charge carrier behavior. The pump–probe decay curves of pure KBr and the U6-H/KBr pellet are shown in **Fig. R14**. Both KBr and U6-H/KBr exhibit only a very fast decay component with a half-life shorter than 5 ps, which can be attributed to the instantaneous electronic response of the materials. Notably, no long-living transient signal (> 100 ps), commonly associated with free or shallowly trapped electrons, can be detected for U6-H in the 330–400 nm excitation range. Such long-living charge carrier signatures are clearly observed for TiO_2-containing samples in **Fig. 2i**. They have been widely reported for photoexcited semiconductors in the literature [Phys. Chem. Chem. Phys., 2007, 9, 1453–1460; Chem. Phys. Lett., 2010, 500, 309–312].

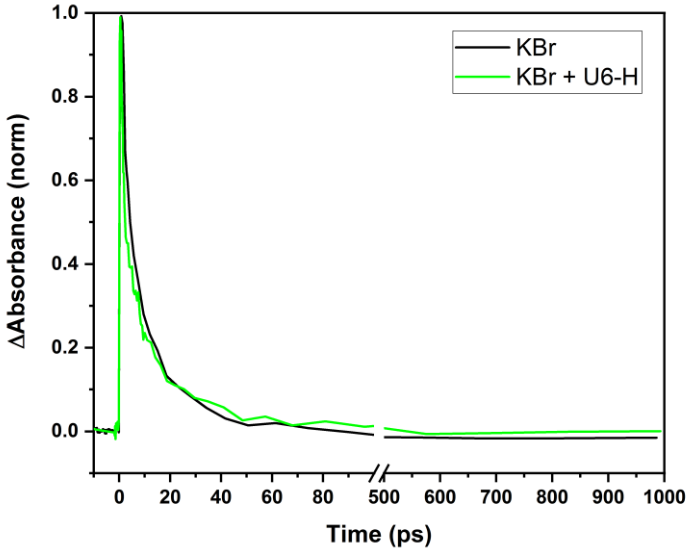

Fig. R14 Pump–probe kinetic traces of pure KBr and of the U6-H + KBr pellet. The measurements were using a 330 nm pump and a probe centered at 4750 nm, with an excitation energy of 80 nJ/pulse.

These results indicate that pristine U6-H cannot be effectively photoexcited in the 330–400 nm spectral region and therefore does not generate long-living charge carriers detectable in the mid-IR transient absorption signal. Consequently, the charge carriers observed in 5(U6-H)$_{S2}$/TiO_2 originate from the excitation of the TiO_2 component. The modification of electron lifetime observed in the composite catalyst can thus be attributed to the interfacial interaction between U6-H nanocrystals and TiO_2, rather than to direct photoexcitation of U6-H.

Accordingly, we added this fs-TAS spectra on U6-H catalyst in **Fig S19** in the revised supporting information (Page 23), and corresponding discussion to the revised manuscript (Page 14, middle).

Reviewer 3: *In this paper the authors present the work titled "In-situ Decorating Titania with Sub-2 nm Zr-MOF Nanocrystals for Quantitative Methane Photocatalytic Oxidation". In this work they produced a heterojunction photocatalyst, where ultrasmall sub-2 nm Zr MOF ((U6-H)S2) nanocrystals were grown in situ on the surface of P25 TiO2 particles. These photocatalysts were capable of oxidising CH4 in*

air with high selectively to CH3OH, CH3COOH and CH2O (with little over oxidation to CO2). The show that this catalyst achieves state of the art performance in terms of product selectivity (i.e. near 100%) and activity (i.e. 6454 μmol gcat^{-1} h^{-1}). The materials are well characterized by a whole suite of methods, including the use of various scavengers (and other conditions) to verify the activity of their catalysts.

The work is of high quality, and I would be happy to accept this after minor revisions, with my primary point of disagreement being their interpretation of XPS and UPS data, which can be used to construct a band energy diagram. My analysis indicates that a Type II alignment will form, where hole carriers will be encourage to migrate from the TiO2 layer into the MOF, which is detailed in Q12 below.

***T1).** "as cocatalysts/promoters for important semiconductor, such TiO2" should be "as cocatalysts/promoters for important semiconductors, such TiO2"*

Reply: Thank you for the comment. Accordingly, we revised the related phrase as "as cocatalysts/promoters for important semiconductors, such TiO_2" in the revised manuscript (Page 5, bottom).

***T2).** "using air as oxidant." Should be "using air as an oxidant."*

Reply: Thank you for the comment. Accordingly, we revised the related phrase as "using air as an oxidant" in the revised manuscript (Page 6, bottom).

***T3).** Fig. S1 should state what is the meaning of 2.5, 5, 10 and 20 in the composite samples.*

Reply: Thank you for the comment. Accordingly, we rewritten the meaning of 2.5, 5, 10 and 20 of the composite samples in the revised supporting information (**Fig. S1**, Page 5).

***T4).** The caption of Figure 1 requires some revision. Image d) is incorrectly labelled as EDS mapping (this should be image e), which is not mentioned in the caption). And the arrows drawn in figures c) and d) should be described (which I can see are pointing to*

MOF sites, however, not all MOF sites are pointed to, so the authors should make clear that only some MOF sites are point to as examples).

Reply: Thank you for the comment. Accordingly, we revised the figure of EDS mapping as **Fig.1e**. And we also revise the yellow arrows pointing to MOF species in **Fig. 1c and 1d** to make the mark more clearly (Page 8).

T5). *The caption of Figure S3 says that the N2 sorption data is of "various (U6-H)/TiO2 catalysts" however, this is rather of a) TiO2, b) 5(U6-H)s2/ TiO2, c) 20(U6-H) and d) TiO2. This error is present in various figure captions. Please revise this error throughout to be more precise in your captions of what is being displayed.* **Reply:** Thank you for the comment. Accordingly, we revised the captions of **Fig. S4** in the revised supporting information **(**Page 8**)**.

T6). *In Figure 2, the image (d) says "wavelgenth" but rather should say "wavelength". Also, (b) says "wavenumbers" but convention is to write "wavenumber". Also, in the caption you write, "UV-vis spectra", please be more specific, as I believe that these are "diffuse reflectance UV-vis spectra". You then write, "photocurrent spectra". These are not spectra, as you did not investigate a range of discrete wavelengths. Rather, this is simply some chronoamperometry measurement of the photocurrent density with light on/off cycles. Please state the applied potential and light source used. And you later write, "(g) the BEF intensity for on TiO2.." which should rather be "(h) the BEF intensity for TiO2…"*

Reply: Thank you for the comment. Accordingly, we have corrected the bottom title both of **Fig. 2b** and **2d** as "wavenumber" and "wavelength", respectively. We have corrected the caption of **Fig. 2d, 2g** and **2h** as "diffuse reflectance UV-Vis spectra", "the photocurrent density with light on/off cycles", and "the BEF intensity for TiO_2 and $5(U6\text{-}H)_{S2}/TiO_2$ catalysts" in the revised manuscript, respectively (Page 11).

T7). *"To further unveil the charge migration and transfer efficiency in composite"*

should be "To further unveil the charge migration and transfer efficiency in the composite"

Reply: Thank you for the comment. Accordingly, we revised the corresponding statement "To further unveil the charge migration and transfer efficiency in the composite" in the revised manuscript (Page 12, bottom).

T8). *Figure S11 has misspelt wavelength*

Reply: Thank you for the comment. Accordingly, we have corrected the bottom title of **Fig. S15** as "wavelength" in the revised supporting information (Page 19).

T9). *"reaching 8158.5 μmol gcat-1 within 2 h", the -1 is not all superscript as it should be.*

Reply: Thank you for the comment. Accordingly, we have corrected the -1 as superscript in the corresponding sentence "reaching 8158.5 μmol g_{cat}^{-1} within 2 h" in the revised manuscript (Page 15, middle).

T10). *In Figure 4, the labels "wavenumbers" should instead be "wavenumber"*

Reply: Thank you for the comment. Accordingly, we have corrected the bottom title both of **Fig. 4e** and **4f** as "wavenumber" in the revised manuscript, respectively (Page 19).

Question 1. *In the introduction, the authors use the term, "delivering promoter-level". I request they add some information on what they mean by this term.*

Reply: Thank you for the comment. The term "promoter-level" was used to indicate that the enhancement of catalytic rate and selectivity achieved by downsizing the MOF nanocrystals to the sub-2 nm regime is comparable to the performance improvement typically brought about by adding a promoter in heterogeneous catalysis. In other words, even without introducing an external promoter element, the nanoscale interface engineering itself delivers a "promoter-like" effect.

Accordingly, we clarified this point in the revised manuscript as "thereby achieving rate and selectivity enhancements comparable to those typically induced by promoter addition ("promoter-level" improvement)" (Page 6, top).

Question 2. *Some clarification should be given from the outset of the results and discussion section as to the series of MOF/TiO2 samples produced, and what is the meaning of the S2 subscript in some samples (I presume due to the MOF being sub-2nm in size), whereas in others it is not present (again, I presume due to the MOF being larger than sub-2nm in size).*

Reply: Thank you for the comment. We agree that clarification of the sample nomenclature is important and have added an explanation at the beginning of the Results and Discussion section.

In our work, the subscript S2 is used to denote that the U6-H MOF species are present exclusively as ultrasmall sub-2-nm nanocrystals on the TiO_2 surface. For example, in the sample 5(U6-H)$_{S2}$/TiO_2, all U6-H components are confined to the sub-2-nm size regime. In contrast, when sub-2-nm U6-H nanocrystals coexist with larger U6-H nanoparticles, the samples are labeled with S2+LP. For example, 20(U6-H)$_{S2+LP}$/TiO_2 contains both the sub-2-nm nanocrystals and additional larger nanoparticles, which appear at higher MOF loadings.

Accordingly, this clarified nomenclature has now been updated in the revised manuscript (Page 27, top).

Question 3. *The authors state that "both 5(U6-H)S2/TiO2 and TiO2 exhibit a cubic morphology". I would re-phrase this statement. There are a variety of shapes seen, which is typical of P25*

Reply: Thank you for the suggestion. Accordingly, we have re-phrased this statement "**Fig. 1b, c** and **Fig. S2a, b** show that both 5(U6-H)$_{S2}$/TiO_2 and TiO_2 exhibit a aggregated nanoparticles morphology with an average size of ~20 nm." in the revised manuscript (Page 8, bottom).

Question 4. *The label on Figure S6 should not be "band gap spectra" but rather, a Tauc plot of diffuse reflectance data. Also, the fact that you have plotted the square of (Ahv) means that you are determining the direct allowed transition. In P25, the majority phase is anatase, where the lowest energy transition is an indirect allowed transition, and therefore, one should plot the square root of (Ahv).*

Reply: Thank you for the comment. We appreciate the reviewer's clarification regarding the proper labeling and interpretation of **Fig. S8**. In the revised supporting information, we have corrected the label from "band gap spectra" to "Tauc plot of diffuse reflectance data".

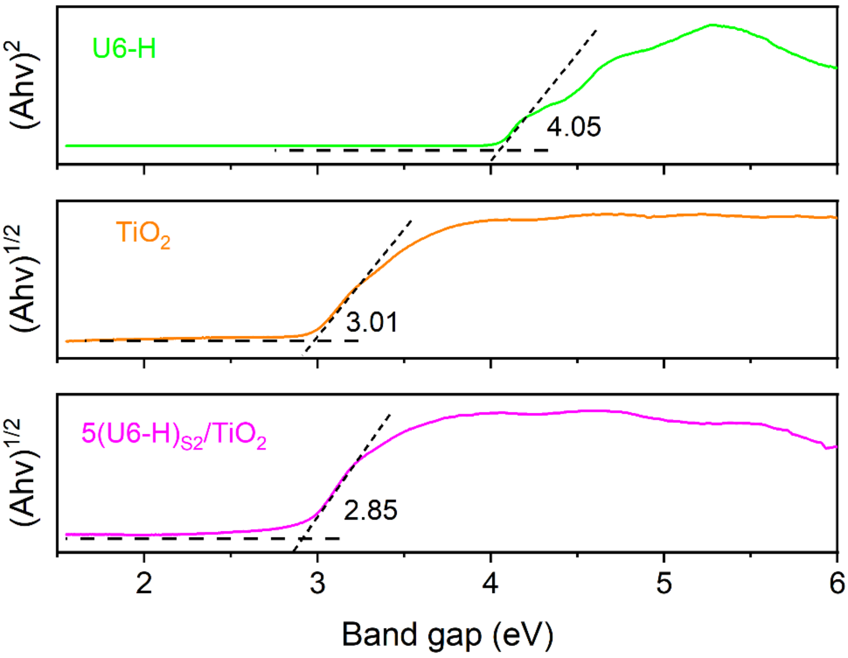

Fig. R15 Tauc plot derived from diffuse reflectance spectra of various (U6-H)/TiO_2 catalysts

As shown in **Fig. R15**, after taking into account the indirect band-gap nature of TiO_2, we re-evaluated the band gaps of TiO_2 and $(U6\text{-}H)_{S2}/TiO_2$ using the indirect Tauc method based on $(Ahv)^{1/2}$. The resulting band-gap values of 3.01 and 2.85 eV for TiO_2 and $(U6\text{-}H)_{S2}/TiO_2$, respectively, confirm that the incorporation of sub-2 nm U6-H indeed leads to a reduced band gap.

Accordingly, we updated the **Fig. S8** in the revised supporting information (Page 12)

and revised the corresponding part in the revised manuscript (Page 12, top).

Question 5. *Similar to above, Figure S7 does not display "VBM spectra", but rather, low binding energy XPS measurements of the VBM energy region of your catalysts. Please revise.*

Reply: Thank you for the suggestion. Accordingly, we revised the label of **Fig. S10** in the revised supporting information (Page 14).

Question 6. *In the caption of Figure S8, please state the excitation wavelength of light.*

Reply: Thank you for the comment. The excitation wavelength of photoluminescence spectroscopy (PL) is 325 nm, which have originally provided in the experiment part. Accordingly, we further add the excitation wavelength of PL in the caption of **Fig. S11** in the revised supporting information (Page 15).

Question 7. *The authors write, "Compared to pristine U6-H, the 5(U6-H)S2/TiO2 composite exhibits enhanced absorption in the UV region, with a slightly extended absorption range than TiO2. This enhancement can be attributed to the interfacial interaction between TiO2 and ultrasmall U6-H nanocrystal. The band gap energies (Eg) were calculated using Tauc' plot (Fig. S6)… etc." In my view, the band edge of TiO2 before and after the addition of the MOF changes only marginally, and likely, within the error of the measurement. And regarding the bandgap values determined from your Tauc plots (which should be redone using the correct square root rather than the square), these values are also within error, given the uncertainty in where to extrapolate each band edge to the energy axis. Therefore, please tone down your statements regarding bandgap reductions.*

Reply: Thank you for the comment. As suggested, we re-evaluated the UV–Vis diffuse reflectance data and repeated the measurements for both TiO_2 and $5(U6\text{-}H)_{S2}/TiO_2$. The new results (**Fig. R16**) confirm a slight but reproducible extension of the absorption edge for the composite compared with bare TiO_2. The magnitude of this shift is small

and does not indicate a substantial change in the intrinsic bandgap.

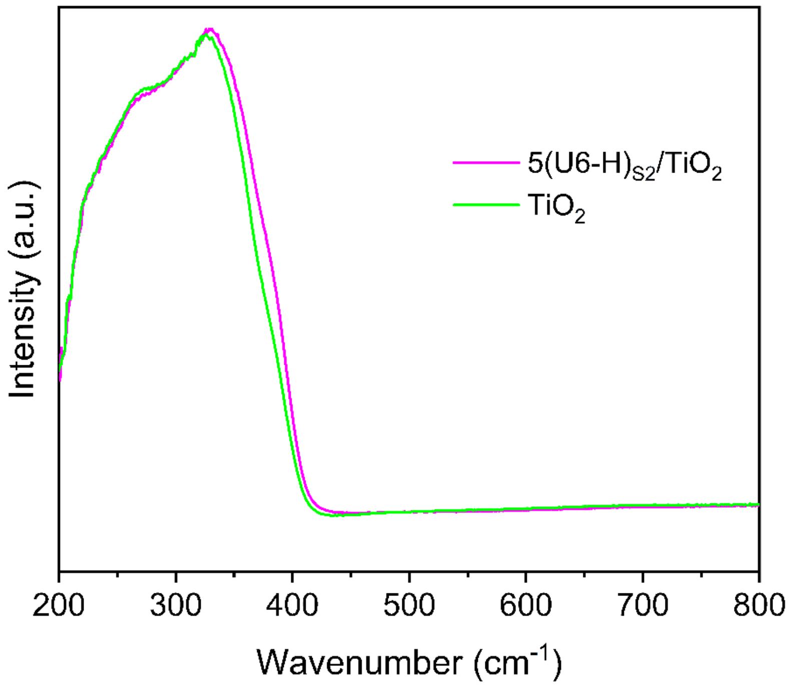

Fig. R16 diffuse reflectance UV-Vis spectra over TiO_2 and $5(U6\text{-}H)_{S2}/TiO_2$

Importantly, both direct and indirect Tauc analyses yield bandgap values that are essentially unchanged upon incorporation of the ultrasmall U6-H nanocrystals. Therefore, in the revised manuscript, we have toned down our discussion and now describe the UV–Vis result as indicating only a minor and reproducible absorption-edge extension, which may arise from subtle interfacial interactions or scattering effects, rather than a meaningful bandgap reduction. The enhanced photocatalytic performance is thus attributed primarily to improved charge separation and interfacial charge transfer, as supported by PL (**Fig. S8**), EIS (**Fig. 2f**), PC (**Fig. 2g**), and fs-TAS (**Fig. 2i**) measurements.

Question 8. *The authors write, "the valence band maximum (VBM) of (U6-H)S2/TiO2 measured by XPS is 2.66 eV vs. NHE, which is significantly lower than that of TiO2 (2.92 eV, vs. NHE) and U6-H (3.56 eV, vs. NHE)". In my view, these values are not vs the NHE scale. They were measured in an XPS instrument under vacuum, and likely, these values are therefore vs the Fermi level (EF) of each material. As such, please*

modify Figure 2(e) accordingly, where the y-axis should be changed to "Potential vs the EF of each material". What this graph and XPS shows us, in my view, is a reduction in energy between the VBM and EF upon addition of the MOF to TiO2, indicating a flow of donor density from TiO2 into the MOF. You later write, "This observation is consistent with the result that, after the introduction of sub-2 nm U6-H, the VBM of (U6-H)S2/TiO2 shifts to a lower level compared to TiO2 (2.56 vs. 2.92 eV, Fig. 2e), thereby weakening its ability to oxidize H2O into •OH". Which based on the above, I feel should be deleted (as one has not determined their VBM with respect to the NHE, one cannot make the above statement".

Reply: Thank you for the comment. We agree that valence band spectra measured by XPS under vacuum conditions directly provide the energy separation between the valence band maximum (VBM) and the Fermi level (Ef), rather than potentials versus the NHE scale. To better interpret the band structure of the photocatalysts in an electrochemical context, the VBM versus NHE was further estimated by combining the experimentally measured work function (Φ) with the VBM–Ef separation obtained from UPS measurements: E_{VB}, NHE = Φ + (Ef − VBM) − 4.44.

It should be noted that, for powder samples such as TiO_2 and MOF-based composites, the accurate determination of the work function by UPS remains experimentally challenging due to their poor conductivity and residual surface charging effects, even when an external bias is applied. As a result, the absolute VBM values referenced to NHE may deviate from ideal theoretical values. Nevertheless, under identical measurement conditions, this method remains reliable for comparing relative VBM shifts among different samples, as commonly accepted in previous studies [J. Phys. Chem. C, 125, 1902 (2021)].

Based on this analysis, as shown in **Fig. R17**, the valence band maximum (VBM) of 5(U6-H)$_{S2}$/TiO_2 is estimated to be 1.44 eV versus NHE, which is lower than those of pristine TiO_2 (1.84 eV vs. NHE) and U6-H (1.78 eV vs. NHE), indicating strong interfacial electronic coupling and the formation of a heterojunction between TiO_2 and the highly dispersed sub-2 nm U6-H crystallites. Hence, upon the introduction of sub-

2 nm U6-H, the VBM of 5(U6-H)$_{S2}$/TiO_2 shifts downward relative to TiO_2 (1.84 vs. 1.44 eV), implying a reduced oxidative driving force for H_2O oxidation to $^{\bullet}$OH.

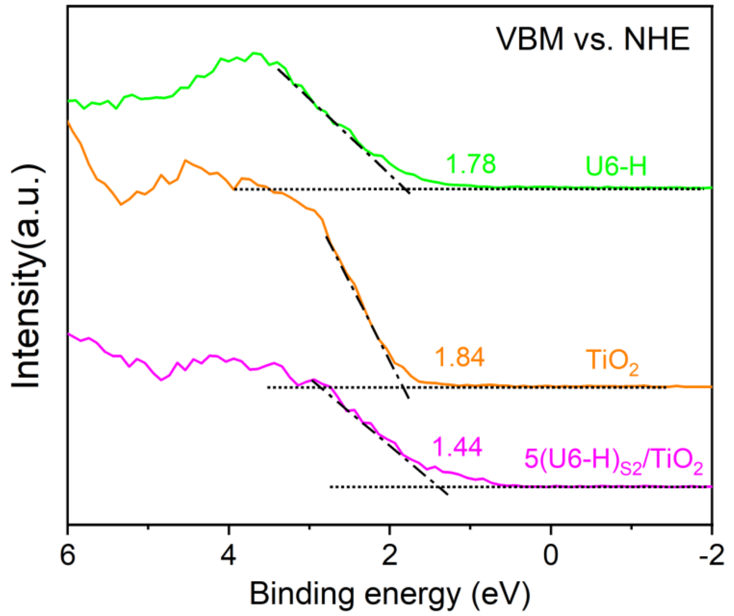

Fig. R17. Low binding energy XPS measurements of the VBM energy region of TiO_2, U6-H, and 5(U6-H)$_{S2}$/TiO_2 catalysts. VBM versus NHE: combining the experimentally measured work function (Φ) with the VBM–Ef separation obtained from UPS, according to: E_{VB}, NHE = Φ + (Ef − VBM) − 4.44.

Accordingly, we updated the VBM vs. NHE in **Fig. S10** in the revised supporting information (Page 24) and added corresponding discussion in the revised manuscript (Page 12, top).

Question 9. *I would remove your surface charge density analysis from the work. You write, "Besides, as displayed in Figs. S9 and S10, the surface charge density of 5(U6-H)S2/TiO2 was calculated to be higher than that of TiO2 (58.0 vs 3.98 μC cm−2)". Now looking at the data, if it were surface charge, you would also see discharging in the dark. For the composite, you do not see that, however, for the TiO2 sample, you do. Therefore, there is more photo-induced capacitance (i.e. surface charging) in the TiO2 sample than in the composite.*

Reply: Thank you for the comment. We respectfully clarify that the "surface charge

density" used in our work follows the definition introduced by Kanata et al. and adopted in subsequent studies for evaluating built-in electric fields (BEF) [Nat. Commun. 12, 4952 (2021); Angew. Chem. Int. Ed. 62, e202217897 (2023)]. In this framework, "surface charge density" is obtained by integrating the transient photocurrent after subtracting the steady-state component. Thus, "surface charge density" represents the net amount of photogenerated charges that reach and accumulate at the semiconductor interface under illumination, rather than the dark discharge behavior typically discussed in classical capacitance measurements.

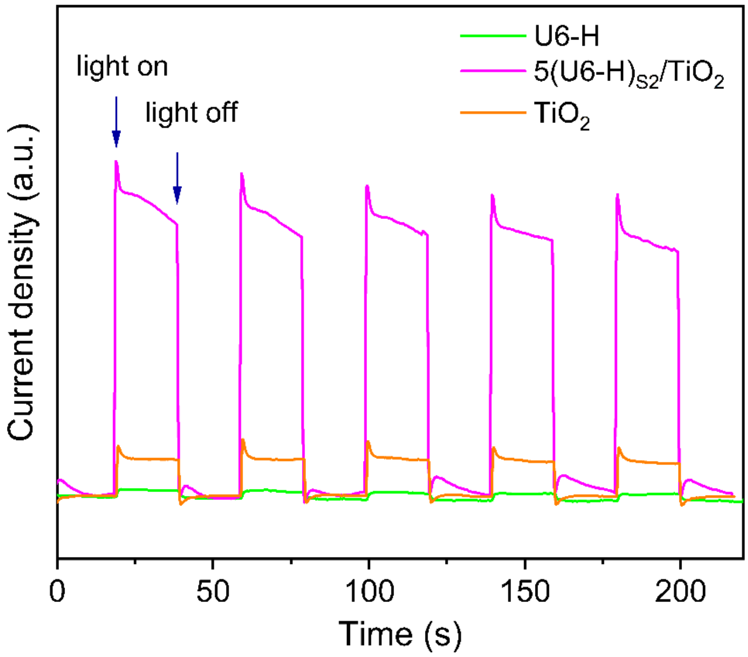

Fig. R18 the photocurrent density with light on/off cycles on U6-H, TiO_2, and 5(U6-H)$_{S2}$/TiO_2 catalysts,

Because the vertical scales of **Fig. S12** and **S13** differ (0.5 vs 0.05 mA cm^{-2} per division), the apparent magnitude of the transient and steady-state currents cannot be directly compared across the two figures. When TiO_2 and 5(U6-H)$_{S2}$/TiO_2 are plotted on the same current-density scale (**Fig. R18**), it becomes clear that the composite exhibits: (i) a much larger transient photocurrent peak, (ii) a substantially higher steady-state photocurrent, and (iii) a significantly larger integrated photocurrent area during illumination.

These features explain why the calculated "surface charge density" of 5(U6-

$H)_{S2}/TiO_2$ (58.0 μC cm^{-2}) greatly exceeds that of TiO_2 (3.98 μC cm^{-2}). Moreover, the longer dark-decay tail of 5(U6-$H)_{S2}/TiO_2$ mainly reflects its slower trap-state detrapping kinetics. Importantly, this dark-decay feature does not correlate with the amount of photogenerated interfacial charge accumulation, which is the relevant physical quantity for BEF estimation.

To avoid misunderstanding, we added this clarification in the revised manuscript (Page 12, bottom), while retaining the surface charge density analysis, as it is essential for quantifying the BEF strength.

Question 10. In Figure 2h, how the BEF intensity was calculated should be made more clear to the reader

Reply: Thank you for the comment. The BEF magnitude of prepared samples was calculated by the following equation, which has originally provided in the manuscript:

$$E = (-2V_s\rho/\varepsilon\varepsilon_0)^{1/2}$$

Where E is the internal electric field magnitude, V_s refers to the surface voltage, ρ is the surface charge density, ε stands for the low-frequency dielectric constant, and ε_0 is the permittivity of free space. The above equation indicated that the internal electric field magnitude was mainly determined by the surface charge density and the surface voltage. [Nat. Coummn., 12, 4952 (2021); Angew. Chem. Int. Ed. 62, e202217897 (2023)]

Question 11. The caption of Figure S23 should make clear what scavenger was used in each experiment.

Reply: Thank you for the suggestion. $K_2S_2O_8$, $Na_2C_2O_4$, salicylic acid, and 1,4-benzoquinone were used as quenchers for e^-, h^+, $^{\bullet}OH$, and $^{\bullet}OOH$, respectively. Accordingly, we modified the caption of updated **Fig 4b** (Page 19) in the revised manuscript and **Fig. S30** in the revised supporting information (Page 34).

Question 12. *You write, "The presence of the Type II liked band structure between TiO2*

and junctions interface prevents effectively the recombination of electron−hole pairs and boost the photocatalytic activity" From the diagram in Figure 5a, you have drawn Type I straddling gap heterojunction, not a Type II. I have now looked into your data in more detail. You have measured the work functions of your materials, in Figure S30. In my view, this work function represents the energy gap from the Fermi level of your material to vacuum, and therefore, one can create a correct band energy diagram using this data and the data in Figure S7. For your TiO2 sample, the work function is 3.36 eV vs vacuum, and therefore, on the NHE scale = 3.36 - 4.44 = -1.08 V vs NHE. And for your MOF sample, the work function is 2.66 eV vs vacuum, and therefore, on the NHE scale = 2.66 - 4.44 = -1.78 V vs NHE. Now from the XPS data in Figure S7, we know that the VBM should be 2.92 eV below the EF for TiO2, putting this at 1.84 V vs NHE and 3.56 eV below the EF for the MOF, putting this at 1.78 V vs NHE.

EF

(V vs NHE) VBM

(V vs NHE)

TiO2 -1.08 1.84

U6-H -1.78 1.78

Using this information, one can now construct a band energy diagram, which is indeed a Type II architecture, where hole carriers flow into the MOF from TiO2. Please consider this analysis in your revised version.

Reply: As discussed in our response to Question 8, following the reviewer's recommendation, we recalculated the band edge positions of all catalysts by combining the experimentally measured work functions with the band gap data to obtain the VBM values referenced to the NHE scale. Based on this approach, the revised energy band structures (**Fig. R19**) and the schematic illustrations of U6-H and TiO_2 before and after contact (**Fig. R20**) were reconstructed.

Based on this revised band alignment, the $(U6\text{-}H)_{S2}/TiO_2$ interface forms a Type-II–like heterojunction, which facilitates spatial charge separation, suppresses electron–hole recombination, and thereby enhances photocatalytic activity.

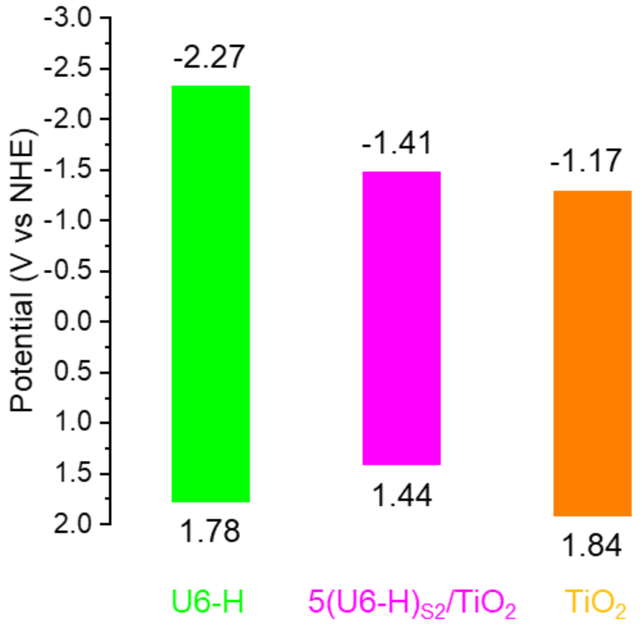

Fig. R19 Energy structure diagram for TiO_2, U6-H and 5(U6-H)$_{S2}$/TiO_2.

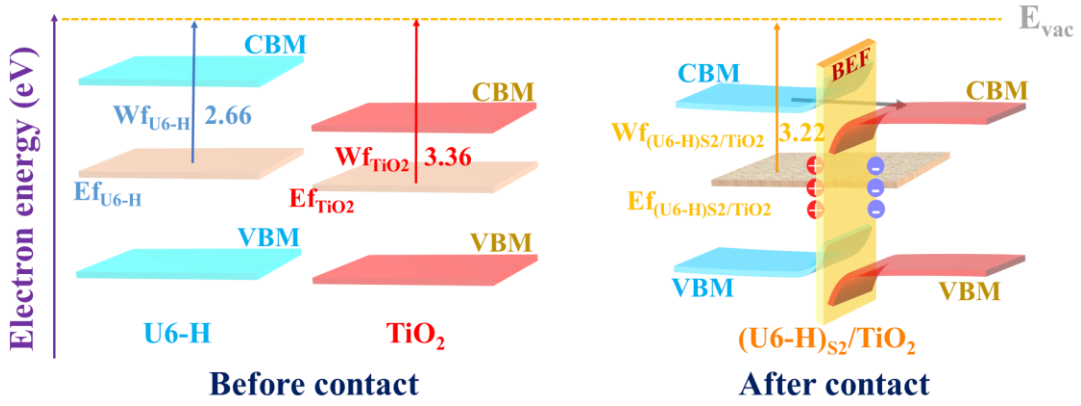

Fig. R20 Sketches of U6-H and TiO_2 before/after contact

Accordingly, we updated the energy structure diagram for TiO_2, U6-H and 5(U6-H)$_{S2}$/TiO_2 in **Fig. 2e** and Sketches of U6-H and TiO_2 before/after contact in **Fig. 5a**, respectively. We also added corresponding discussion in the revised manuscript (Page 12, top; Page 24, middle).

Reviewer 4: *In this manuscript, the authors constructed a (U6-H)S2/TiO2 interface with a robust built-in electric field (BEF) by in situ growth of sub-2 nm ultrasmall MOF*

nanocrystals on the TiO2 surface for the selective photocatalytic oxidation of methane to oxyhenates (HCHO, CH3OH, and CH3OOH)。Although the catalyst exhibits relatively superior performance, certain noteworthy issues remain unexplained in the manuscript. Therefore, I recommend publishing this paper in Nature Communications after the following revisions.

Question 1. *It is essential to explain the reasons behind the formation of sub-2nm ultra-small MOFs.*

Reply: Thank for this comment. The $(U6\text{-}H)_{S2}/TiO_2$ composite was prepared via an *in situ* growth strategy (**Fig. R21**). TiO_2 (P25) was first mixed with Zr^{4+} in DMF under stirring to form oxophilic Zr^{4+} coordinated TiO_2. Subsequently, terephthalic acid (H_2BDC) ligand was added into the above mixture to produce the $(U6\text{-}H)_{S2}/TiO_2$ composites under the hydrothermal conditions. The characteristic bands of pyridine coordinated to Lewis acid $Ti^{\delta+}$ sites on TiO_2 (1445 and 1605 cm^{-1}) (**Fig. 2b**), are markedly weakened after the introduction of ultrasmall U6-H nanocrystals. This observation indicates a significant decrease in the density of accessible Ti-based Lewis acid sites, consistent with their involvement in interfacial coordination during heterojunction formation. Attenuated total reflectance infrared (ATR-IR) spectroscopy provided deeper insight into the interaction between TiO_2 and U6-H. With the introduction of an ultrasmall U6-H nanocrystals, a new characteristic band at 432 cm^{-1} was observed (**Fig 2a**), suggesting the formation of Ti–O–Zr chemical bond.

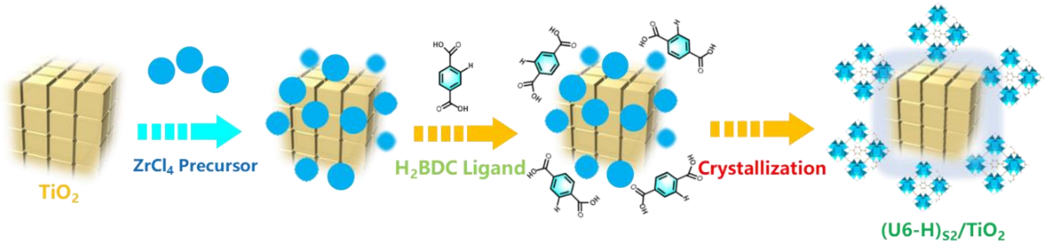

Fig. R21 Schematic illustration of TiO_2 anchored sub-2 nm U6-H via *in situ* growth method.

In the $(U6\text{-}H)/TiO_2$ composites, the characteristic peaks at $2\theta = 7.5°$ and $8.6°$ of U6-

H gradually increase with the U6-H content, confirming the coexistence of both U6-H and TiO_2 phases in the composites. High-resolution AC-HAADF-TEM images further reveal that ultrasmall MOF nanocrystals with a size of approximately < 2 nm are anchored on the surface of TiO_2 (**Fig. 1b–c, S3**). Moreover, energy-dispersive X-ray spectroscopy mapping (EDX-mapping) confirms that the Zr element from $(U6\text{-}H)_{S2}$ is uniformly dispersed across the TiO_2 surface (**Fig. 1e**). The electronic interaction between U6-H and TiO_2 in $(U6\text{-}H)_{S2}/TiO_2$ was also investigated using X-ray photoelectron spectroscopy (XPS). Compared with pristine TiO_2, the Ti 2p peak of $5(U6\text{-}H)_{S2}/TiO_2$ exhibits an apparent shift to higher binding energy, from 458.95 to 459.68 eV (**Fig. 2c**), indicating interfacial electronic coupling and charge redistribution associated with heterojunction formation. These results indicate strong interfacial coupling and the formation of a heterojunction between TiO_2 and highly dispersed sub-2 nm U6-H.

Question 2. *Please provide direct evidence demonstrating the formation of Ti-O-Zr chemical bonds.*

Reply: Thank for this comment. The attenuated total reflectance infrared (ATR-IR) spectroscopy has been used for detecting the Ti-O-Zr chemical bonds between TiO_2 and U6-H. As shown in **Fig. R22,** neither pristine U6-H nor pure TiO_2 exhibits a band in the 400–450 cm^{-1} region. With the introduction of an ultrasmall U6-H nanocrystals, a new characteristic band at 432 cm^{-1} was observed, suggesting the formation of Ti–O–Zr chemical bond.

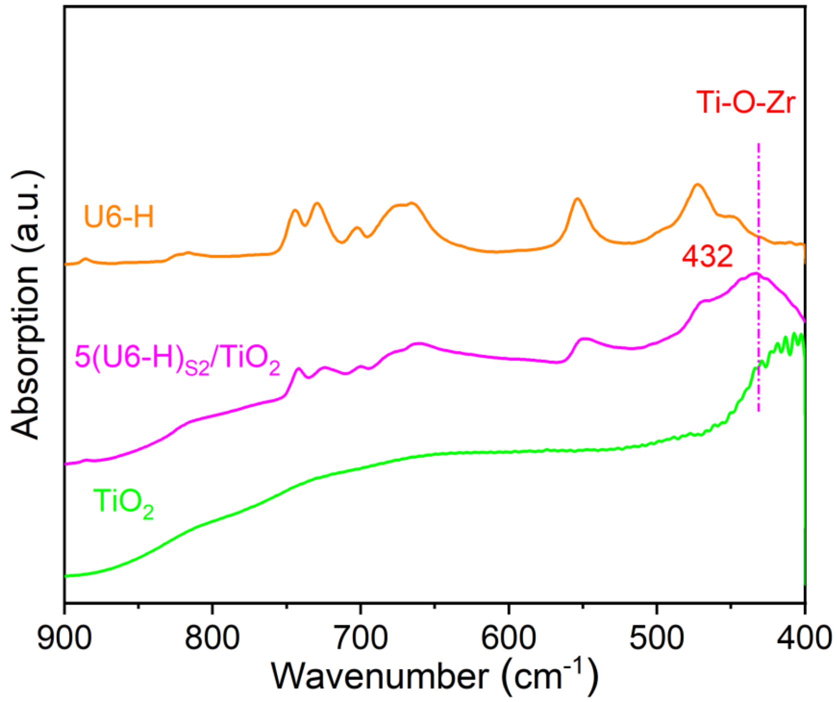

Fig. R22 ATR-IR spectra on U6-H, TiO_2 and 5(U6-H)$_{S2}$/TiO_2 catalysts

Question 3. *Figure 4a indicates that oxygenated products are formed even in the absence of air. It is therefore necessary to clarify the origin of oxygen in the oxidation products.*

Reply: Thank for this comment. $^{\bullet}OH$ can be generated on TiO_2-based catalysts in aqueous environments, as reported in previous publications [J. Am. Chem. Soc., 145, 1185 (2023)]. When we didn't use air, the oxidation products do not originate from O_2, as no CH_3OOH or other $^{\bullet}OOH$-derived species associated with O_2 reduction are detected. Therefore, the formation of CH_3OH arises from the coupling of $^{\bullet}CH_3$ with $^{\bullet}OH$, and its further oxidation leads to the formation of HCHO. However, the yield is almost 4 times lower than using CH_4 and air as reactant, which prove that the air plays a key role in photocalytic CH_4 oxidation.

Question 4. *The author claimed that "after introduction of highly dispersed sub-2 nm MOF nanocrystals onto the TiO2 surface via an in-situ growth method, the oxygenate yield increased markedly, while CO2 formation was effectively suppressed". Please explain the reasons for this phenomenon.*

Reply: Thank for this comment. The increase in oxygenate yield, while suppressing CO2 is principally interbred to generation of mild •OOH oxidizing species, and reducing the consternation of extremely active •OH radicals.

In situ liquid EPR tests were carried out by using 5, 5-dimethyl-1-pyrroline N-oxide (DMPO) as a spin trap. Before the measurements, the photocatalyst was dispersed into a solution of DMPO under atmosphere of CH_4 and air. As shown in **Fig. 4d**, a quartet signal peak of spin adducts DMPO-•OH appears after irradiation both on TiO_2 and (U6-H)$_{S2}$/TiO_2. Evidently, the •OH signal intensity for (U6-H)$_{S2}$/TiO_2 is almost 2 times weaker than that for TiO_2 **(Table R2)**. This observation is consistent with the result that, after the introduction of sub-2 nm U6-H, the VBM of (U6-H)$_{S2}$/TiO_2 shifts to a lower level compared to TiO_2 (1.84 vs. 1.44 eV, vs. NHE, **Fig. 2e**), thereby weakening its ability to oxidize H_2O into •OH. The decrease in •OH concentration coincides with an increase in selectivity toward oxygenates and a decrease in selectivity toward CO_2.

When water was replaced by methanol, the •OOH was successfully detected by *in situ* liquid EPR (**Fig. S31** and **S32**). Notably, the integral area of •OOH on 5(U6-H)$_{S2}$/TiO_2 is 2.3 times higher than that on TiO_2, indicating the favorable generation of •OOH on (U6-H)$_{S2}$/TiO_2 (**Fig. S32, Table S5**). This enhanced •OOH formation originates from the upward shift of the CBM in (U6-H)$_{S2}$/TiO_2 relative to TiO_2 (–1.41 vs. –1.17 eV, vs. NHE, **Fig. 2e**), together with the higher concentration of Ti^{3+} species **(Fig. 4c)**, both of which facilitate the reduction of O_2 to $•O_2^-$, followed by protonation to •OOH. Consequently, this pathway directly correlates with the predominance of CH_3OOH and HCHO.

These results demonstrate that the (U6-H)$_{S2}$/TiO_2 catalyst preferentially generates moderate •OOH intermediates while suppressing strong •OH oxidizing species, thereby optimizing the product distribution.

Question 5. *The author attributes the reduction in CO_2 to highly dispersed sub-2 nm MOF crystals on the surface of TiO2. However, no CO_2 was observed in the products of two control samples lacking sub-2 nm MOF crystals. Please explain the reason.*

Reply: Thank for the comment. The samples with excessive loading of U6-H contains in addition to a larger amount of sub-2 nm species, larger U6-H nanocrystals. The suppression of deep oxidation was assigned to a higher concentration of sub-2 species in the sample with a larger U6-H content.

Question 6. *To better illustrate the effect of sub-nanostructures on the built-in electric field strength, it is necessary to add the built-in electric field strengths of 10(U6-H)/TiO_2 or 20(U6-H)/TiO_2 in Figure 2h.*

Reply: Thank for the suggestion. Following this comment, we supplemented data for the 20(U6-H)$_{S2+LP}$/TiO_2 catalyst.

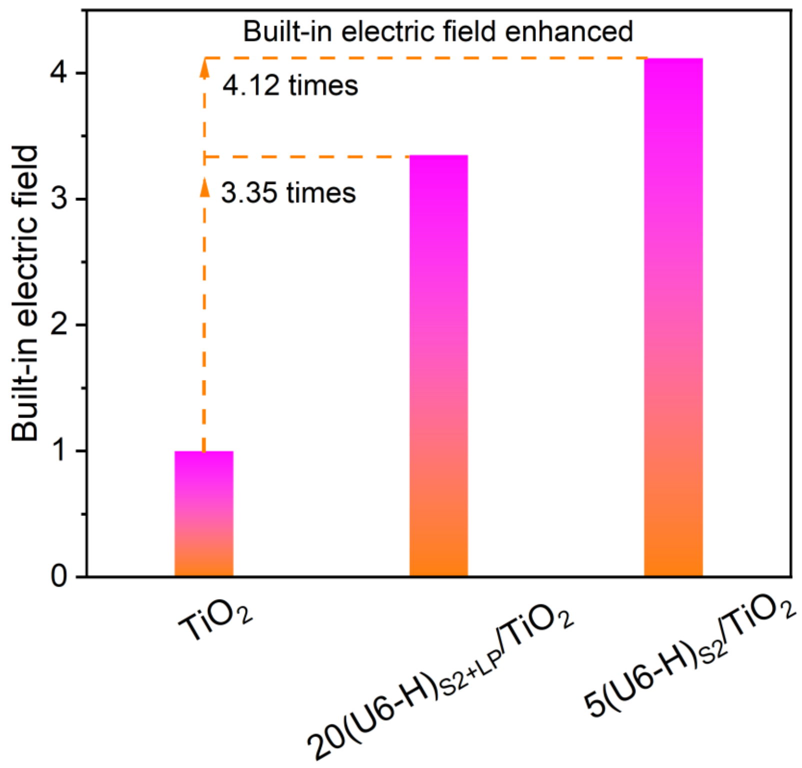

Fig. R25 The BEF of TiO_2, 5(U6-H)$_{S2}$/TiO_2, and 20(U6-H)$_{S2+LP}$/TiO_2 catalysts

Accordingly, the SPV and BEF value of 20(U6-H)$_{S2+LP}$/TiO_2 have been updated in **Fig. S15** in the revised supporting information (Page 19) and **Fig. 2h** in the revised manuscript (Page 11), respectively, while its surface charge density has been added as **Fig. S14** in the revised supporting information (Page 18).

Question 7. *The duration of each cycle in Figure 3d requires clarification.*

Reply: Thank for the comment. We have originally provided the recycling procedure in the Experimental Section for clarity. Briefly, fresh catalyst (30 mg) was first tested under standard conditions. After the reaction, the solid was recovered by filtration and dried overnight by freeze-drying. A small portion of this dried catalyst was then used for the second catalytic cycle. The remaining catalyst was subjected again to the reaction conditions, and this process was repeated to obtain a total of four cycles.

To make this clearer for readers, we have added a dedicated subsection with a bolded title describing the cycling test procedure in the revised manuscript (Page 28, bottom).

Question 8. *The interfaces play important roles and more relevant works are suggested to be referred such as 10.1016/j.apcatb.2024.124001 and 10.1016/j.apcatb.2025.125411.*

Reply: Thank for this suggestion. Accordingly, we cited the relevant works both of [10.1016/j.apcatb.2024.124001] and [10.1016/j.apcatb.2025.125411] as **Ref. 8** and **9** in the revised manuscript (Page 35).

Response to reviewers

GENERAL: We are grateful to the reviewers for all their comments. We have considered all of them carefully and have accordingly revised the manuscript. The changes are highlighted using a yellow background in Word. The detailed responses to all the critical points are listed below, with the original comments in italics.

Reviewer 1: *I have carefully reviewed the authors' revised manuscript and their detailed point-by-point responses. The authors have made a conscientious effort to address all of my previous comments, including additional evidence for charge carrier separation, supplementary 13CH4 experiments, the inclusion of AQY data, and expanded quenching studies. These revisions substantially improve both the scientific rigor and the overall completeness of the manuscript. Nevertheless, several points still require further clarification before the manuscript can be considered for acceptance. I believe that the manuscript will be suitable for publication once the authors adequately address the following issues:*

Question 1. *In Fig R6(a), the authors propose that the addition of the electron scavenger significantly suppresses radical generation and H2O2 formation, thereby inhibiting the Fenton process. This interpretation would be further strengthened if the authors could demonstrate whether adding H2O2 together with the electron scavenger restores the activity.*

Reply: Thank you for this suggestion. As shown in **Fig. R1**, when $K_2S_2O_8$ was introduced to trap photogenerated electrons, the reduction of O_2 to protonated $^{\bullet}OOH$ and H_2O_2 ($^{\bullet}OH$) species via Haber-Weiss and/or Fenton-like multistep process was significantly hindered resulting in a sharp decline in catalytic activity. This confirms that electron-mediated O_2 activation is essential for reactive oxygen species (ROS) generation under standard conditions.

As suggested, we further evaluated the photocatalytic methane oxidation performance by simultaneously introducing the electron scavenger ($K_2S_2O_8$) and a small amount of H_2O_2 (40 μL) to probe the involvement of the Fenton process.

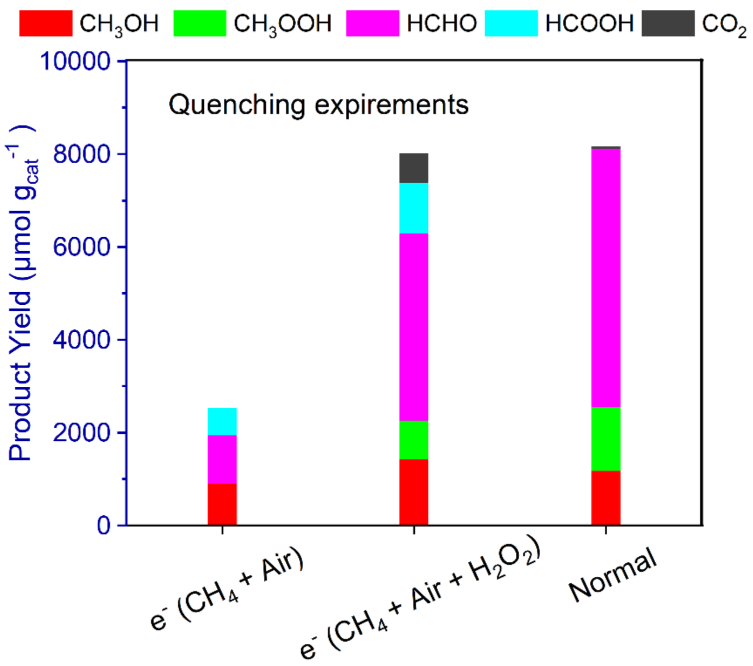

Fig. R1 Electron quenching experiments on 5(U6-H)$_{S2}$/TiO_2 catalyst, $K_2S_2O_8$ was used as quenchers for e^-. Test conditions: Pressure (CH_4) = 18 bar, (Air) = 2 bar, Catalyst: 5 mg 5(U6-H)$_{S2}$/TiO_2, 100 mL H_2O (with or without 40 μL 30 wt% H_2O_2 solution), r.t., 500 W Xe lamp illumination for 2 h

Importantly, even in the presence of $K_2S_2O_8$, the photocatalytic activity was largely restored upon the addition of H_2O_2. Compared with the O_2 proton-coupled electron transfer (PCET) pathway, which requires multiple electron-transfer steps to generate ROS and is suppressed by the electron scavenger, externally added H_2O_2 bypass the electron-dependent O_2 reduction process and more readily produce $^{\bullet}OH$ species through a Fenton-like pathway. Consequently, $^{\bullet}OH$-associated products, including CH_3OH and its deeper oxidation products HCOOH and CO_2 are relatively increased after H_2O_2 addition. Combined with the *in situ* measurements (**Fig. 4c, d**) and quenching experiments (**Fig. 4a, b, S30**), these results indicate that the Fenton radical-like pathway plays a key role in photocatalytic CH_4 oxidation over (U6-H)$_{S2}$/TiO_2.

Accordingly, we added the results of the simultaneous addition of $K_2S_2O_8$ and H_2O_2 in **Fig. S30** to the supporting information (Page 34), and added corresponding discussion to the revised manuscript (Page 18, top).

Question 2. *According to the energy structure diagram in Fig. 2e, the introduction of the MOF appears to weaken both the reduction ability of electrons and the oxidation ability of holes. The authors should clarify why the ·OOH radical concentration increases while the ·OH radical concentration decreases under these conditions.*

Reply: Thank you for this comment. After the introduction of sub-2 nm U6-H, the VBM of (U6-H)$_{S2}$/TiO_2 shifts compared to TiO_2 (1.44 eV vs. 1.84 eV versus NHE, **Fig. R2**), thereby weakening its ability to oxidize H_2O into •OH. The decrease in •OH concentration coincides with an increase in selectivity toward oxygenates and a decrease in selectivity toward CO_2.

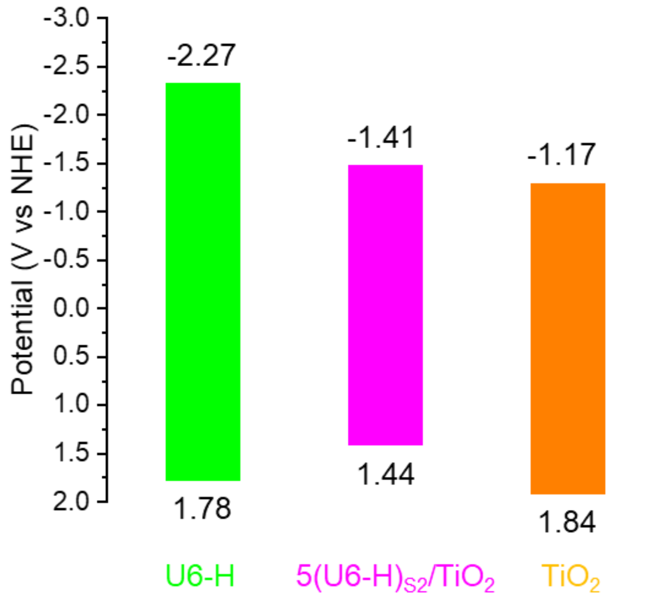

Fig. R2 Energy structure diagram for TiO_2, U6-H and 5(U6-H)$_{S2}$/TiO_2.

In contrast, the integral area of •OOH on 5(U6-H)$_{S2}$/TiO_2 detected by *in situ* liquid EPR is 2.3 times higher than that on TiO_2 (**Fig. S31** and **S32**), indicating the favourable generation of •OOH on (U6-H)$_{S2}$/TiO_2 (**Fig. S33, Table S6**). This enhanced •OOH formation originates from the shift of the CBM in 5(U6-H)$_{S2}$/TiO_2 to a more negative potential (–1.41 eV versus NHE) relative to TiO_2 (–1.17 versus NHE, **Fig. 2e**), together with the higher concentration of Ti^{3+} species (**Fig. 4c)**. Both of factors facilitate the reduction of O_2 to •O_2^-, followed by protonation to •OOH.

For clarity and transparency, we modified the corresponding description of radical

generation part in the revised manuscript (Page 21, top).

Question 3. *The energy structure diagram in the revised manuscript differs notably from that in the previous version. The authors should briefly explain the origin of this difference.*

Reply: Thank you for this comment. In the initial version, the valence band spectra were derived from XPS measurements under vacuum conditions, which directly provide the energy difference between the valence band maximum (VBM) and the Fermi level (Ef), rather than potentials versus the normal hydrogen electrode (NHE).

As suggested by Reviewer 3 (see the response to Question 8 in the previous response letter, Page 30), a consistent description of the photocatalyst band structure relative to the NHE reference requires conversion of the energy levels to the NHE scale. Therefore, the VBM versus NHE was re-estimated by combining the experimentally measured work function (Φ) with the VBM–E_f according to:

EVB versus NHE = Φ + (E_f − VBM) − 4.44.

where 4.44 eV corresponds to the energy difference between the vacuum level and the NHE reference.

Table R1. Measured work function and VBM of U6-H, TiO_2 and $5(U6\text{-}H)_{S2}/TiO_2$

Catalysts	Φ[a] (eV)	E_f – VBM (eV)	VBM vs NHE[b] (eV)
TiO_2	3.36	2.92	1.84
U6-H	2.66	3.56	1.78
$5(U6\text{-}H)_{S2}/TiO_2$	3.22	2.66	1.44

[a] Measured work function (Φ), [b] EVB versus NHE = Φ + (Ef − VBM) − 4.44.

Based on this revised analysis, the updated VBM of $5(U6\text{-}H)_{S2}/TiO_2$ is estimated to be 1.44 eV versus NHE (**Table R1**), which is lower than those of TiO_2 (1.84 eV versus NHE) and U6-H (1.78 eV versus NHE), indicating strong interfacial electronic

coupling and the formation of a heterojunction between TiO_2 and the highly dispersed sub-2 nm U6-H crystallites.

To avoid misunderstanding, the measured work function and VBM values of U6-H, TiO_2, and 5(U6-H)$_{S2}$/TiO_2 have been compiled together in **Table S2** in the supporting information (Page 42) and the corresponding discussion has been incorporated into in the revised manuscript (Page 12, top).

Reviewer 2: *The authors have carefully revised the manuscript. The revised manuscript can be considered for publication.*

Reply: We thank the reviewer for the positive evaluation and recommendation for publication. The reviewer's constructive comments have helped improve the clarity and quality of the manuscript.

Reviewer 3: *I am grateful for your detailed response to my comments and am satisfied with the changes that you have made to the manuscript. I am therefore happy to recommend this work for publication in this journal.*

Reply: We thank the reviewer for the encouraging assessment and recommendation for publication. We are pleased that the revisions have satisfactorily addressed the concerns and appreciate the valuable feedback, which has strengthened the manuscript.

Reviewer 4: *The authors have responded well to all the comments, and I recommend accepting this manuscript.*

Reply: We thank the reviewer for the positive evaluation and recommendation for acceptance. The helpful comments have contributed to improving the manuscript.
